# Dual-ligand and hard-soft-acid-base strategies to optimize metal-organic framework nanocrystals for stable electrochemical cycling performance

**DOI:** 10.1093/nsr/nwab197

**Published:** 2021-11-01

**Authors:** Shasha Zheng, Yan Sun, Huaiguo Xue, Pierre Braunstein, Wei Huang, Huan Pang

**Affiliations:** School of Chemistry and Chemical Engineering, Yangzhou University, Yangzhou 225009, China; School of Chemistry and Chemical Engineering, Yangzhou University, Yangzhou 225009, China; School of Chemistry and Chemical Engineering, Yangzhou University, Yangzhou 225009, China; Institut de Chimie UMR 7177, Universite´ de Strasbourg, CNRS, Strasbourg 67081, France; State Key Laboratory of Organic Electronics and Information Displays, and Institute of Advanced Materials (IAM), Nanjing University of Posts and Telecommunications, Nanjing 210023, China; Frontiers Science Center for Flexible Electronics (FSCFE), MIIT Key Laboratory of Flexible Electronics (KLoFE), Northwestern Polytechnical University, Xi’an 710072, China; School of Chemistry and Chemical Engineering, Yangzhou University, Yangzhou 225009, China

**Keywords:** metal-organic framework, dual ligand, hard-soft-acid-base, electrochemical energy storage, supercapacitor

## Abstract

Most metal-organic frameworks (MOFs) hardly maintain their physical and chemical properties after exposure to acidic, neutral, or alkaline aqueous solutions, resulting in insufficient stability, therefore limiting their applications. Thus, the design and synthesis of stable size/morphology-controlled MOF nanocrystals is critical but challenging. In this study, dual-ligand and hard-soft-acid-base strategies were used to fabricate a variety of 3D pillared-layer [Ni(thiophene-2,5-dicarboxylate)(4,4^′^-bipyridine)]_n_ MOF nanocrystals (1D nanofibers, 2D nanosheets and 3D aggregates) with controllable morphology by varying the concentration of 4,4^′^-bipyridine and thus controlling the crystal growth direction. Owing to the shorter ion diffusion length, enhanced electron/ion transfer and strong interactions between thiophene-2,5-dicarboxylate and 4,4^′^-bipyridine, the 2D nanosheets showed much larger specific capacitance than 1D nanofibers and 3D aggregates. A single device with an output voltage as high as 3.0 V and exceptional cycling performance (95% of retention after 5000 cycles at 3 mA cm^–2^) was realized by configuring two aqueous asymmetric supercapacitive devices in series. The excellent cycling property and charge–discharge mechanism are consistent with the hard-soft-acid-base theory.

## Teaser

A unique method combines dual-ligand with hard-soft-acid-base strategies to achieve the morphology-controlled formation of the 3D pillared-layer MOF nanocrystals, which can effectively promote the electrochemical cyclic stability.

## INTRODUCTION

Supercapacitors (SCs) have emerged as promising devices for electrochemical energy storage, on account of their long life cycle, high power density and fast charging ability [1]. Recently, 2D pseudocapacitive nanomaterials (e.g. black phosphorus [2] and transition-metal carbide/nitrides [3]) have been proven to be efficient electrode materials for high-energy and high-power-oriented applications of SCs [4]. Therefore, the synthesis and development of 2D pseudocapacitive nanomaterials is crucial for the future development of SCs.

Metal-organic frameworks (MOFs) are crystalline materials whose diverse structures, large surface areas and tunable pore sizes attract considerable attention [5,6]. Because of their remarkable properties, MOFs have been used in diverse fields, including gas storage and separation [7], batteries [8], SCs [9,10], catalysis [11,12], water treatment [13,14] and desalination [15,16]. Earlier research in this field has mainly focused on the preparation, characterization and application of bulk MOFs, and has shown considerable influence of their chemical composition, size and morphology on their functionality and utility [17,18]. In recent years, it was found that MOF nanomaterials are more promising for electrochemical energy storage devices than bulk MOF materials because of the shorter diffusion pathways and size-dependent physical–chemical properties [19,20]. However, most of these MOF nanomaterials still suffer from insufficient stability, which severely limits their application [21,22]. Consequently, the synthesis of size/morphology-controlled MOF nanocrystals with improved stability has become central to their wider application.

Although there is a growing number of methods used to regulate the size/morphology of MOFs, using different reagents and templates, most of them are only applicable to specific materials [23–25]. Therefore, it is highly desirable to develop a general and efficient method for adjusting the crystal size and morphology of different MOFs, endowed with excellent electrochemical energy storage performances. In the process of developing new methods for the crystal engineering of coordination networks, the double-ligand strategy has been shown to be effective for controlling the size, morphology and chemical properties of MOFs [26,27]. In particular, the dual-ligand strategy can provide unique pillared-layer networks to assemble specific MOFs with diverse properties [27]. Such pillared-layer networks are based on coordination bonds, which confer better structural stability and are more attractive than hydrogen bonds [28,29]. One of the reasons for the instability of many MOF crystals may be the combination of soft metal cations (Co^2+^, Zn^2+^, Ni^2+^, Cu^2+^, etc.) with hard carboxylic acid ligands, which do not conform to the hard-soft-acid-base (HSAB) principle [30]. Coordination bonds involving soft metal cations with softer N-containing organic ligands are stronger than those formed with hard carboxylic acid ligands [31,32].

**Scheme 1.**
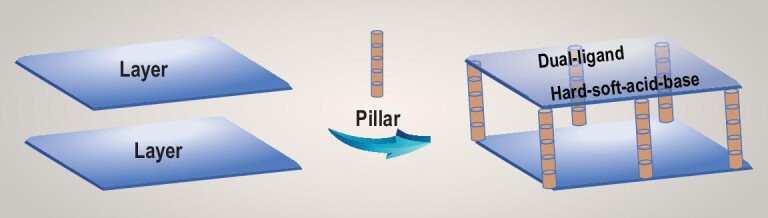
Schematic of the dual-ligand and HSAB strategies for fabricating 3D pillared-layer [Ni(Tdc)(Bpy)]_n_ MOF nanocrystals.

We decided to utilize 4,4^′^-bipyridine (Bpy) as a coordination modulator to achieve a more controllable morphology and good stability of MOFs based on dual-ligand and HSAB strategies. We report a general, rapid, room-temperature solution reaction method to fabricate a variety of 3D pillared-layer [Ni(Tdc)(Bpy)]_n_ MOF materials (Tdc = thiophene-2,5-dicarboxylate). The Ni^II^ center can be connected to the softer base Bpy to form stable 1D Ni-Bpy linear chains, which can in turn be used as pillars to support 2D Ni-Tdc network structures resulting from coordination of the Ni^II^ center with the carboxylate ligand Tdc (Scheme 1). We have successfully synthesized [Ni(Tdc)(Bpy)]_n_ MOF nanocrystals with different controllable morphologies (1D nanofibers, 2D nanosheets and 3D aggregates) by adjusting the concentration of Bpy to control the direction of crystal growth. The resulting [Ni(Tdc)(Bpy)]_n_ MOF nanocrystals were used as electrodes for SCs, and the 2D nanosheets displayed the highest specific capacity of 612 F g^–1^ at 0.5 A g^–1^. Furthermore, two aqueous asymmetric SC (ASC) devices placed in series were successfully fabricated using 2D nanosheets and activated carbon (AC), which delivers superior cycling properties.

## RESULTS AND DISCUSSION

**Figure 1.**
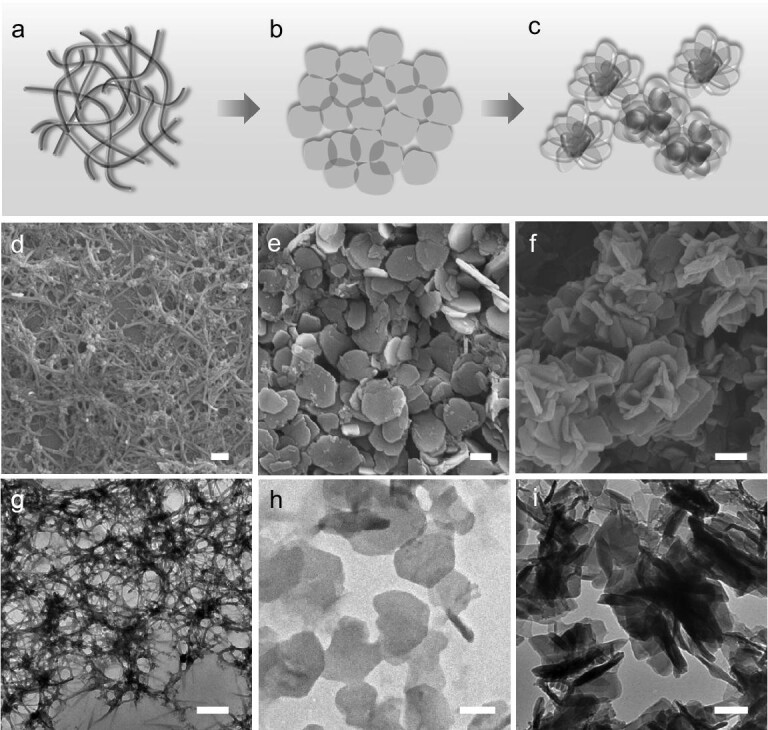
(a–c) The morphological transformation process of MOF nanomaterials from 1D nanofibers to 3D aggregates. (d–f) SEM (scale bar, 200 nm) images. (g–i) TEM (scale bar, 200 nm) images of the samples: (a, d, g) M1, (b, e, h) M5 and (c, f, i) M8.

[Ni(Tdc)(Bpy)]_n_ MOFs were readily prepared by a room-temperature alkaline solution reaction of the organic linkers Tdc and Bpy with NiCl_2_·6H_2_O. The different morphologies obtained for [Ni(Tdc)(Bpy)]_n_ according to the Tdc and Bpy molar ratios of 1:0.03, 1:0.06, 1:0.12, 1:0.25, 1:0.5, 1:1, 1:1.5 and 1:2, are denoted as M1 to M8, respectively. Scanning electron microscopy (SEM) and transmission electron microscopy (TEM) images of the [Ni(Tdc)(Bpy)]_n_ MOFs (Fig. 1) indicate that the samples M1, M5 and M8 are 1D nanofibers, 2D nanosheets and 3D aggregates with uniform morphology, respectively. The change in morphology of the MOF nanomaterials from 1D nanofibers to 3D aggregates was also observed in Figs S1 and S2. Obviously, an increasing amount of Bpy (on going from M1 to M8) results in gradual stacking and agglomeration of the 1D nanofibers to form 2D nanosheets (from M1 to M5), and then the 2D nanosheets gradually assemble to form 3D aggregates (from M6 to M8). Moreover, the high-resolution TEM images of M5 in Fig. S3 exhibit no lattice fringe, which may be due to the damage caused by the light beam at high voltage. The high-angle annular dark-field scanning TEM combined with elemental mapping show that the elements C, N, O, S and Ni are distributed throughout the M5 nanosheets (Fig. S3b). The MOF stability in water and alcohol was investigated by soaking M5 in a water or ethanol solution for 7 days. Figure S4 indicates that the structure of M5 nanosheets is destroyed in ethanol solution, but remains stable without any change in water, which is beneficial to application in aqueous SC devices. The Fourier transform infrared (FTIR) spectra of M1–M8, Tdc and Bpy are displayed in Fig. S5. For Tdc, the peaks at ∼1411, 1662 and 1522 cm^–1^ arise from the symmetric and asymmetric stretching modes of the –COOH group, respectively [33]. The peaks of the Bpy appearing at ∼1406, 1482 and 1587 cm^–1^ arise from C=C stretching. The absorptions at 800–1215 cm^–1^ are related to aromatic C–H stretching vibrations. The FTIR spectra of MOFs (M1–M8) exhibit peaks at 1566 and 1350 cm^–1^, which can be associated with the asymmetric and symmetric stretching modes of coordinated –COO^–^ groups, respectively. The difference between the peaks of the –COOH and –COO^–^ groups confirms the coordination of –COO^–^ to Ni^2+^. In addition, compared with the free ligands, a new peak at 629 cm^–1^ corresponds to Ni-O from [Ni(Tdc)(Bpy)]_n_.

**Figure 2.**
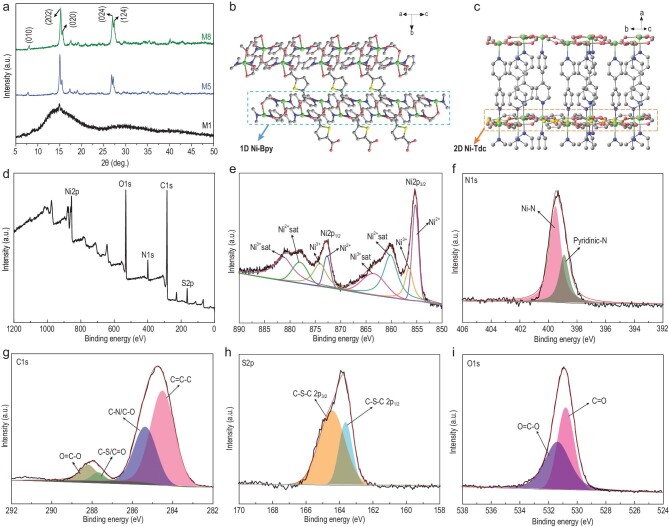
(a) XRD patterns of M1, M5 and M8. (b) The accumulation viewed along the (202) direction. (c) The accumulation viewed along the (024) direction. XPS spectra of the M5: (d) survey and high resolution, (e) Ni 2p, (f) N 1s, (g) C 1s, (h) S 2p and (i) O 1s XPS spectra.

Furthermore, X-ray diffraction (XRD) patterns confirmed that the various samples of [Ni(Tdc)(Bpy)]_n_ were successfully prepared and their characteristic peaks were indexed using the standard simulated pattern of MOFs (CCDC No. 298903) [34,35]. Figure 2a shows the XRD patterns of M1, M5 and M8, demonstrating that the 1D nanofibers (M1) preferentially grow (202) crystal planes, while 2D nanosheets and 3D aggregates have exposed crystal planes (010), (020), (024) and (124). Interestingly, upon stacking of 1D nanofibers and agglomeration to 2D nanosheets (from M1 to M5), the (024) crystal plane is gradually exposed and the diffraction peak intensity increases (Fig. S6). To further explain this phenomenon, the molecular structure of a [Ni(Tdc)(Bpy)]_n_ MOF, which belongs to the orthorhombic space group (*Pccn*), was analyzed [34]. Figure S7a shows that each Ni^II^ center is coordinated by four oxygen atoms and two nitrogen atoms, and the Ni^II^ center is in a six-coordinated environment, forming an octahedral structure [36]. In the 3D stacking structure of a [Ni(Tdc)(Bpy)]_n_ MOF, the Ni^II^ ions connect to the N atoms of Bpy to construct 1D Ni-Bpy linear chains, and these 1D chains serve as ‘pillars’ to support the 2D Ni-Tdc network structures formed by coordination of the carboxyl oxygen atoms of Tdc to the Ni^II^ center (Fig. S7b–e) [37]. A view along the (202) direction (Fig. 2b) shows that adjacent 1D Ni-Bpy linear chains are bridged by Tdc to further extend into a 3D porous structure through Ni-O bonds. Therefore, when a small amount of Bpy is introduced, the 1D Ni-Bpy linear chains form and control the growth of [Ni(Tdc)(Bpy)]_n_ MOF crystals along the (202) crystal plane as a priority, making M1 appear as 1D nanofibers. With an increasing amount of Bpy, the 1D nanofibers gradually stack and agglomerate to form 2D nanosheets, the crystal planes (010), (020), (024) and (124) are exposed, and the perspective views along the direction of these crystal planes (Figs 2c and S8) indicate that the adjacent 2D Ni-Tdc network structures are bridged by Bpy to further extend into a 3D porous structure by Ni-N bonds, resulting in the formation of 2D nanosheets.

X-ray photoelectron spectroscopy (XPS) measurements confirm that M5 contains five elements: Ni, N, C, S and O (Fig. 2d). The Ni 2p spectrum demonstrates the presence of two types of Ni species: Ni^2+^ at 855.2 and 872.8 eV and Ni^3+^ at 856.5 and 874.2 eV (Fig. 2e) [38,39]. The deconvoluted N 1s XPS spectrum (Fig. 2f) indicates peaks at 399.5 and 398.8 eV arising from Ni-N and pyridinic N [40]. In the C 1s XPS spectrum (Fig. 2g), the peaks appearing at ∼288.2, 287.6, 285.3 and 284.5 eV arise from O=C–O, C–S/C=O, C–N/C–O and C=C–C, respectively [41]. Moreover, the existence of O=C–O (531.3 eV) and C=O (530.7 eV) units is further verified by the O 1s spectrum (Fig. 2i) [41]. The S 2p spectrum (Fig. 2h) reveals the presence of C–S–C, further confirming the formation of [Ni(Tdc)(Bpy)]_n_ [42]. Furthermore, the XPS spectra of other samples also indicated that the constructed MOF materials are [Ni(Tdc)(Bpy)]_n_ (Figs S9–S15). To study the porous structure of the MOF materials, the Brunauer-Emmett-Teller (BET) surface areas were determined. A sample of M5 exhibited a high specific surface area that was remarkably larger than those of other MOF materials (Fig. S16 and Table S2). Furthermore, the pore size distribution also indicates the coexistence of micropores and mesopores in the MOF materials, with M5 possessing a larger pore volume (Fig. S17). Therefore, the high specific surface area of M5 is favorable for promoting electrolyte permeation to access more redox active sites.

**Figure 3.**
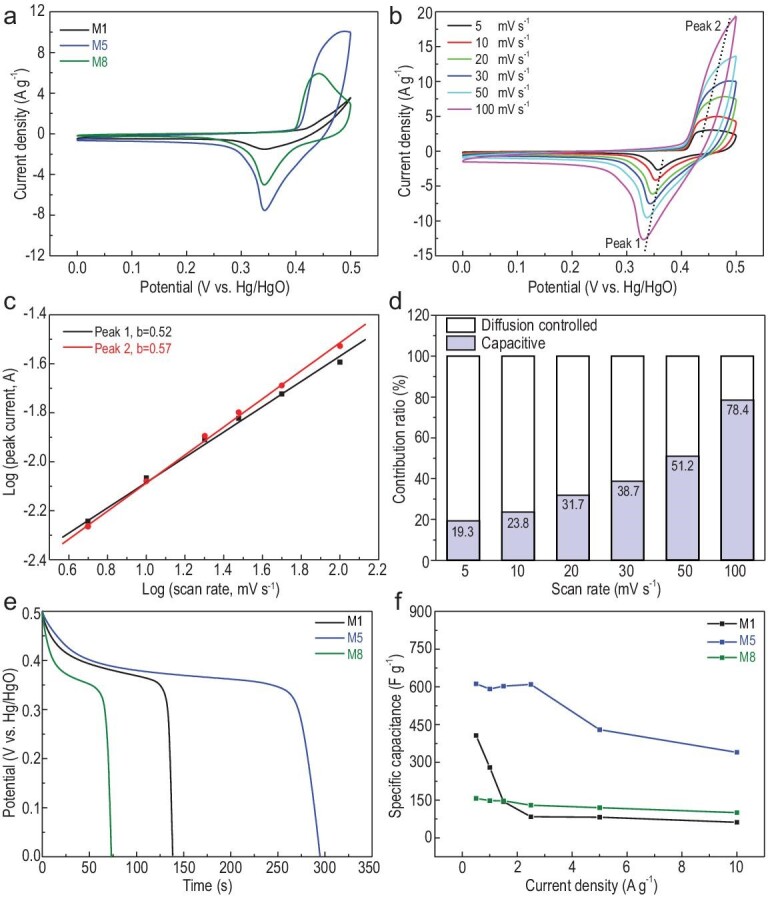
(a) CV curves of the M1, M5 and M8 at 30 mV s^–1^ in a three-electrode cell. (b) CV curves for M5 at various scan rates. (c) Log*(i)* versus log*(v)* plots for M5. (d) Bar chart showing the % of capacitive contribution of M5 at various scan rates. (e) Galvanostatic discharge curves of M1, M5 and M8 at 1 A g^–1^. (f) Specific capacitance of M1, M5 and M8 at multiple current densities.

The electrochemical capacitive properties of the samples M1–M8 were first studied in a three-electrode system. The SEM images of the prepared MOF electrodes show that the morphology of 1D nanofibers, 2D nanosheets and 3D aggregates is well maintained during the fabrication process of the electrodes (Fig. S18). The cyclic voltammetry (CV) curves in Fig. 3a reveal that the redox current density of M5 is much higher than that of the other electrodes. The electrochemical reaction kinetics of the as-fabricated electrodes were further studied via CV at multiple scan rates (Figs 3b, S19a, S21a, S23a, S25a, S28a, S30a and S32a). As the scan rates increase, the reduction peak (peak 1) moves to a more negative voltage and the oxidation peak (peak 2) shifts to a more positive voltage, which may be caused by an increase in the internal diffusion resistance at high scan rates. Even at high scan rates, the redox peaks of M3–M5 are well maintained, indicating their excellent capacitive behavior and rate capability. Furthermore, the relationship between current (i) and scanning rate (v) is *i = av^b^*(details in the Supplementary Data) [43]. The *b* values are between 0.5 and 0.7, indicating that both diffusion-controlled and surface capacitive-controlled processes exist in the entire electrochemical reaction (Figs 3c, S19b, S21b, S23b, S25b, S28b, S30b and S32b). Subsequently, the ratios of the two capacitive mechanism contributions at various scan rates can be calculated (details in the Supplementary Data). As the scan rate increases, the capacitive contribution shows an increasing trend, indicating high-efficiency charge storage (Figs 3d, S19c, S20, S21c, S22, S23c, S24, S25c, S26, S27, S28c, S29, S30c, S31, S32c and S33). As shown in Fig. S34, compared with other electrodes, M5 has a higher diffusion-controlled contribution, which means that the corresponding redox reactions in the M5 electrode are mainly diffusion-controlled processes. Figure S35a shows CV curves for the M5 electrode at various potentials. As the potential window increases, the area delimited by the corresponding CV curve increases accordingly. To further explore the electrochemical behavior of M1–M8, galvanostatic charge–discharge (GCD) tests were performed. In Fig. S35b and c, the GCD and specific capacitance change versus potential curves of the M5 electrode at different potentials suggest that this electrode possesses the longest charge–discharge time and highest specific capacitance at a charge–discharge potential of 0.5 V. From the GCD curves in Fig. 3e at 1 A g^–1^, M5 displays the longest discharge time compared with M1 and M8. The GCD curves of the other electrodes at various current densities are displayed in Figs S36 and S37. As shown in Fig. S38, although M3 has a longer discharge time than M5, the coulombic efficiency and rate performance of M3 are relatively poor. Overall, M5 exhibits the highest specific capacity (592 F g^–1^ at 1 A g^–1^), and its coulombic efficiency is also good (Fig. 3f). To highlight the outstanding electrochemical capacitive performance of M5, a comparison with recently reported MOF nanomaterials [44–46] is presented in Table S3. For comparison, the electrochemical capacitive properties of the organic ligands (Bpy, Tdc, Bpy/Tdc) were also tested (Fig. S39). The organic ligand electrodes exhibit almost no electrochemical energy storage characteristics, which indicates that the [Ni(Tdc)(Bpy)]_n_ MOFs formed by the organic ligands and Ni^2+^ have unique energy storage characteristics. Their superior electrochemical activity was further confirmed via electrochemical impedance spectroscopy (EIS) measurements (Fig. S40). The intersection point of the Nyquist plot with the Z′ axis represents R_s_ (series resistance). M5 exhibits a low value of R_s_ at 1.06 Ω, similar to M1 (2.28 Ω), M2 (2.20 Ω), M3 (0.70 Ω), M4 (0.92 Ω), M6 (1.48 Ω), M7 (1.02 Ω) and M8 (0.93 Ω). The results indicate that the as-obtained electrodes have high electronic conductivity and rapid reaction kinetics.

**Figure 4.**
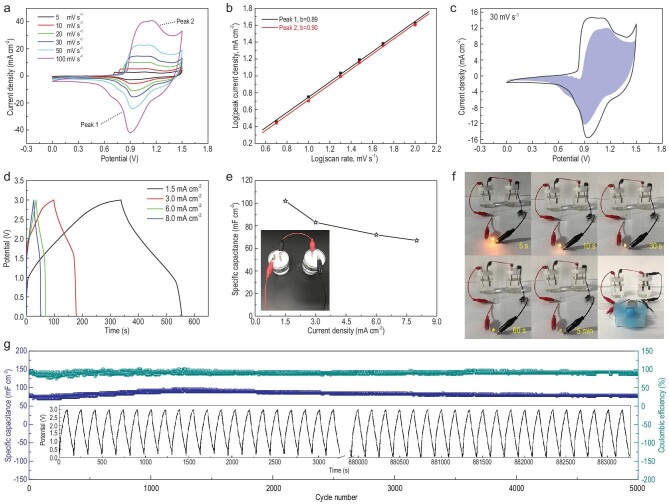
(a) CV curves of M5//AC at multiple scan rates. (b) Log*(i)* versus log*(v)* plots for M5//AC. (c) CV curve with the capacitive fraction displayed by the shaded area of M5//AC at 30 mV s^–1^. (d) GCD curves of two M5//AC devices linked in series at multiple current densities. (e) Specific capacitance changes versus current density of two M5//AC devices linked in series. (Inset) Optical image of two M5//AC devices linked in series. (f) Schematic illustration of two M5//AC devices linked in series to light up a yellow LED and power a rotating motor. (g) Cycling performance and coulombic efficiency at 3 mA cm^–2^ for 5000 cycles. (Inset) The first 20 and last 20 GCD curves of two M5//AC devices linked in series.

ASC devices were fabricated from [Ni(Tdc)(Bpy)]_n_ MOFs (positive) and AC (negative) materials using our previously reported method (at a mass ratio of 1:1.8, details are in the Supplementary Data, Fig. S41). As displayed in Fig. 4a, the shape of the CV curves of the M5//AC ASC device can be maintained at various scan rates, suggesting typical pseudocapacitive performance with rapid and reversible charge storage capacity. To better understand the enhanced properties of the M5//AC ASC device, the interfacial capacitive behavior of all ASC devices was further confirmed via CV tests at multiple scan rates. By plotting log(i) versus log(v), *b* values of M1–M8//AC ASC devices were found to be between 0.8 and 1 (Figs 4b, S42–S57). This result indicates that the overall charge storage by M1–M8//AC ASC devices can be divided into diffusion-controlled and surface capacitance-controlled processes but are mainly due to a surface capacitance-controlled process. Figure S58 shows that the surface capacitance-controlled contribution in the M5//AC ASC device is more significant. With an increasing scan rate, the surface capacitance-controlled contribution rate of M5//AC gradually increases from 52.8% to 94.8%, indicating that M5//AC has good charge-transfer kinetics, which is also the reason why M5//AC has high rate capability. A shaded area related to the surface capacitance-controlled contribution in M5//AC accounts for a large fraction of 74.2% at 30 mV s^–1^ (Fig. 4c). As displayed in Fig. S59, the specific capacitance of the as-constructed M5//AC ASC devices is the highest at 1.5 V. Additionally, the GCD curves of the assembled M1–M8//AC ASC devices were tested at different current densities (Figs S60 and S61). In Fig. S62, the M5//AC ASC device reveals a specific capacitance of 207 mF cm^–2^, which is high compared to other devices (the values for M1, M2, M3, M4, M6, M7 and M8 are 98.5, 155, 90, 106, 119, 74 and 61 mF cm^–2^, respectively), mainly because 2D MOF nanosheets have a shorter ion transport pathway (Fig. S63). At 8 mA cm^–2^, the M5//AC ASC device provides an excellent rate capability by maintaining a capacitance of 144 mF cm^–2^. To further explore the impedance performance, EIS analysis was performed, and the Nyquist plots of the M1–M8//AC ASC devices are displayed in Fig. S64. The charge-transfer resistance (R_ct_) of this device was calculated using ZView software. The M5//AC ASC device revealed a low R_s_ of 1.2 Ω and an R_ct_ of 79 Ω. The low resistance value for the M5//AC ASC device indicates easy ion diffusion. In addition, the GCD curves of the device at different mass loadings were investigated. The specific capacitance was found to gradually increase with the mass loading, but with the higher mass loading (8 mg cm^–2^), the specific capacitance decreases, probably because the active material on the electrode is not fully utilized under the high mass loading conditions (Fig. S65).

To explore the practical applications of the ASC device, two M5//AC ASC devices were linked in series. Figure 4d shows GCD curves for two M5//AC ASC devices linked in series. This device could be extended to a large voltage window of 3.0 V. As displayed in Fig. 4e, the specific capacitance of the series device at 1.5, 3, 6 and 8 mA cm^–2^ was calculated to be 102, 83, 72 and 67 mF cm^–2^, respectively, showing good rate capability. According to the GCD curves in Fig. 4d, we calculated the coulombic efficiency of the device at different current densities (Fig. S66). With the increase of current density, the coulombic efficiency gradually increases, and is close to 100% at 8.0 mA cm^–2^. More importantly, in Fig. 4f, this series device was used to power a yellow LED and a rotating motor for ∼5 min and 3 s, respectively, after charging for 1 min (Videos S1 and S2). Moreover, this device demonstrates an excellent cycling property with a capacitance retention of 95% and an excellent coulombic efficiency of 96% at 3 mA cm^–2^ after 5000 cycles, which should be of benefit for fast-charging energy storage devices (Fig. 4g).

**Figure 5.**
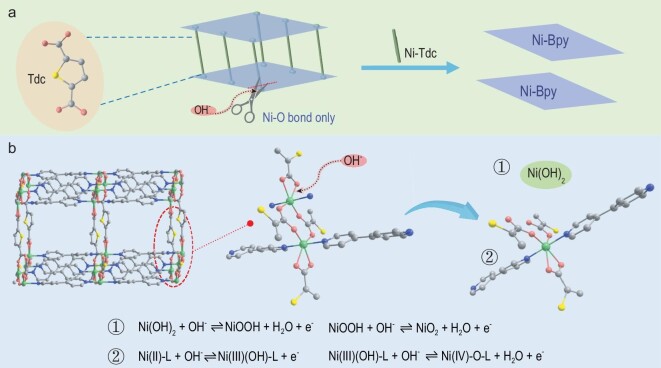
(a) Schematic for the selective removal of Tdc carboxylate links from [Ni(Tdc)(Bpy)]_n_ nanosheets by OH^–^ in the electrolyte during charging and discharging. (b) Mechanism of charge/discharge of a [Ni(Tdc)(Bpy)]_n_ MOF-based electrode.

To further investigate the charge–discharge mechanism of the electrode materials, SEM images of M5 after cycling were obtained (Fig. S67). The SEM images show that the nanosheet morphology of M5 is well maintained without visible damage. The corresponding elemental mapping images of M5 after cycling indicate that C, N, O, S, Ni and K are distributed throughout the whole nanosheet (Fig. S68). The XRD patterns show that the (202) crystal plane of M5 is retained and the other crystal planes disappear after cycling (Fig. S69). This phenomenon can be explained by the selective removal of Tdc carboxylate links from [Ni(Tdc)(Bpy)]_n_ nanosheets by OH^–^ in the electrolyte during charging and discharging, whereas the Ni-Bpy layers are well protected (Fig. 5a). Therefore, the (202) crystal plane corresponding to the Ni-Bpy layers is well preserved. In addition, Fig. S70 shows that the XPS spectral peaks of N 1s and C 1s decrease after cycling, and the XPS spectral peak of S 2p almost disappears, which also confirms the above hypothesis. As can be seen from the cycling performance (Fig. 4g), the specific capacitance slowly increases before 1000 cycles, which may be due to the capacity contribution of Ni(OH)_2_ generated during the removal of Tdc carboxylate linkers. Therefore, the possible charge–discharge mechanism is shown in Fig. 5b.

## CONCLUSIONS

In conclusion, we have proposed a facile method for the preparation of morphology-controllable 3D pillared-layer [Ni(Tdc)(Bpy)]_n_ MOF nanocrystals, based on the synergetic dual-ligand and HSAB strategies. The nanocrystals are revealed as efficient electrode materials for SCs with excellent life cycles. Bpy was found to act as a coordination modulator to adjust the morphology transformation of [Ni(Tdc)(Bpy)]_n_ MOF nanocrystals from 1D nanofibers to 2D nanosheets and then to 3D aggregates. This appears related to the amount of Bpy introduced, which causes MOF crystals to grow along 1D Ni-Bpy linear chains or in a 2D Ni-Tdc network direction. Compared with the 1D nanofibers and 3D aggregates, the 2D nanosheets demonstrate higher electrochemical performance. This is closely related to the ion diffusion and charge-transfer processes. The excellent electrochemical properties of 2D nanosheets are due to their short ion transport distance. Furthermore, by way of HSAB strategies, in the 3D pillared-layer [Ni(Tdc)(Bpy)]_n_ MOF structure the Ni^II^ center can be used as a soft metal site to connect with the soft base Bpy to construct a stable 1D Ni-Bpy linear chain. During the charging and discharging process, the Ni-Tdc network in the MOF was removed by OH^–^ in the electrolyte, while the Ni-Bpy layer was well protected, thus providing good cycling stability. We believe that this work can provide a general approach to designing size/morphology-controllable and functional adjustable MOFs.

## Supplementary Material

nwab197_Supplemental_Files
