# Supplementary material for: Dual-ligand and hard-soft-acid-base strategies to optimize metal-organic framework nanocrystals for stable electrochemical cycling performance
Source: Natl Sci Rev. 2021 Nov 1;9(7):nwab197. doi: 10.1093/nsr/nwab197 (PMC9362764; doi:10.1093/nsr/nwab197)
Supplement: nwab197_Supplemental_Files [file nwab197_supplemental_files.zip › Supporting_Information.docx]

**Supporting Information**

**Dual-ligand and hard-soft-acid-base strategies to optimize metal-organic framework nanocrystals for stable electrochemical cycling performance**

Shasha Zheng,^1^ Yan Sun,^1^ Huaiguo Xue,^1^ Pierre Braunstein,^4^ Wei Huang^2,3^* and Huan Pang^1,^*

^1^School of Chemistry and Chemical Engineering, Yangzhou University, Yangzhou, 225009, Jiangsu, China.

^2^State Key Laboratory of Organic Electronics and Information Displays & Institute of Advanced Materials (IAM), Nanjing University of Posts & Telecommunications, 9 Wenyuan Road, Nanjing 210023, China.

^3^Frontiers Science Center for Flexible Electronics (FSCFE), MIIT Key Laboratory of Flexible Electronics (KLoFE), Northwestern Polytechnical University, Xi'an 710072, China.

^4^Universite´ de Strasbourg, CNRS, Institut de Chimie UMR 7177, Strasbourg 67081, Cedex, France.

E-mail: iamwhuang@njupt.edu.cn; panghuan@yzu.edu.cn

Content

**1.** **Experimental section** 3

**1.1** **Materials** 3

**1.2** **Materials synthesis** 3

**1.3** **Material characterization** 4

**1.4** **Fabrication of the electrodes in a traditional three-electrode system** 4

**1.5** **Fabrication of the aqueous electrochemical energy storage device** 4

**2.** **Calculations** 6

**3.** **SEM images** 7

**4.** **TEM images** 8

**5.** **HRTEM images of M5** 9

**6.** **The stability of M5 in water and alcohol** 10

**7.** **IR patterns** 11

**8.** **XRD patterns** 12

**9.** **Structure of [Ni(Tdc)(Bpy)]_n_ MOF** 13

**10.** **XPS spectra of the M1** 15

**11.** **XPS spectra of the M2** 16

**12.** **XPS spectra of the M3** 17

**13.** **XPS spectra of the M4** 18

**14.** **XPS spectra of the M6** 19

**15.** **XPS spectra of the M7** 20

**16.** **XPS spectra of the M8** 21

**17.** **BET** 22

**18.** **Table S2. BET surface area** 23

**19.** **Pore size distribution** 24

**20.** **SEM images of M1, M5, and M8 electrode** 25

**21.** **The analysis of ion-diffusion and capacitive contributions of the M1** 26

**22.** **Pseudocapacitive contribution shadow diagram of M1 in CV curves** 27

**23.** **The analysis of ion-diffusion and capacitive contributions of the M2** 28

**24.** **Pseudocapacitive contribution shadow diagram of M2 in CV curves** 29

**25.** **The analysis of ion-diffusion and capacitive contributions of the M3** 30

**26.** **Pseudocapacitive contribution shadow diagram of M3 in CV curves** 31

**27.** **The analysis of ion-diffusion and capacitive contributions of the M4** 32

**28.** **Pseudocapacitive contribution shadow diagram of M4 in CV curves** 33

**29.** **Pseudocapacitive contribution shadow diagram of M5 in CV curves** 34

**30.** **The analysis of ion-diffusion and capacitive contributions of the M6** 35

**31.** **Pseudocapacitive contribution shadow diagram of M6 in CV curves** 36

**32.** **The analysis of ion-diffusion and capacitive contributions of the M7** 37

**33.** **Pseudocapacitive contribution shadow diagram of M7 in CV curves** 38

**34.** **The analysis of ion-diffusion and capacitive contributions of the M8** 39

**35.** **Pseudocapacitive contribution shadow diagram of M8 in CV curves** 40

**36.** **The percent of the diffusion-controlled capacity and pseudocapacitive contribution of the M1-M8** 41

**37.** **CV and GCD curves of the M5 at different potentials** 42

**38.** **The GCD curves of M1-M4 at different current densities** 43

**39.** **The GCD curves of M5-M8 at different current densities** 44

**40.** **The specific capacitance of the M1-M8 at different current densities.** 45

**41.** **The specific capacitance of organic ligand** 46

**42.** **The electrochemical impedance spectra of M1-M8** 47

**43.** **The GCD curves of AC** 48

**44.** **The analysis of ion-diffusion and capacitive contributions of the M1//AC** 49

**45.** **Pseudocapacitive contribution shadow diagram of M1//AC in CV curves** 50

**46.** **The analysis of ion-diffusion and capacitive contributions of the M2//AC** 51

**47.** **Pseudocapacitive contribution shadow diagram of M2//AC in CV curves** 52

**48.** **The analysis of ion-diffusion and capacitive contributions of the M3//AC** 53

**49.** **Pseudocapacitive contribution shadow diagram of M3//AC in CV curves** 54

**50.** **The analysis of ion-diffusion and capacitive contributions of the M4//AC** 55

**51.** **Pseudocapacitive contribution shadow diagram of M4//AC in CV curves** 56

**52.** **The analysis of capacitive contributions of the M5//AC** 57

**53.** **Pseudocapacitive contribution shadow diagram of M5//AC in CV curves** 58

**54.** **The analysis of ion-diffusion and capacitive contributions of the M6//AC** 59

**55.** **Pseudocapacitive contribution shadow diagram of M6//AC in CV curves** 60

**56.** **The analysis of ion-diffusion and capacitive contributions of the M7//AC** 61

**57.** **Pseudocapacitive contribution shadow diagram of M7//AC in CV curves** 62

**58.** **The analysis of ion-diffusion and capacitive contributions of the M8//AC** 63

**59.** **Pseudocapacitive contribution shadow diagram of M8//AC in CV curves** 64

**60.** **The percent of the diffusion-controlled capacity and pseudocapacitive contribution of the M1-M8//AC** 65

**61.** **CV and GCD curves of the M5//AC at different potentials** 66

**62.** **The GCD curves of M1-M4//AC at different current densities** 67

**63.** **The GCD curves of M5-M8//AC at different current densities** 68

**64.** **The specific capacitance of the M1-M8//AC at different current densities** 69

**65.** **Schematic diagram of ion transport pathway** 70

**66.** **The electrochemical impedance spectra of M1-M8//AC** 71

**67.** **The GCD curves of M5 with different mass loadings** 72

**68.** **The coulombic efficiency** 73

**69.** **SEM images of M5 after cycling** 74

**70.** **The EDS mapping images of M5 after cycling** 75

**71.** **XRD patterns of M5 after cycling** 76

**72.** **XPS spectra of M5 after cycling** 77

**73.** **A comparison with previously reported MOF nanomaterials.** 78

**74.** **References** 79

1. **Experimental section**
   1. **Materials**

All chemicals, thiophene-2,5-dicarboxylate (Tdc, C_6_H_2_O_4_S, 98%, Aladdin Reagent), 4,4’-bipyridine (Bpy, C_10_H_8_N_2_, 98%, Aladdin Reagent), nickel chloride hexahydrate (NiCl_2_·6H_2_O), polyvinyl pyrrolidone (PVP) and sodium hydroxide (NaOH, 96%), were purchased and used without further purification. All aqueous solutions were freshly prepared with deionized water (18 MΩ cm).

- 1. **Materials synthesis**

**Synthesis of [Ni(Tdc)(Bpy)]_n_.** In a typical synthesis, 29.6 mg Bpy, 56 mg Tdc and 10 mg PVP were first dissolved in 15 mL of deionized water in an ultrasonic bath for 15 min at 25 ^o^C. Then, 5 mL of aqueous NaOH (26.4 mg NaOH) was slowly added dropwise to the solution and 5 mL of deionized water containing 23.8 mg NiCl_2_·6H_2_O was slowly added to the reaction mixture. After it was magnetically stirred for 20 min, a precipitate was formed and collected by centrifugation and thoroughly washed several times with deionized water and alcohol. Then, the precipitate was dried in the air at 25 ^o^C. The different [Ni(Tdc)(Bpy)]_n_ synthesized using Tdc and Bpy in a molar ratio of 1 : 0.03, 1 : 0.06, 1 : 0.12, 1 : 0.25, 1 : 0.5, 1 : 1, 1 : 1.5 and 1 : 2, are denoted M1, M2, M3, M4, M5, M6, M7, and M8, respectively (**Table S1**).

**Table S1. Experimental parameters for the synthesis of the samples.**

| **Sample** | **Chemical agents** | | | | | | **Magnetic stirring** | |
| --- | --- | --- | --- | --- | --- | --- | --- | --- |
|  | **NiCl_2_·6H_2_O** | **Bpy** | **Tdc** | **PVP** | **NaOH** | **H_2_O** | **T(^o^C)** | **Time** |
| M1 | 23.8 mg | 1.8 mg | 56 mg | 10 mg | 26.4 mg | 25 mL | RT | 20 min |
| M2 | 23.8 mg | 3.7 mg | 56 mg | 10 mg | 26.4 mg | 25 mL | RT | 20 min |
| M3 | 23.8 mg | 7.4 mg | 56 mg | 10 mg | 26.4 mg | 25 mL | RT | 20 min |
| M4 | 23.8 mg | 14.8 mg | 56 mg | 10 mg | 26.4 mg | 25 mL | RT | 20 min |
| M5 | 23.8 mg | 29.6 mg | 56 mg | 10 mg | 26.4 mg | 25 mL | RT | 20 min |
| M6 | 23.8 mg | 59.2 mg | 56 mg | 10 mg | 26.4 mg | 25 mL | RT | 20 min |
| M7 | 23.8 mg | 88.8 mg | 56 mg | 10 mg | 26.4 mg | 25 mL | RT | 20 min |
| M8 | 23.8 mg | 118.4 mg | 56 mg | 10 mg | 26.4 mg | 25 mL | RT | 20 min |

- 1. **Material characterization**

The morphological features were characterized by field emission scanning electron microscopy (FESEM, Zeiss-Supra55), transmission electron microscopy (TEM, JEM-2100). X-ray diffraction (XRD) patterns were examined on a Bruker D8 Advanced X-ray Diffractometer (Cu-K*α* radiation: *λ* = 0.15406 nm). The chemical states were measured using an Axis Ultra X-ray photoelectron spectroscope (XPS, Kratos Analytical Ltd., UK) equipped with a standard monochromatic Al-K*α* source (hv = 1486.6 eV). Fourier transform infrared (FTIR) transmission spectra were obtained on a BRUKER-EQUINOX-55 IR spectrophotometer.

- 1. **Fabrication of the electrodes in a traditional three-electrode system**

Electrochemical performance was conducted by a CHI 660E instrument in a traditional three-electrode system. A Hg/HgO electrode and platinum electrode were chosen as reference and counter electrode, respectively. The electrolyte was 3.0 M KOH aqueous solution. The working electrode was prepared by grinding the mixture of active materials, acetylene black, and polytetrafluoroethylene (PTFE) with a weight ratio of 80:15:5, and coating the mixture on a 1 cm × 5 cm nickel foam. The additive was a certain amount of isopropyl alcohol when grinding and the painted size was about 1 cm^2^. In addition, we pressed the nickel foam to a thin foil with a pressure of 10.0 MPa. The typical mass loading of the electrode material was ≈2 mg.

- 1. **Fabrication of the aqueous electrochemical energy storage device**

Aqueous electrochemical energy storage devices were assembled by employing the MOF-based materials as positive electrode and activated carbon as negative electrode. The mass loading for the negative electrode was determined by balancing the charges stored in each electrode. Generally, the charges stored by positive and negative electrodes can be determined by q_+_ = C_+_ × △E_+_ × m_+_ and q_-_ = C_-_ × △E_-_ × m_-_, where C_+_, C_-_ represent the specific capacitance of positive electrode and negative electrodes (F g^-1^), respectively; △E is the potential range (V); m_+_, m_-_ is the weight of the active material in positive electrode and negative electrode (g), respectively; The charges are balanced by the equation of q_+_ = q_-_, where q_+_ and q_-_ represent the charges stored in the positive and negative electrodes, respectively. Therefore, m_+_/m_-_ = C_-_ × △E_-_/C_+_ × △E_+_. The specific capacitance of purchased activated carbon electrode was 202.5 F g^-1^ when the current density was 1 A g^-1^ (**Figure S41**). Thus, the mass ratio between the positive and negative electrodes was set at 1:1.8 in the as-assembled device. The electrochemical performance of the devices was measured at room temperature for 25^o^C in a two-electrode electrochemical full cell. The electrolyte was 3.0 M KOH aqueous solution.

1. **Calculations**

The mass-specific capacitance (*C/F g^-1^*) of the device can also be calculated using：

*C=Q / (m×△V)= ∫Idt / (m×△V)= I×t_discharge_ / (m×△V)*  (1)

where *m* is the mass of the activated materials, *I* is the discharge current, *t_discharge_* is

discharge time, and *△V* is the potential drop during discharge.

The area-specific capacitance (*C/mF cm^-2^*) of the device can also be calculated using：

*C=Q / (A×△V)= ∫Idt / ( A×△V)= I×t_discharge_ / ( A×△V)*  (2)

where *A* is the surface area of the device, *I* is the discharge current, *t_discharge_* is

discharge time, and *△V* is the potential drop during discharge.

The kinetic of capacitive contribution can be obtained through calculating the CV curves at different scan rates. The relationship between current (*i*) and scan rate (*v*) can be written as:

*i=av^b^* (5)

*log(i)=b×log(v)+log(a)* (6)

where *a* and *b* are constant can be obtained from *log(v)* versus *log(i)* plots. When b=0.5 represents an ideal diffusion-controlled process and when b=1.0 indicates a surface capacitive-controlled process. The capacitive contributions at different scan rates can be calculated by the equations described as below:

*i=k_1_v+k_2_v^1/2^*  (7)

*i/v^1/2^=k_1_v^1/2^+k_2_*  (8)

where *i* is the current density at a voltage (V), *v* is the scan rate (mV s^-1^), *k_1_* and *k_2_* can be obtained from the slope and intercept, respectively. Where *k_1_v* can be attributed to the current from surface capacitance contribution, while *k_2_v^1/2^* is indexed to the diffusion process.

1. **SEM images**


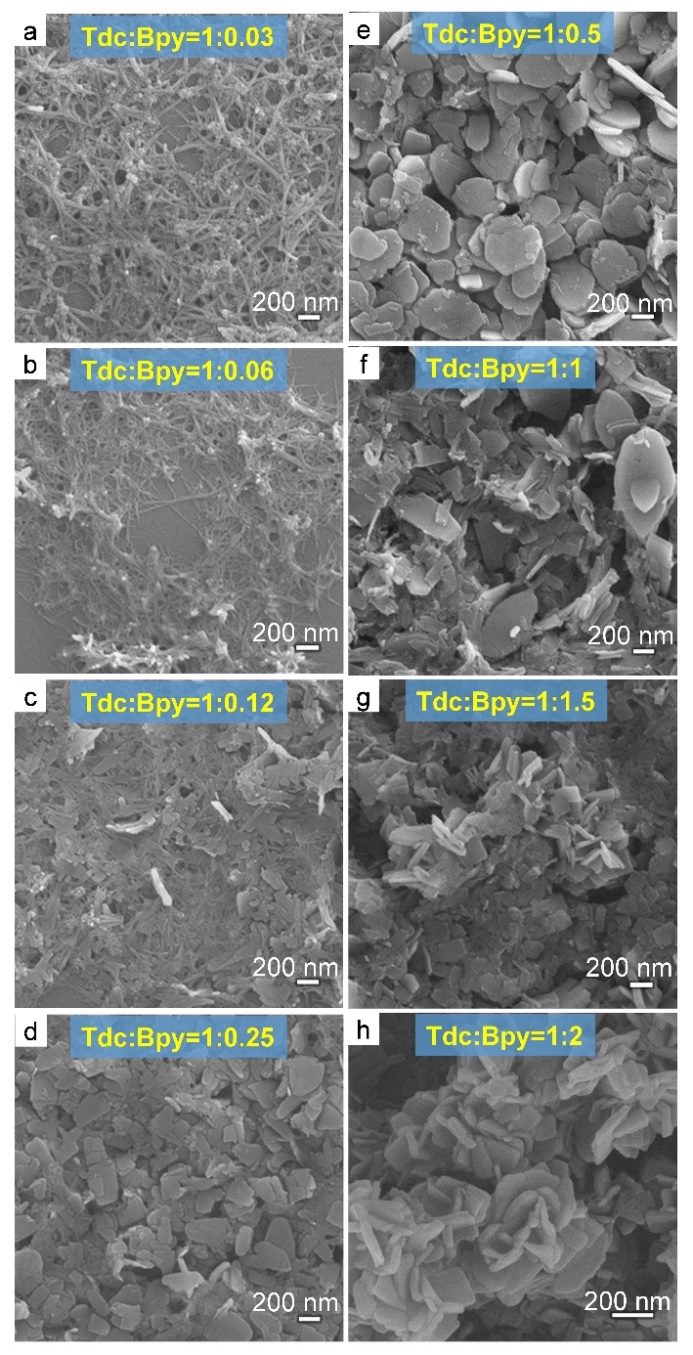


**Figure S1.** SEM images of the samples: a) M1, b) M2, c) M3, d) M4, e) M5, f) M6, g) M7, and h) M8.

1. **TEM images**


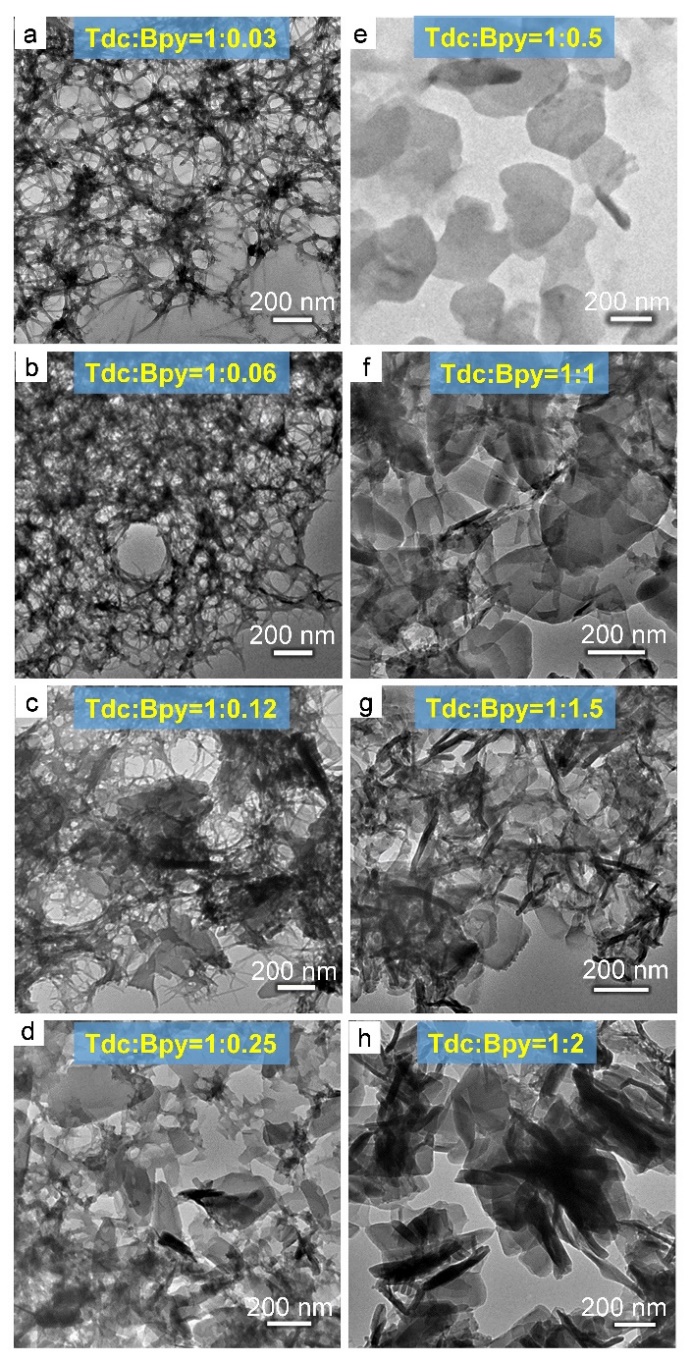


**Figure S2.** TEM images of the samples: a) M1, b) M2, c) M3, d) M4, e) M5, f) M6, g) M7, and h) M8.

1. **HRTEM images of M5**

**
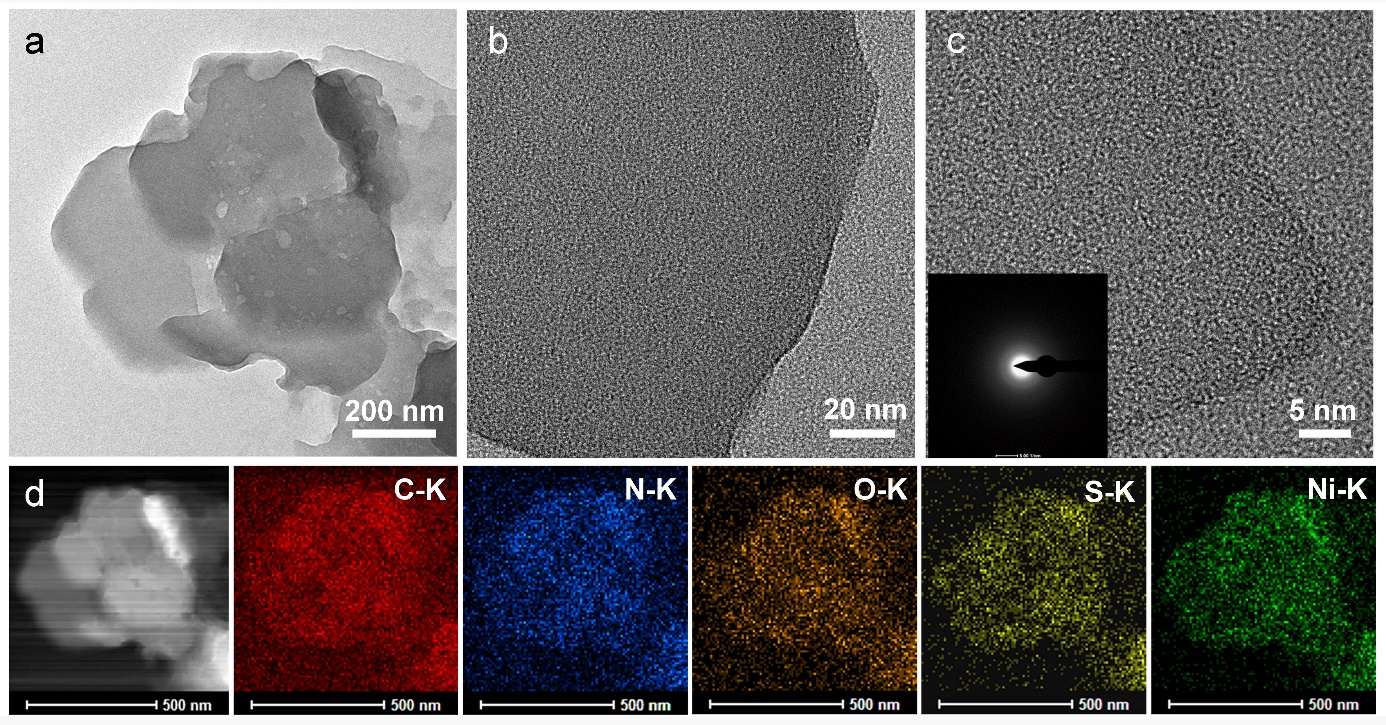
**

**Figure S3.** a) TEM image, b,c) HRTEM images (inset of (c): SAED pattern), and d) HAADF-STEM image and the corresponding elemental mappings of C-K, N-K, O-K, S-K, and Ni-K of M5.

1. **The stability of M5 in water and alcohol**

**
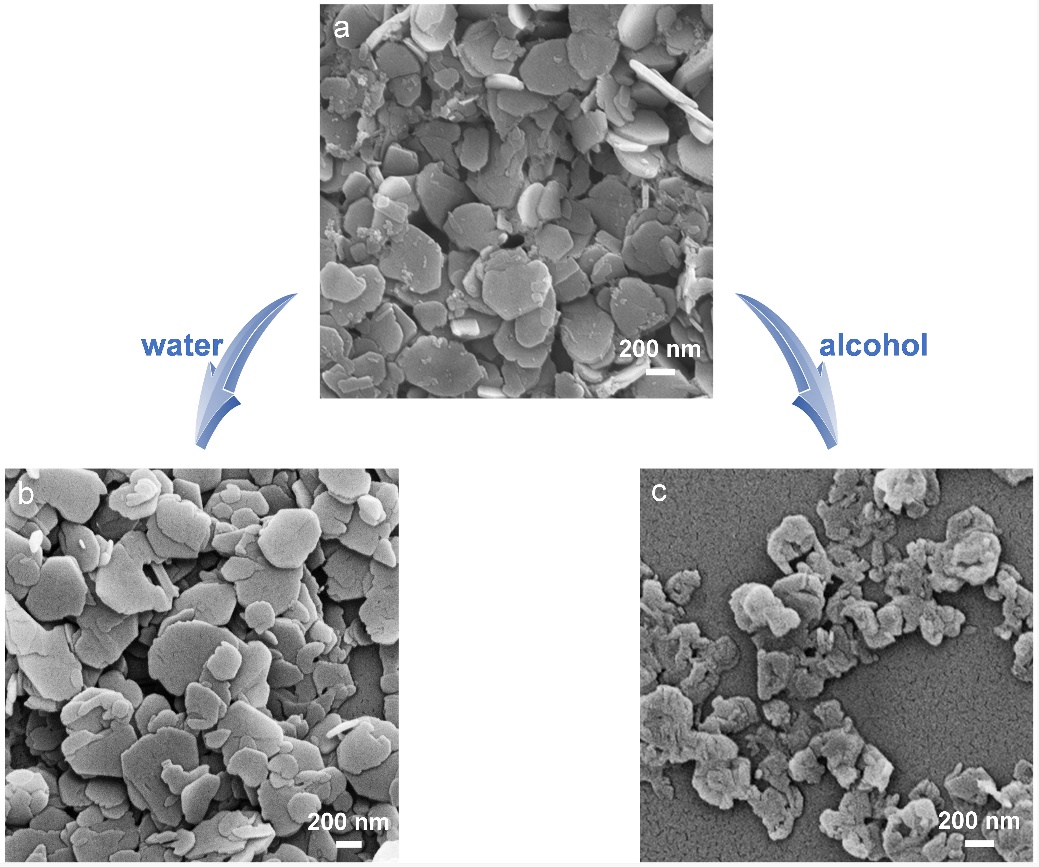
**

**Figure S4.** SEM images: a) M5, b) M5 after immersion in water for 7 days, and c) M5 after immersion in alcohol for 7 days.

1. **IR patterns**


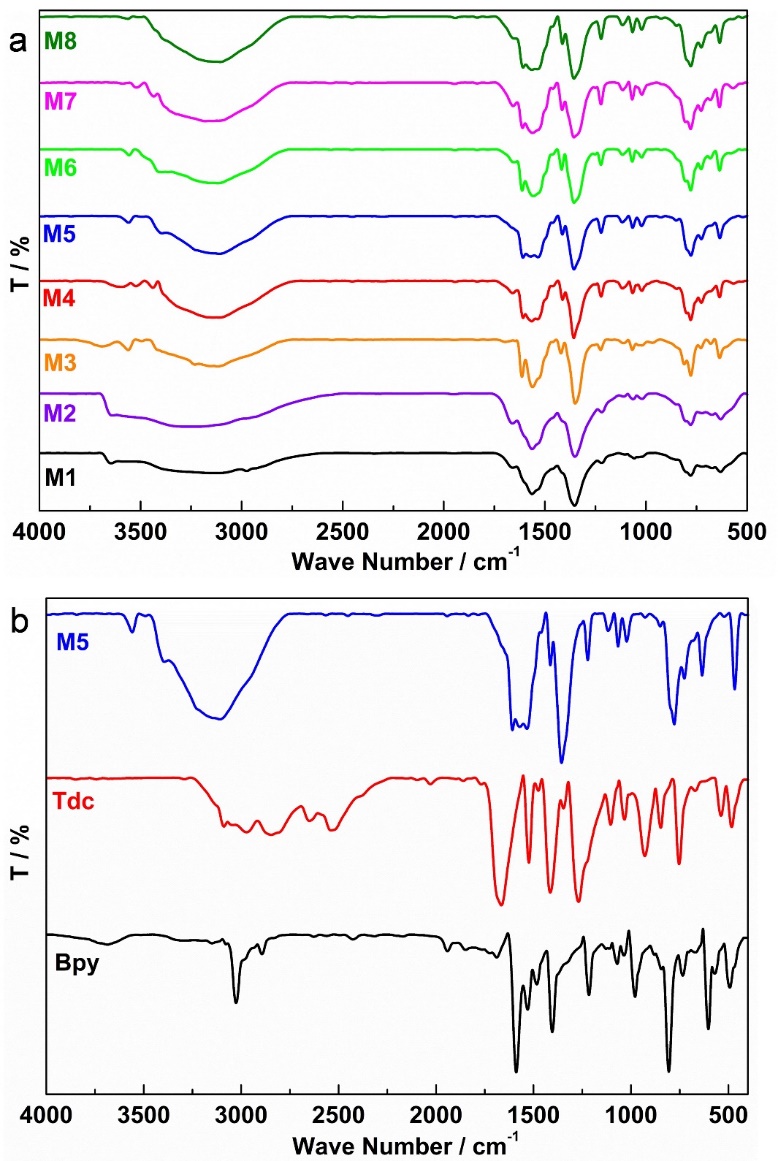


**Figure S5.** IR patterns of M1-M8, Tdc, and Bpy.

1. **XRD patterns**





**Figure S6.** XRD patterns of M1-M8.

1. **Structure of [Ni(Tdc)(Bpy)]_n_ MOF**


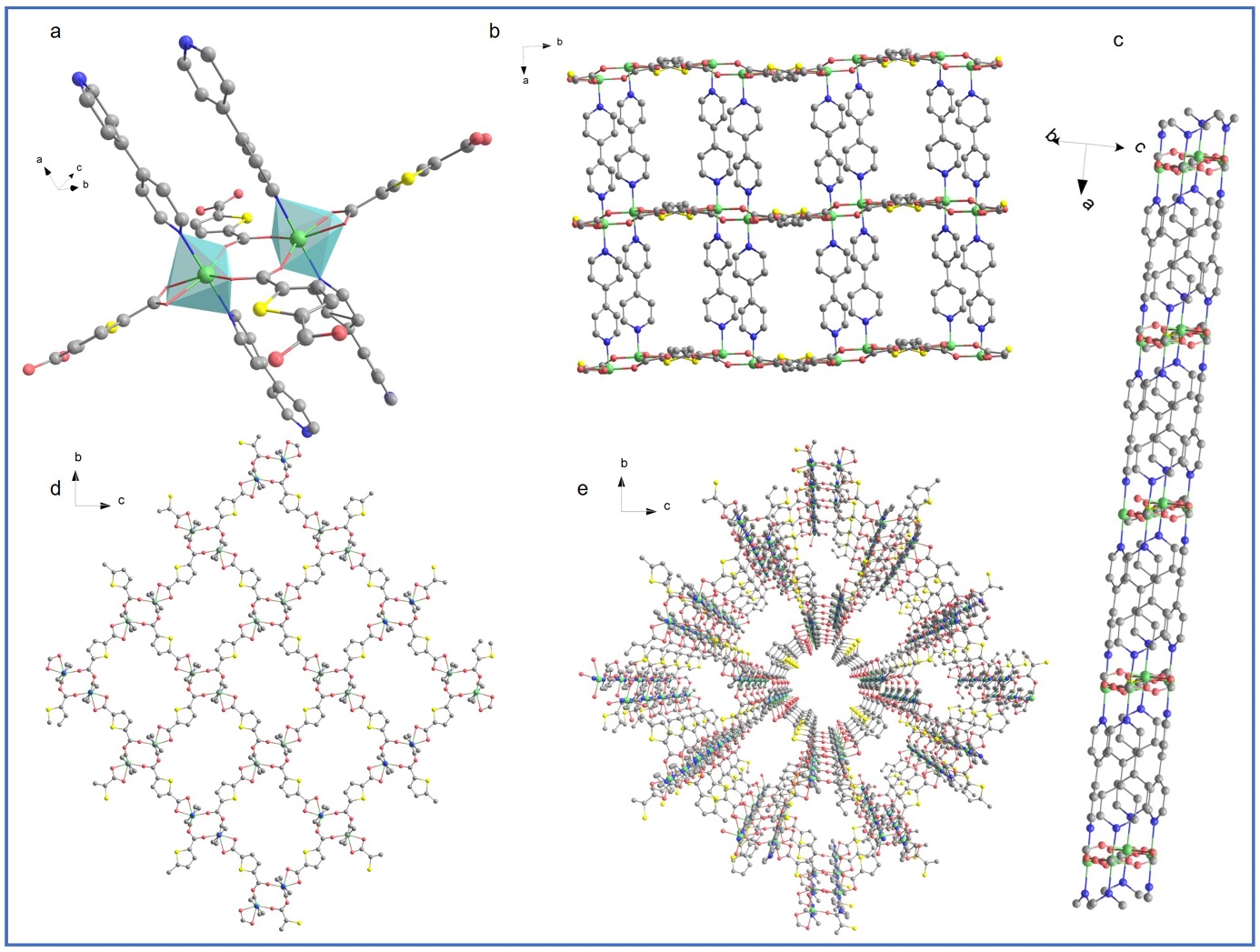


**Figure S7.** a) the coordination environment of nickel center, b) the accumulation viewed along the c direction, c) the accumulation viewed along the Bpy direction, d,e) the accumulation viewed along the a direction.


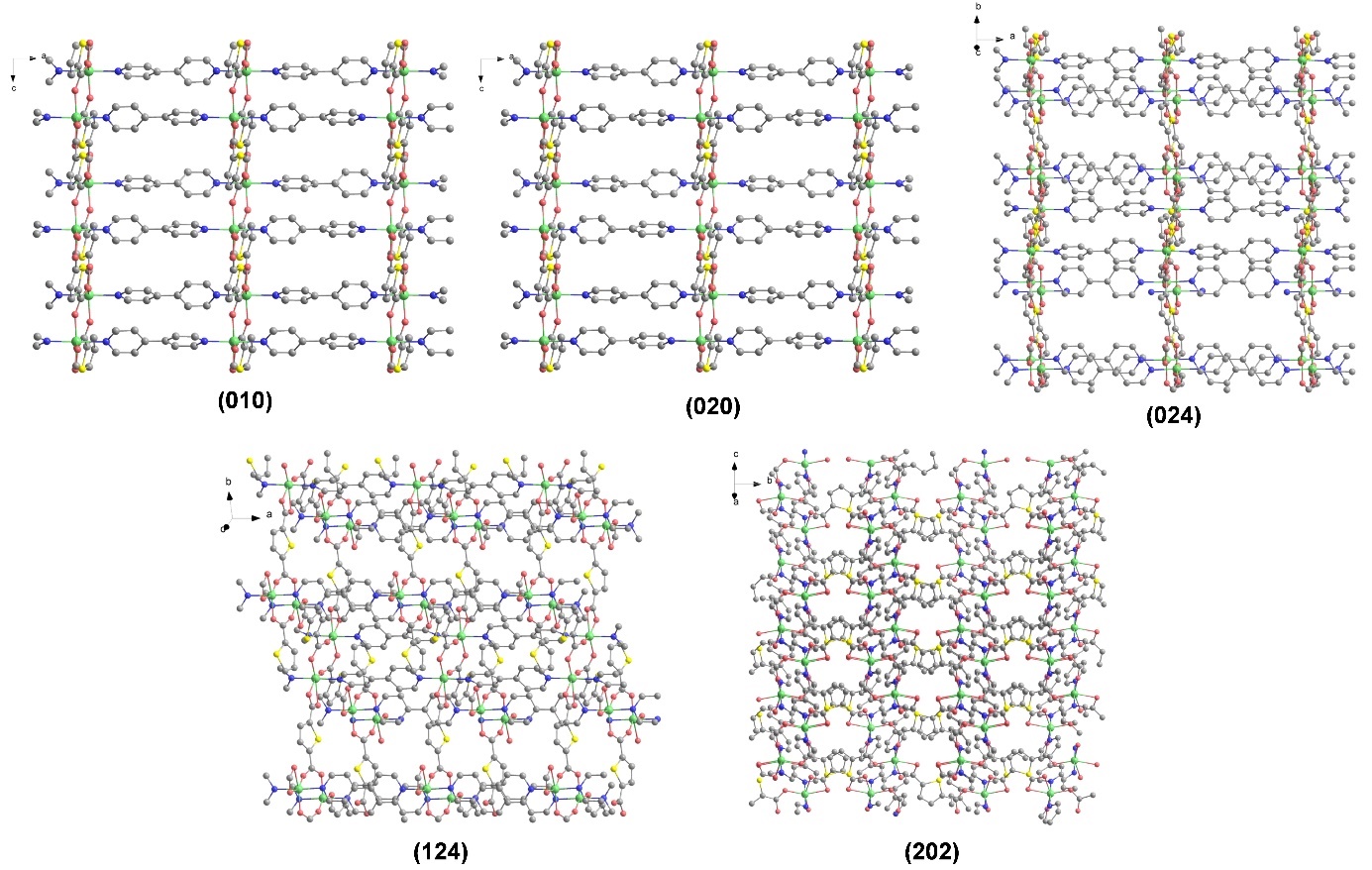


**Figure S8.** The accumulations viewed along the (010), (020), (024), (124) and (202) direction.

1. **XPS spectra of the M1**


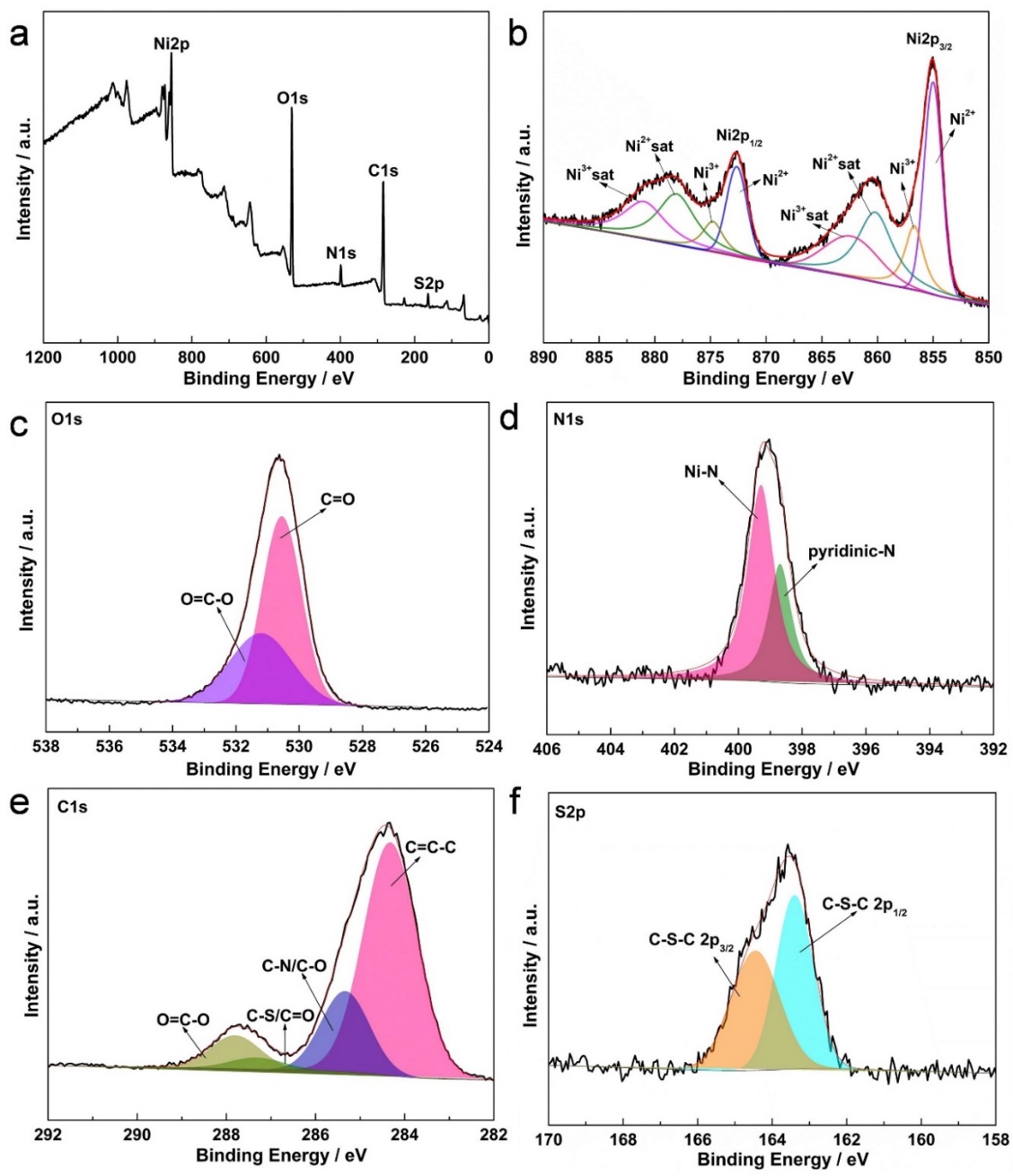


**Figure S9.** XPS spectra of the M1. a) Survey, and high resolution b) Ni 2p, c) O 1s, d) N 1s, e) C 1s and f) S 2p XPS spectra.

1. **XPS spectra of the M2**


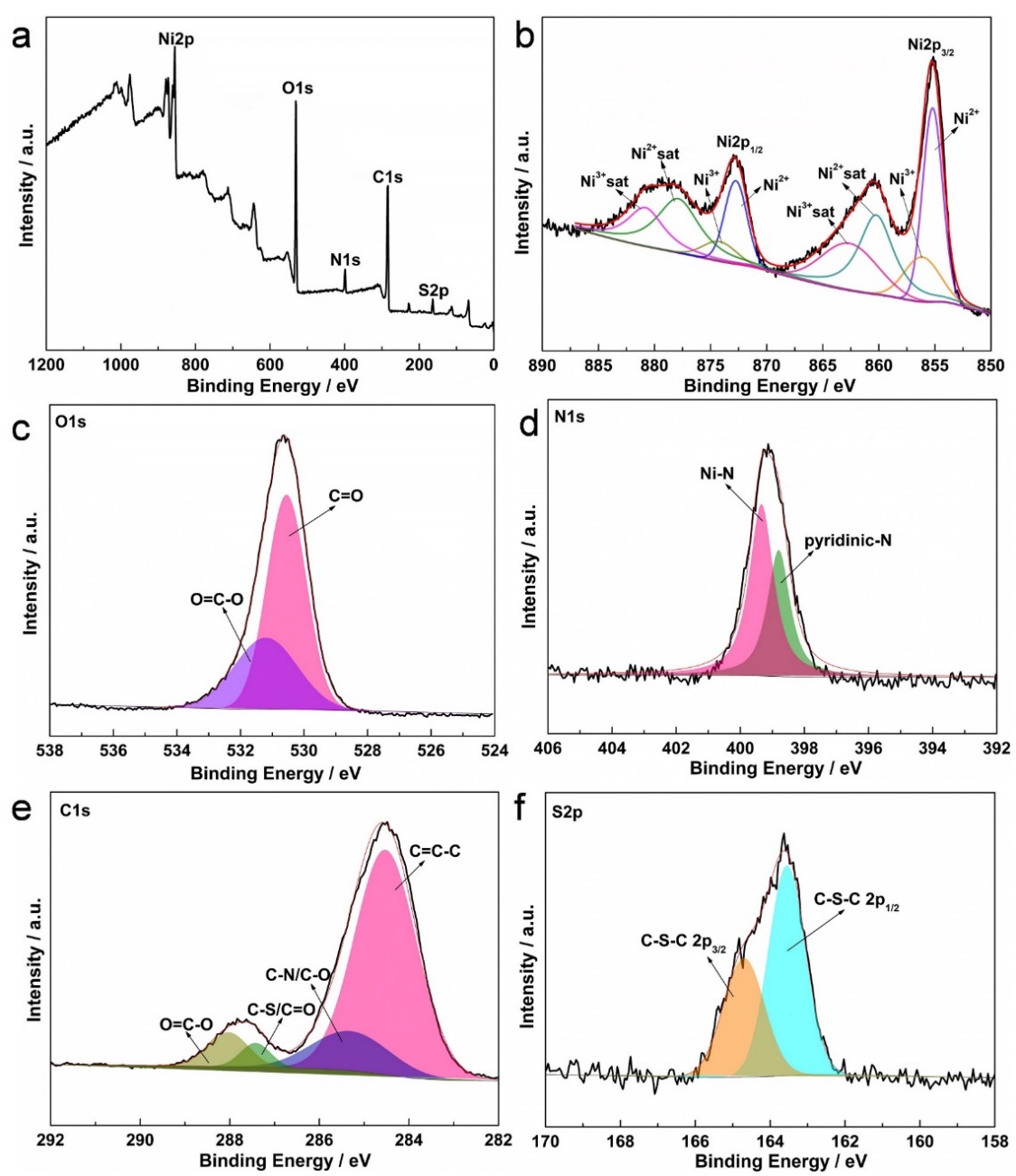


**Figure S10.** XPS spectra of the M2. a) Survey, and high resolution b) Ni 2p, c) O 1s, d) N 1s, e) C 1s and f) S 2p XPS spectra.

1. **XPS spectra of the M3**


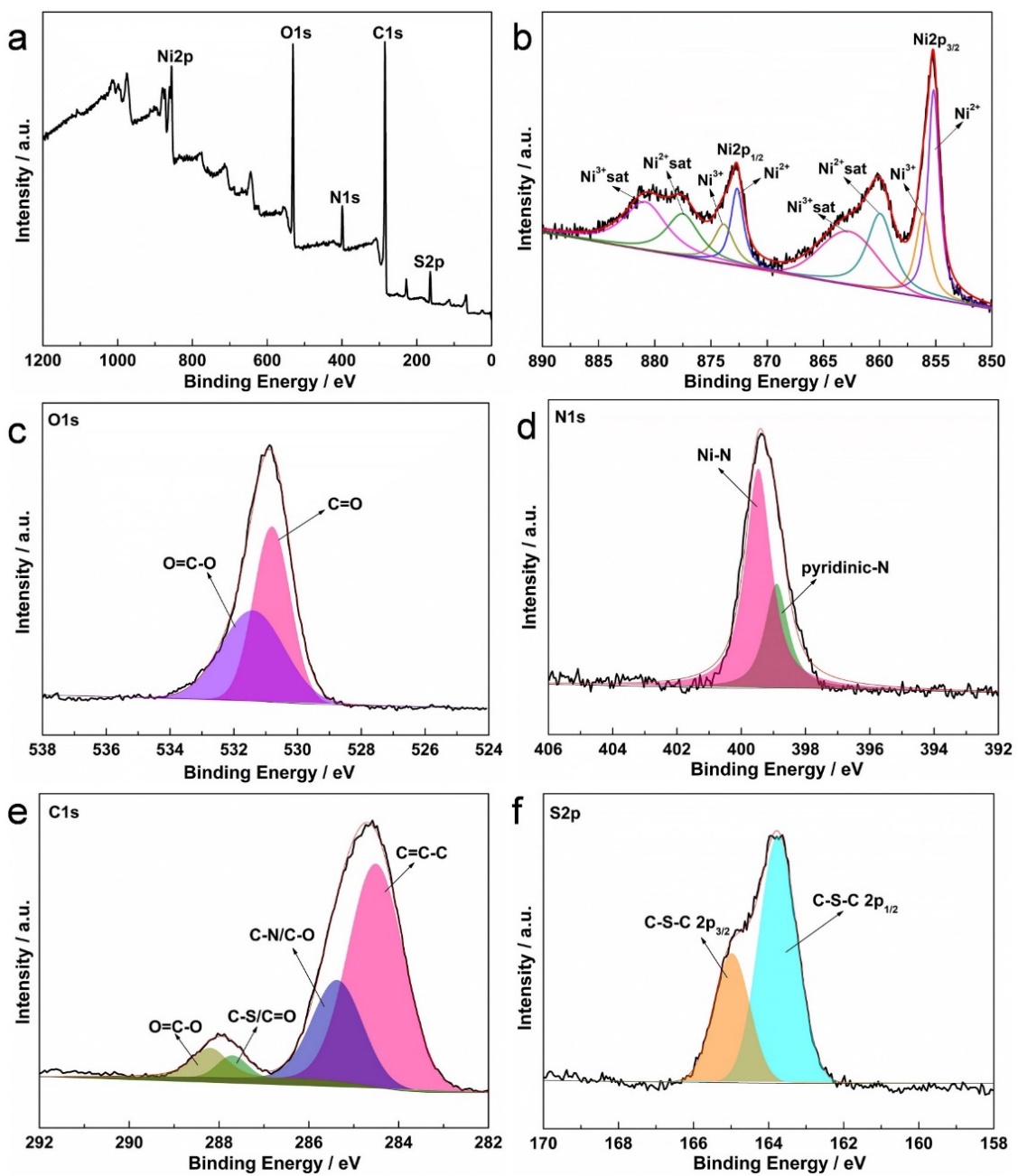


**Figure S11.** XPS spectra of the M3. a) Survey, and high resolution b) Ni 2p, c) O 1s, d) N 1s, e) C 1s and f) S 2p XPS spectra.

1. **XPS spectra of the M4**


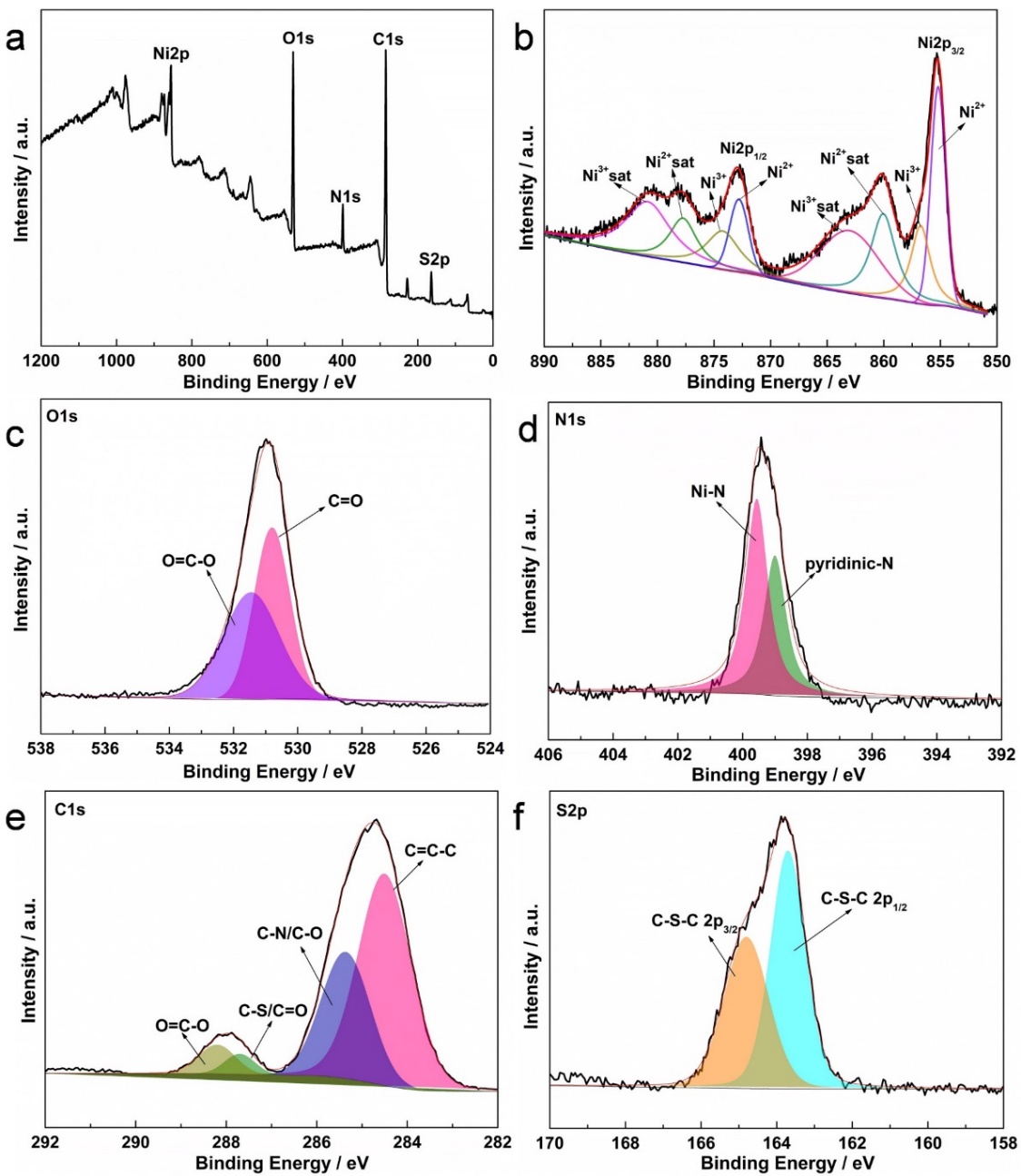


**Figure S12.** XPS spectra of the M4. a) Survey, and high resolution b) Ni 2p, c) O 1s, d) N 1s, e) C 1s and f) S 2p XPS spectra.

1. **XPS spectra of the M6**


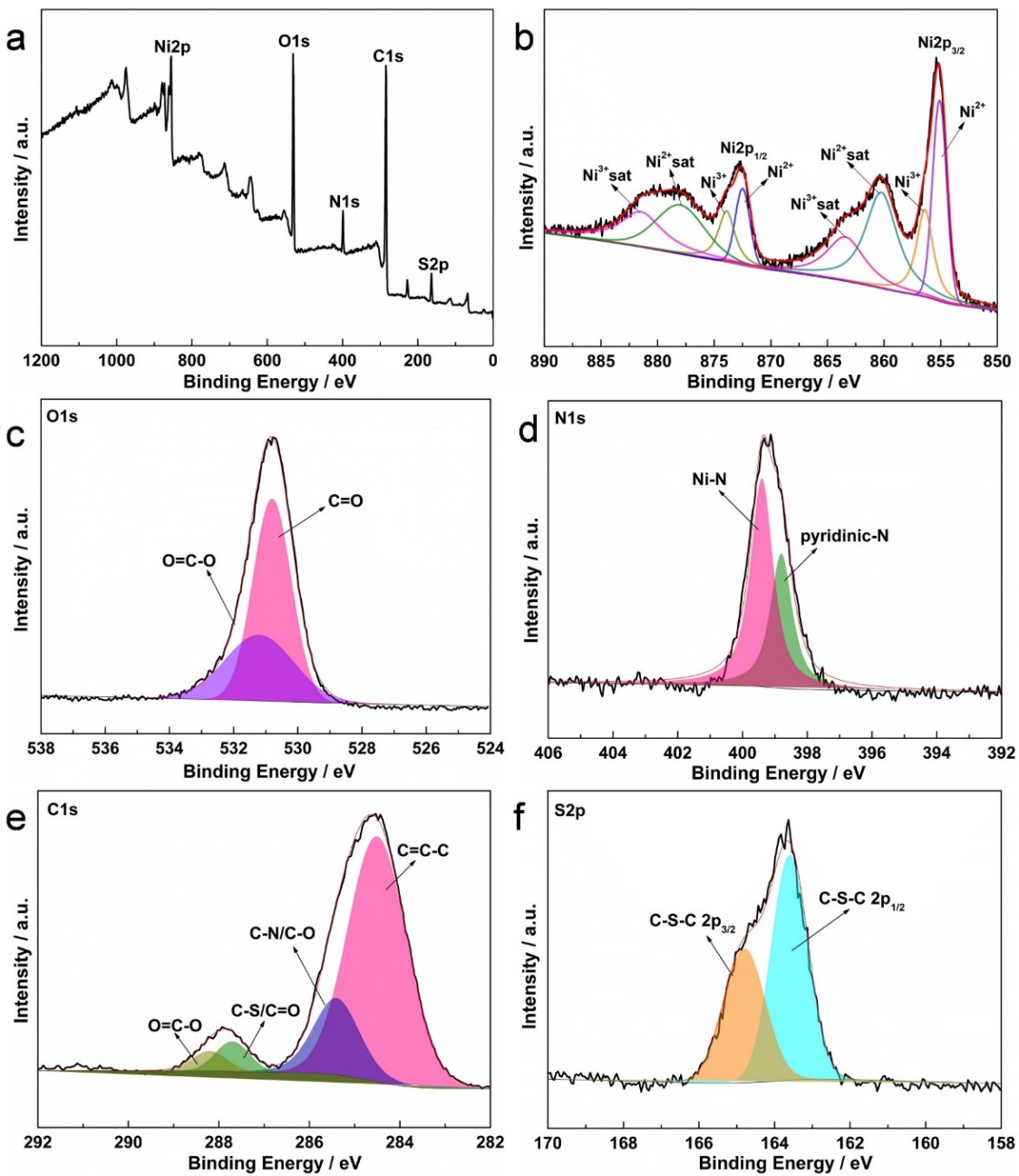


**Figure S13.** XPS spectra of the M6. a) Survey, and high resolution b) Ni 2p, c) O 1s, d) N 1s, e) C 1s and f) S 2p XPS spectra.

1. **XPS spectra of the M7**


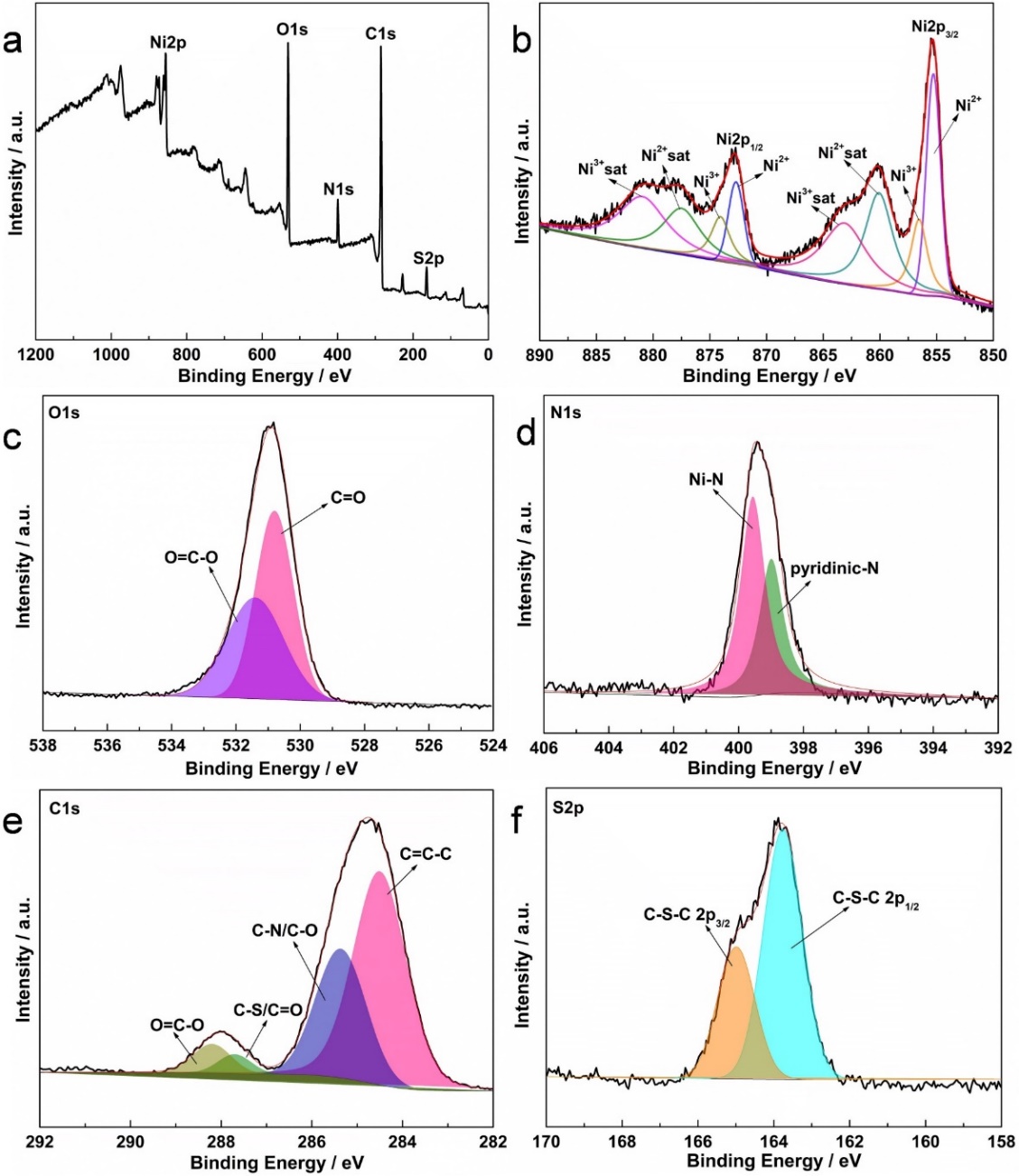


**Figure S14.** XPS spectra of the M7. a) Survey, and high resolution b) Ni 2p, c) O 1s, d) N 1s, e) C 1s and f) S 2p XPS spectra.

1. **XPS spectra of the M8**


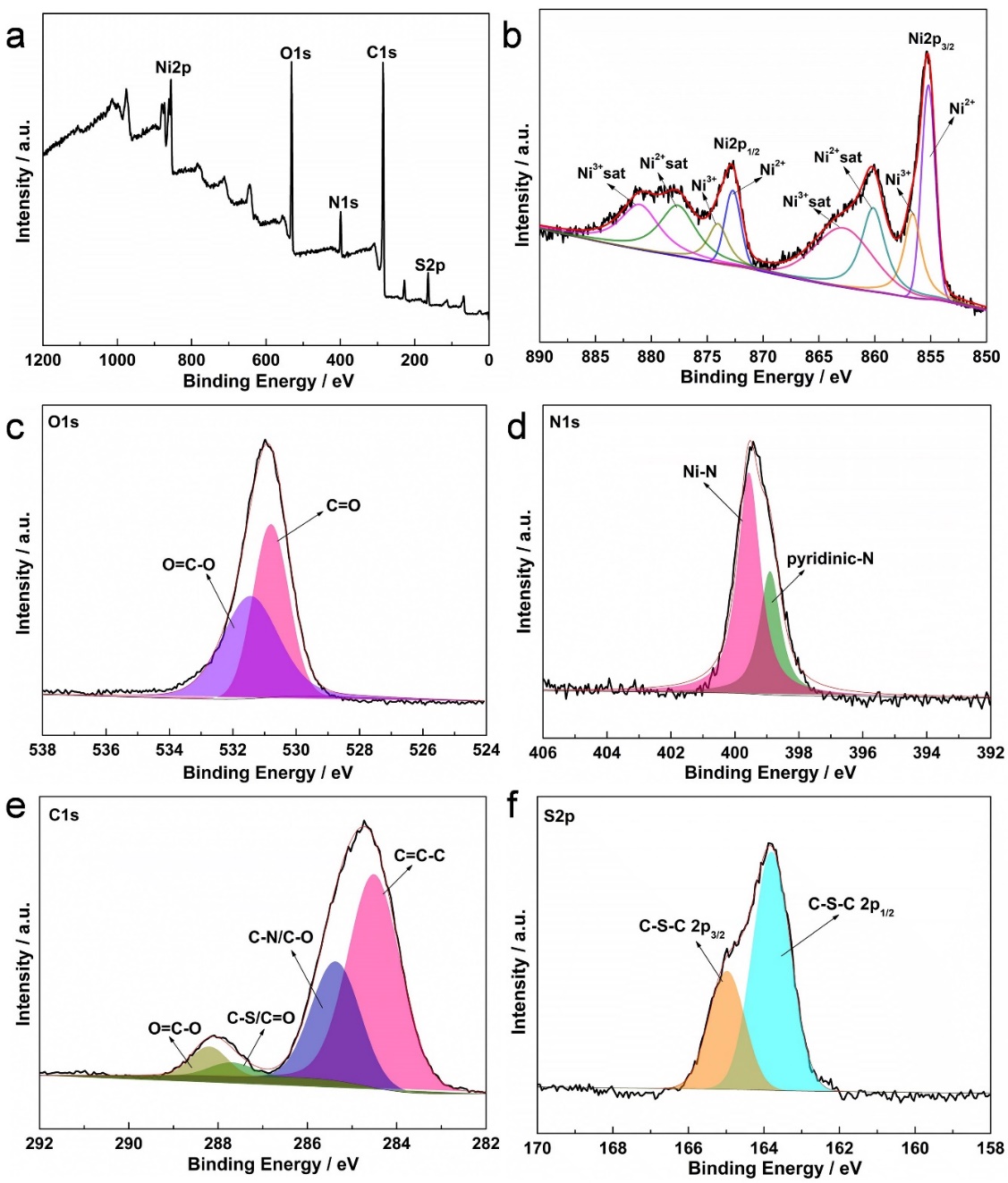


**Figure S15.** XPS spectra of the M8. a) Survey, and high resolution b) Ni 2p, c) O 1s, d) N 1s, e) C 1s and f) S 2p XPS spectra.

1. **BET**

**
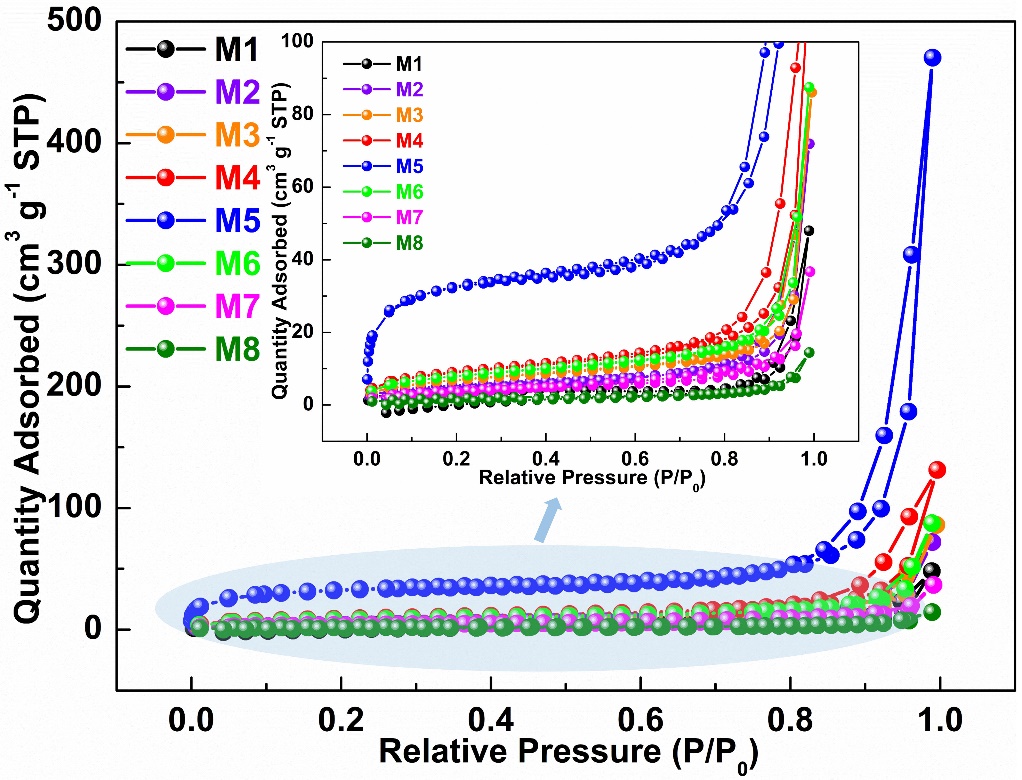
**

**Figure S16.** N_2_ adsorption-desorption isotherms of M1-M8.

1. **Table S2. BET surface area**

| **Sample** | **BET surface area (m^2^ g^-1^)** |
| --- | --- |
| **M1** | **13.07** |
| **M2** | **19.33** |
| **M3** | **24.65** |
| **M4** | **31.55** |
| **M5** | **105.27** |
| **M6** | **28.29** |
| **M7** | **14.22** |
| **M8** | **6.47** |

1. **Pore size distribution**

**
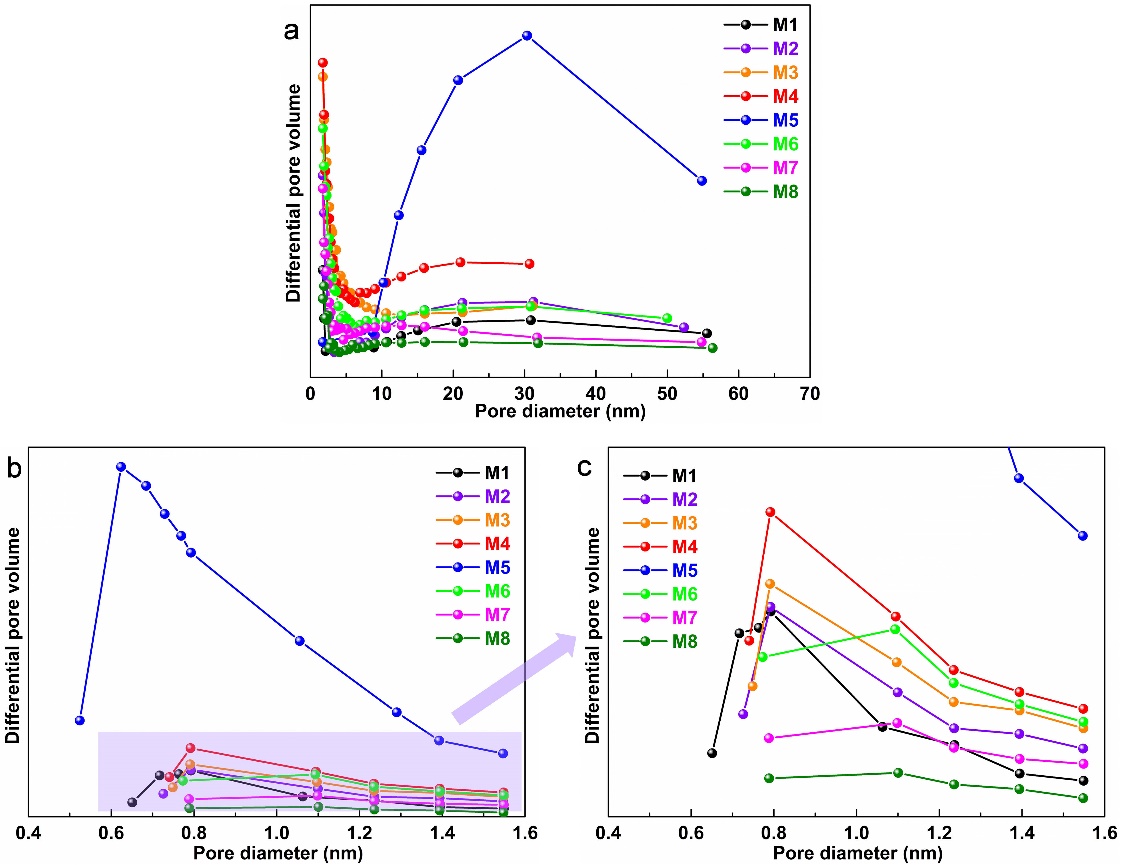
**

**Figure S17.** The pore size distribution of M1-M8 for the BJH adsorption branch (a) and HK method (b,c).

1. **SEM images of M1, M5, and M8 electrode**

**
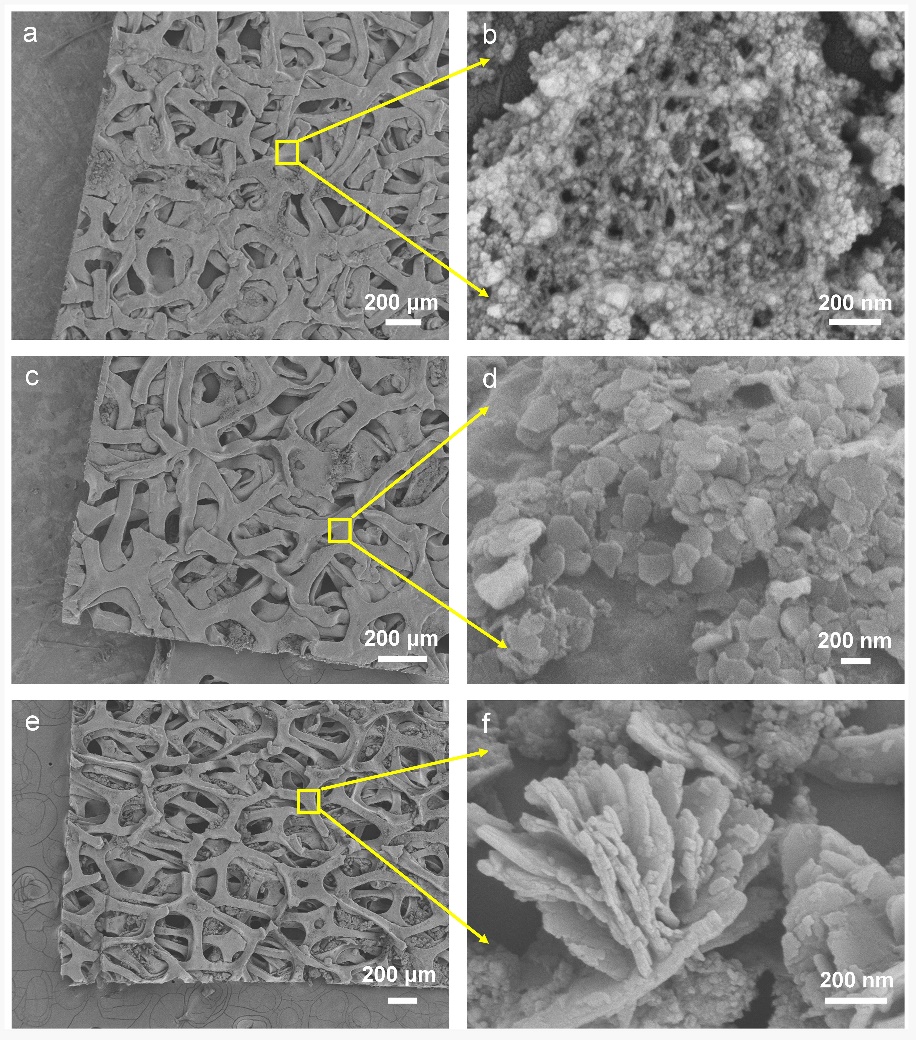
**

**Figure S18.** SEM images of M1 (a,b), M5 (c,d), and M8 (e,f).

1. **The analysis of ion-diffusion and capacitive contributions of the M1**


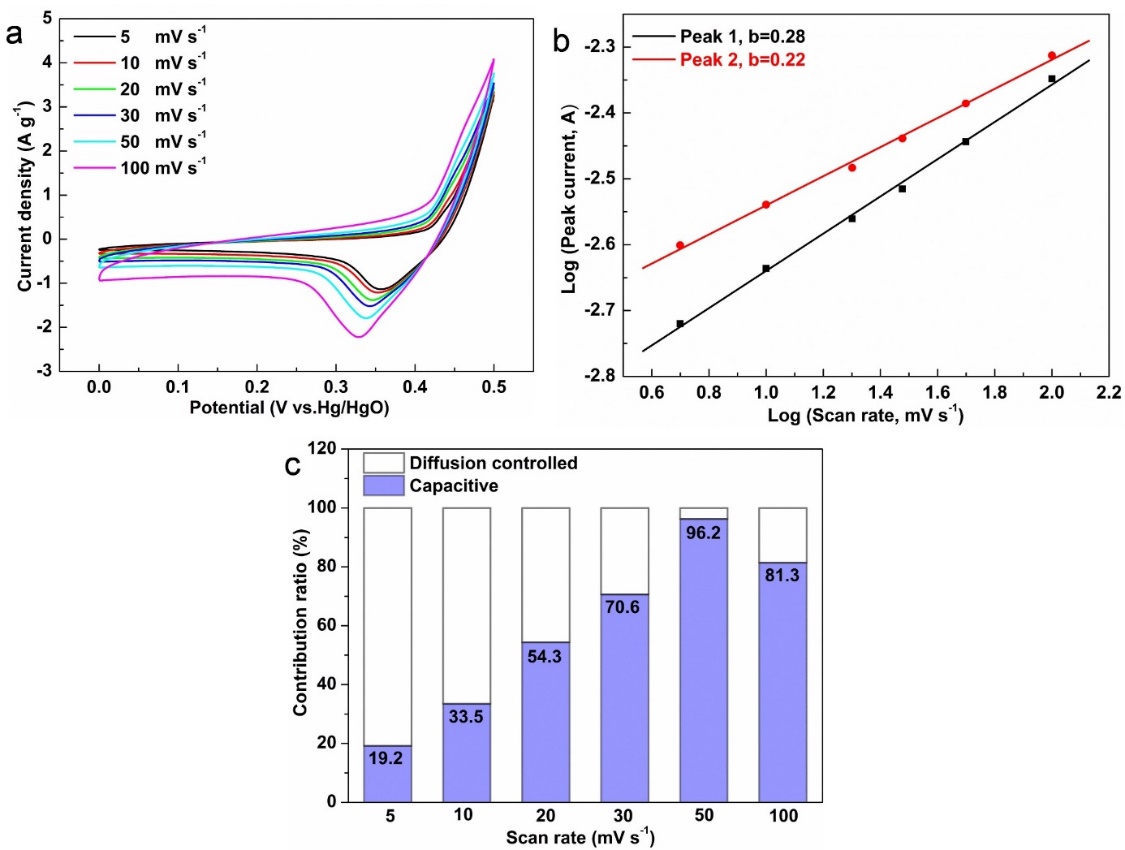


**Figure S19.** a) CV curves of the M1 at various scan rates of 5-100 mV s^-1^ in a three-electrode cell. b) Log*(i)* versus log*(v)* plots of the M1 at specific peak currents. c) Bar chart showing the percent of pseudocapacitive contribution of the M1 at different scan rates.

1. **Pseudocapacitive contribution shadow diagram of M1 in CV curves**


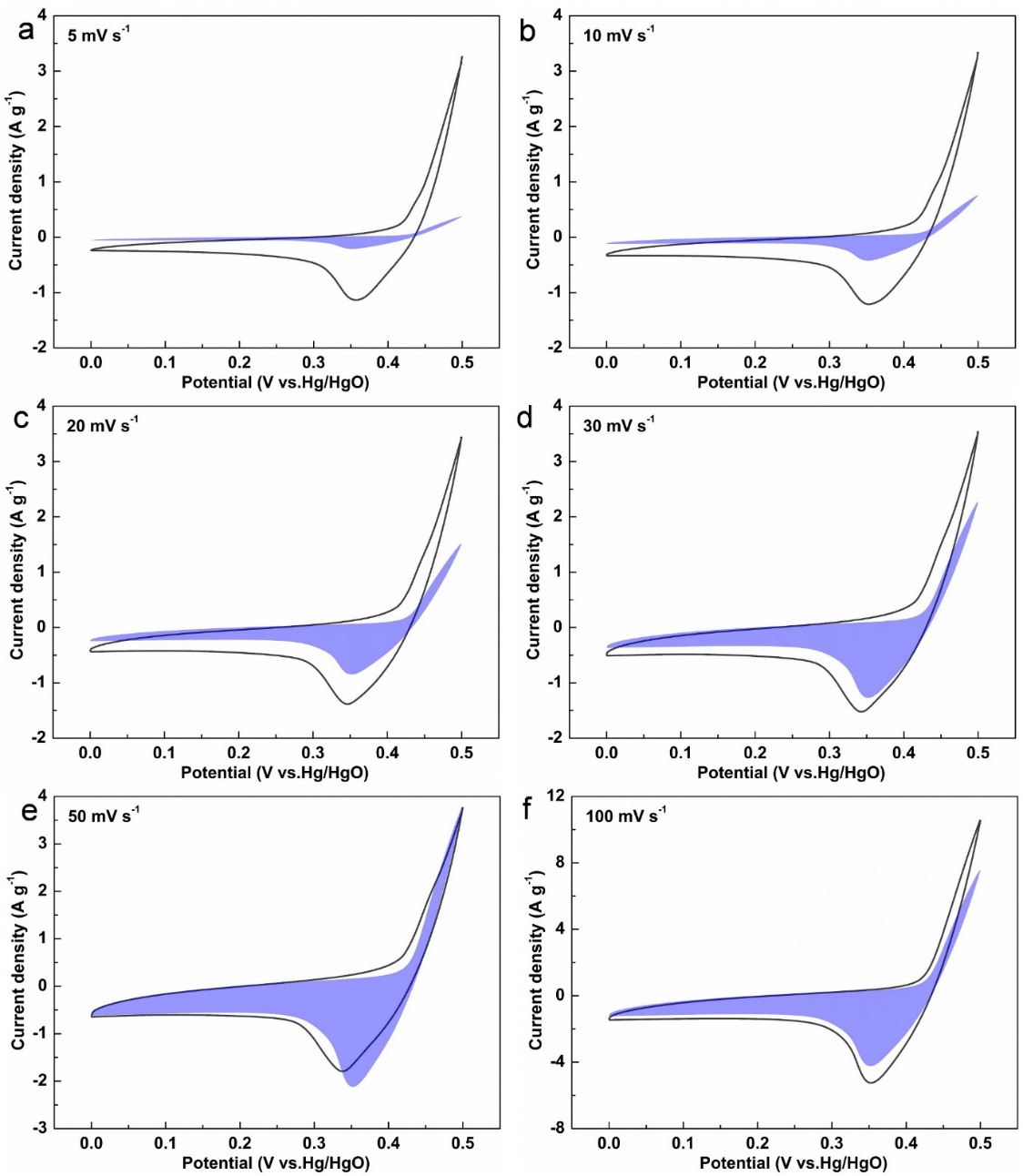


**Figure S20.** CV curve with the pseudocapacitive fraction shown by the shaded area of M1 at various scan rates in a three-electrode cell. a) 5 mV s^-1^. b) 10 mV s^-1^. c) 20 mV s^-1^. d) 30 mV s^-1^. e) 50 mV s^-1^. f) 100 mV s^-1^.

1. **The analysis of ion-diffusion and capacitive contributions of the M2**


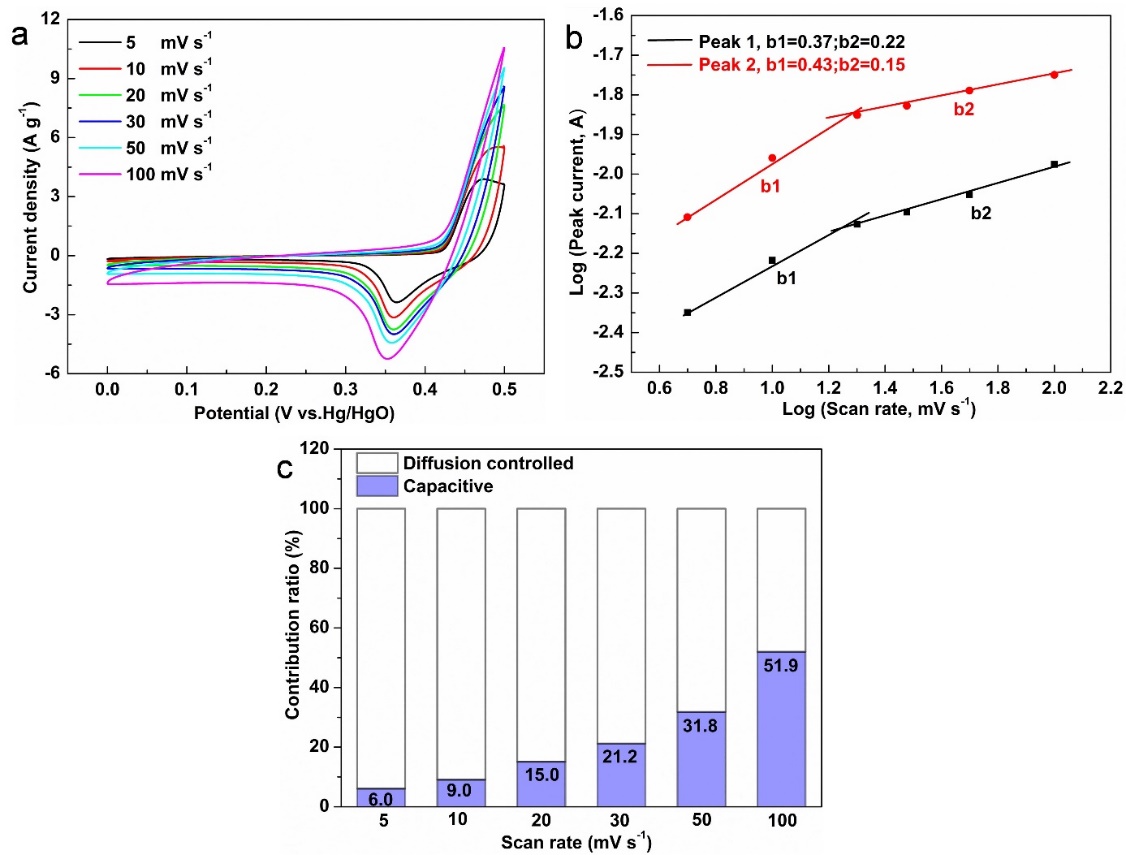


**Figure S21.** a) CV curves of the M2 at various scan rates of 5-100 mV s^-1^ in a three-electrode cell. b) Log*(i)* versus log*(v)* plots of the M2 at specific peak currents. c) Bar chart showing the percent of pseudocapacitive contribution of the M2 at different scan rates.

1. **Pseudocapacitive contribution shadow diagram of M2 in CV curves**


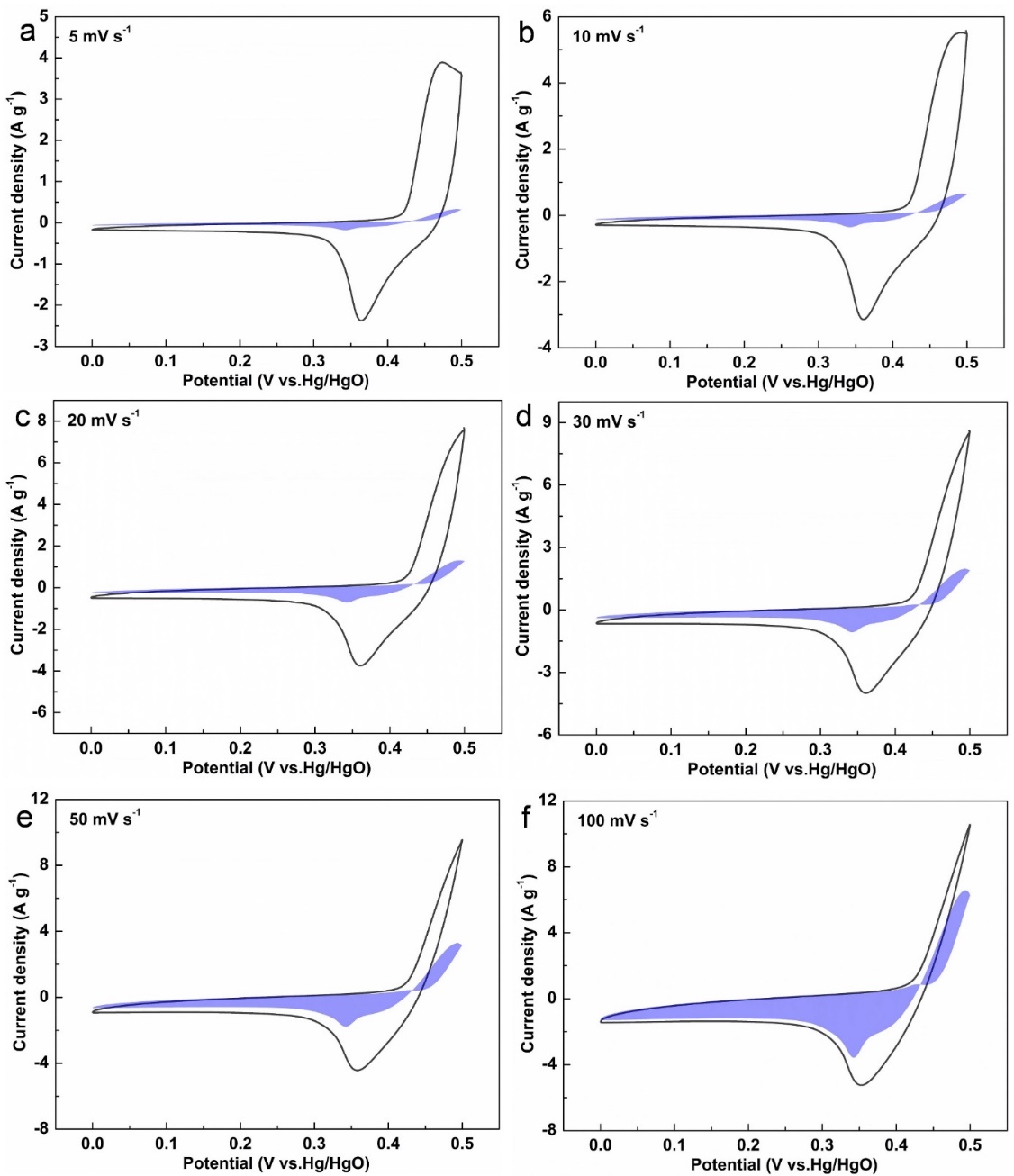


**Figure S22.** CV curve with the pseudocapacitive fraction shown by the shaded area of M2 at various scan rates in a three-electrode cell. a) 5 mV s^-1^. b) 10 mV s^-1^. c) 20 mV s^-1^. d) 30 mV s^-1^. e) 50 mV s^-1^. f) 100 mV s^-1^.

1. **The analysis of ion-diffusion and capacitive contributions of the M3**


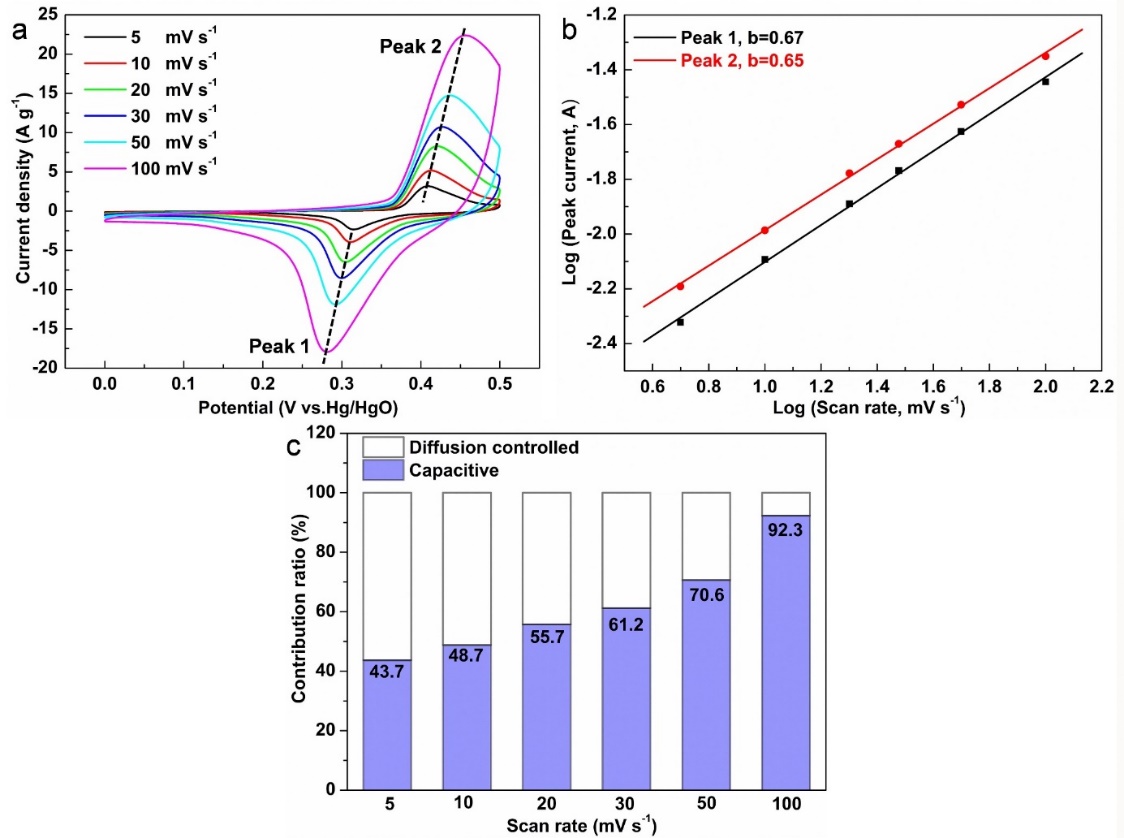


**Figure S23.** a) CV curves of the M3 at various scan rates of 5-100 mV s^-1^ in a three-electrode cell. b) Log*(i)* versus log*(v)* plots of the M3 at specific peak currents. c) Bar chart showing the percent of pseudocapacitive contribution of the M3 at different scan rates.

1. **Pseudocapacitive contribution shadow diagram of M3 in CV curves**


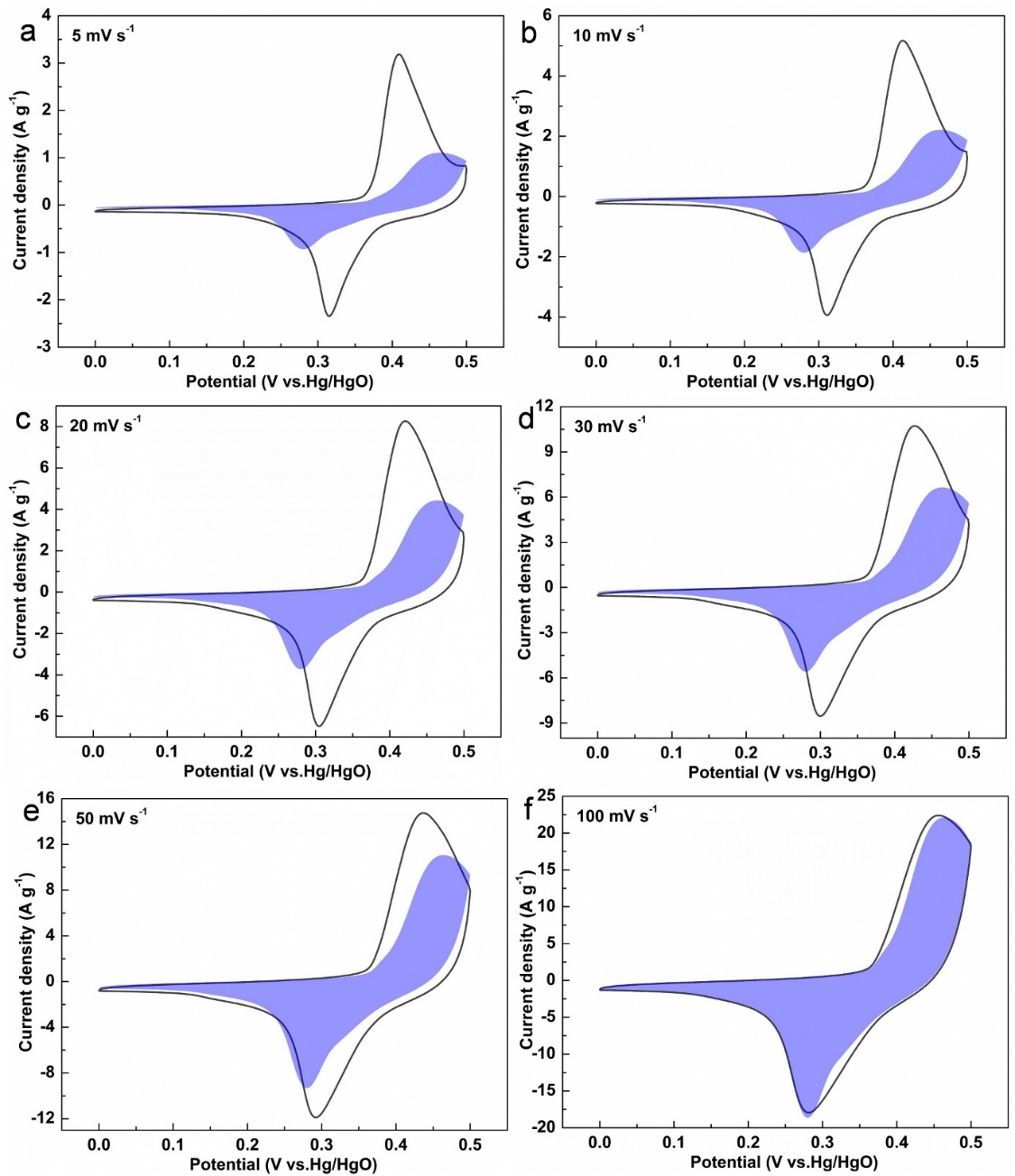


**Figure S24.** CV curve with the pseudocapacitive fraction shown by the shaded area of M3 at various scan rates in a three-electrode cell. a) 5 mV s^-1^. b) 10 mV s^-1^. c) 20 mV s^-1^. d) 30 mV s^-1^. e) 50 mV s^-1^. f) 100 mV s^-1^.

1. **The analysis of ion-diffusion and capacitive contributions of the M4**


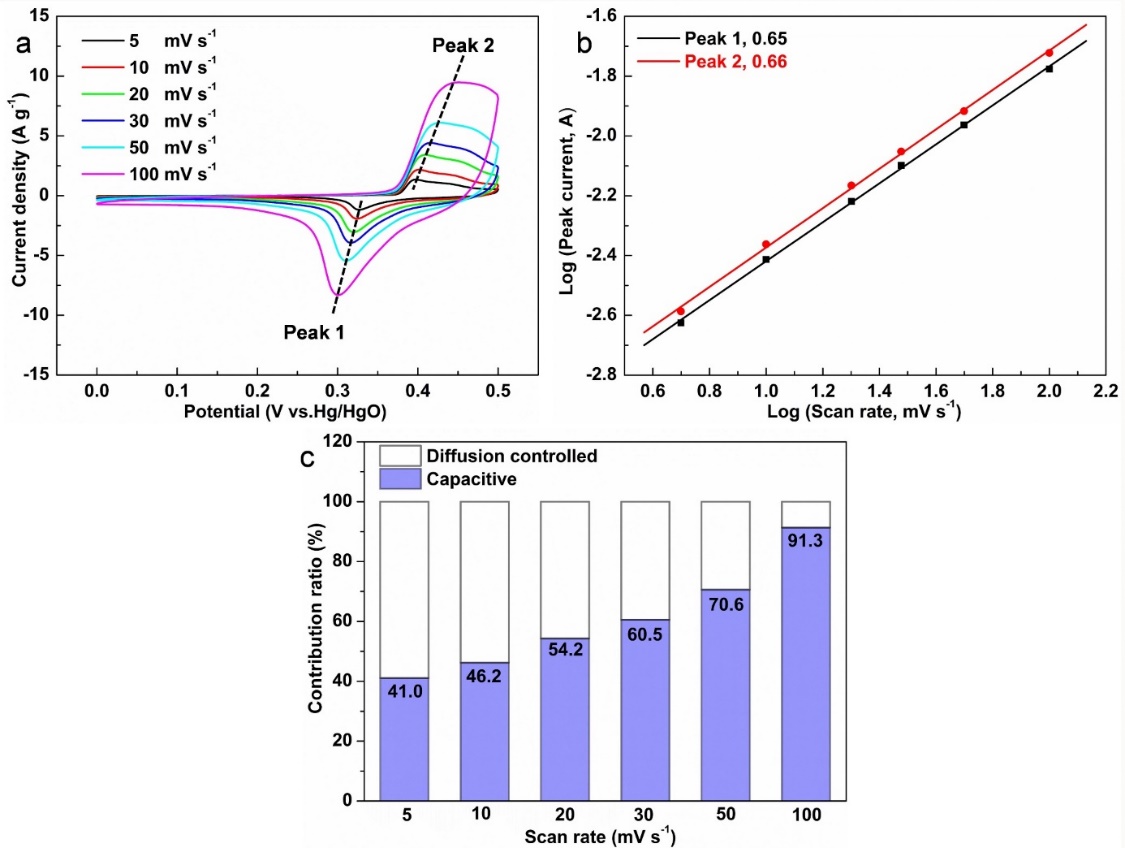


**Figure S25.** a) CV curves of the M4 at various scan rates of 5-100 mV s^-1^ in a three-electrode cell. b) Log*(i)* versus log*(v)* plots of the M4 at specific peak currents. c) Bar chart showing the percent of pseudocapacitive contribution of the M4 at different scan rates.

1. **Pseudocapacitive contribution shadow diagram of M4 in CV curves**


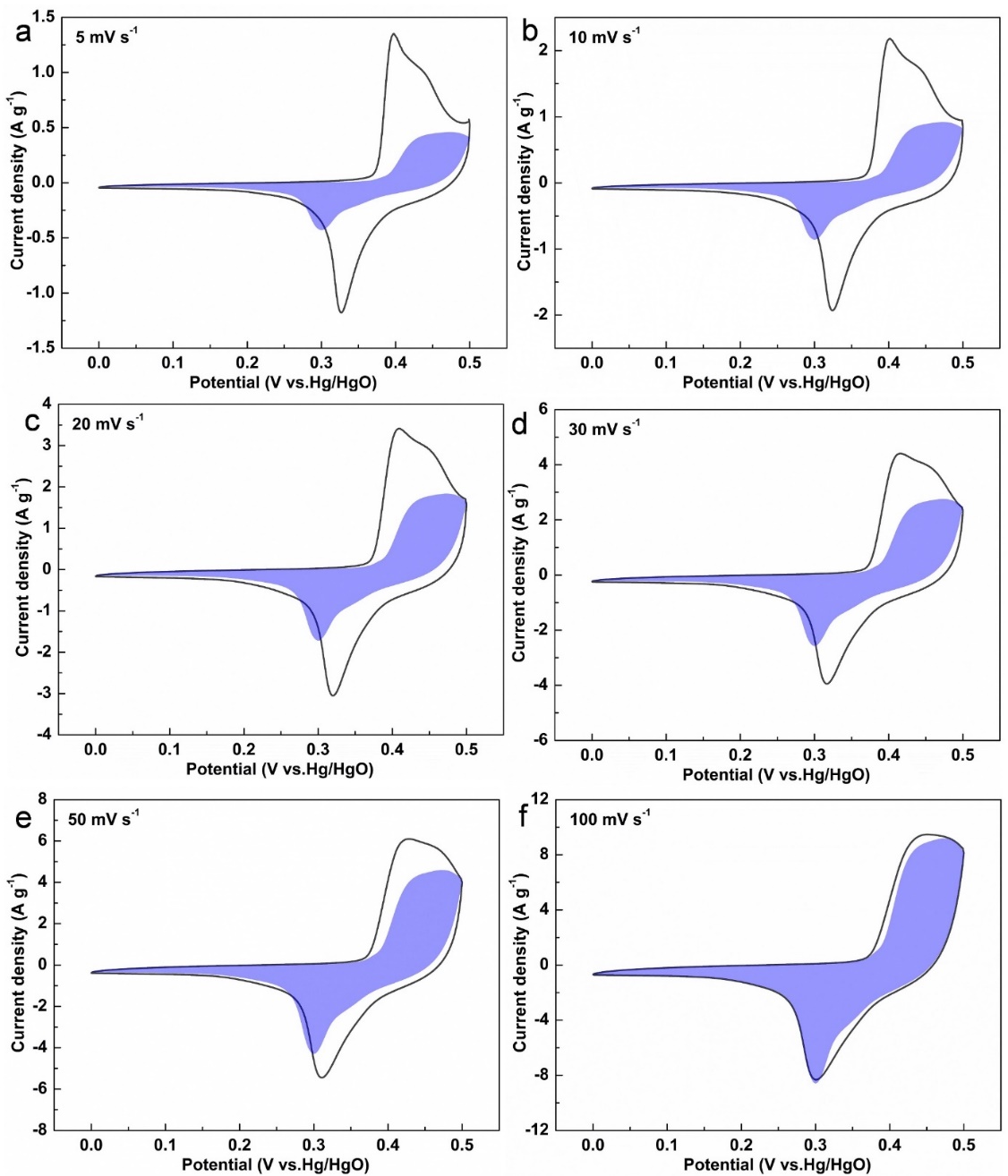


**Figure S26.** CV curve with the pseudocapacitive fraction shown by the shaded area of M4 at various scan rates in a three-electrode cell. a) 5 mV s^-1^. b) 10 mV s^-1^. c) 20 mV s^-1^. d) 30 mV s^-1^. e) 50 mV s^-1^. f) 100 mV s^-1^.

1. **Pseudocapacitive contribution shadow diagram of M5 in CV curves**


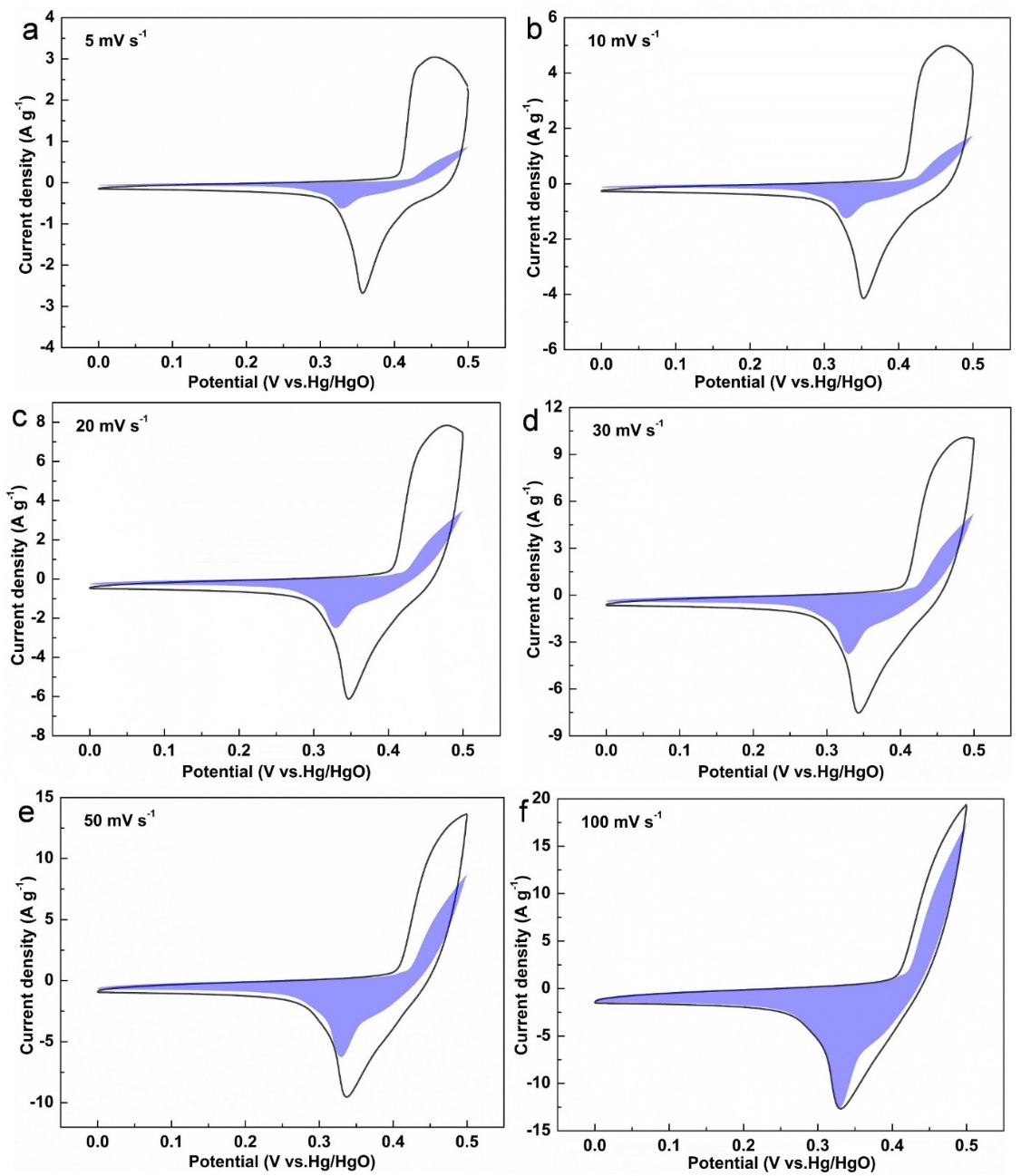


**Figure S27.** CV curve with the pseudocapacitive fraction shown by the shaded area of M5 at various scan rates in a three-electrode cell. a) 5 mV s^-1^. b) 10 mV s^-1^. c) 20 mV s^-1^. d) 30 mV s^-1^. e) 50 mV s^-1^. f) 100 mV s^-1^.

1. **The analysis of ion-diffusion and capacitive contributions of the M6**


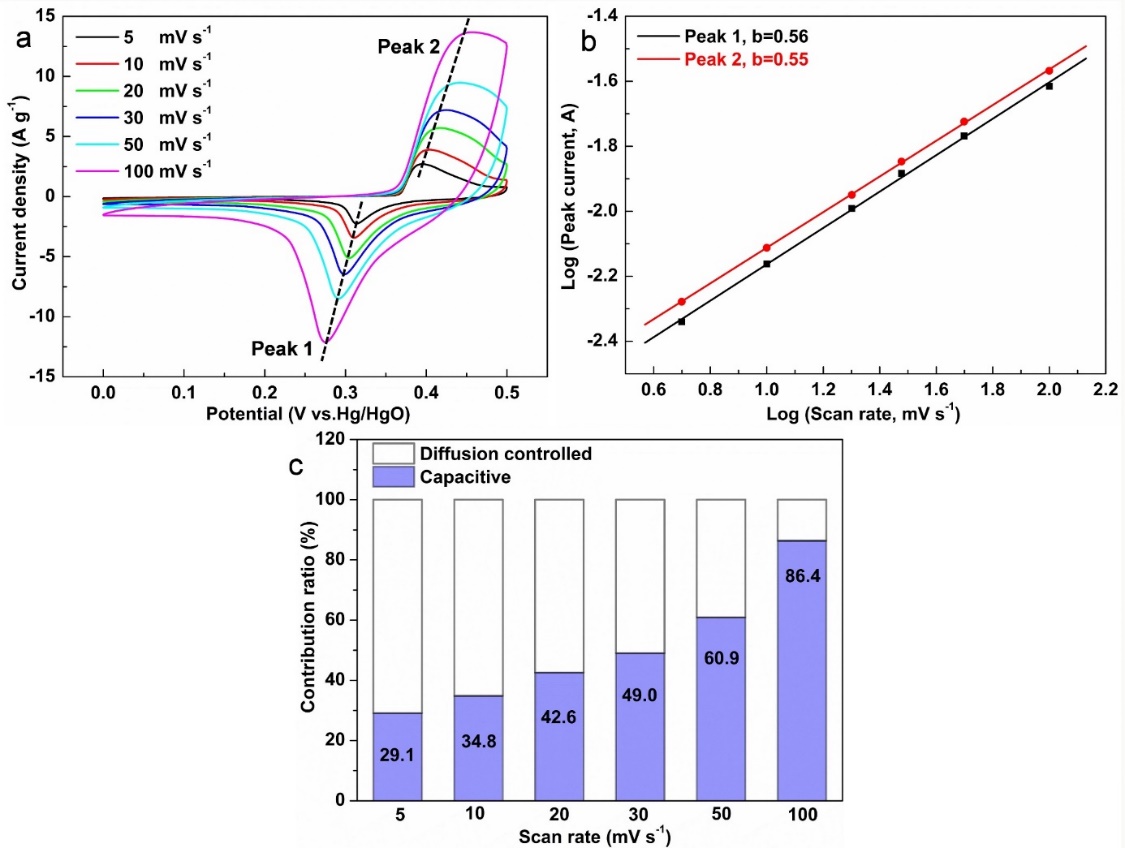


**Figure S28.** a) CV curves of the M6 at various scan rates of 5-100 mV s^-1^ in a three-electrode cell. b) Log*(i)* versus log*(v)* plots of the M6 at specific peak currents. c) Bar chart showing the percent of pseudocapacitive contribution of the M6 at different scan rates.

1. **Pseudocapacitive contribution shadow diagram of M6 in CV curves**


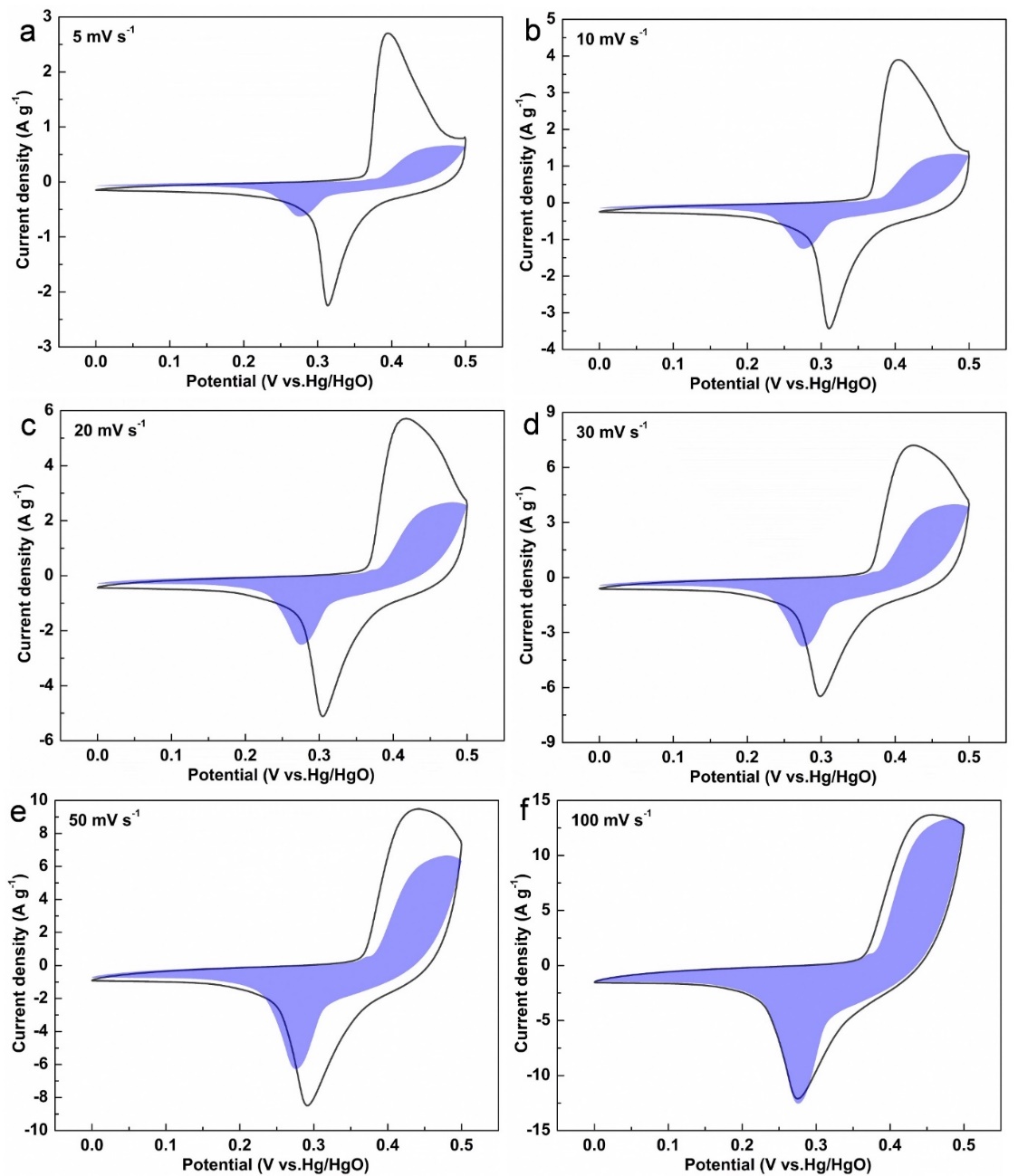


**Figure S29.** CV curve with the pseudocapacitive fraction shown by the shaded area of M6 at various scan rates in a three-electrode cell. a) 5 mV s^-1^. b) 10 mV s^-1^. c) 20 mV s^-1^. d) 30 mV s^-1^. e) 50 mV s^-1^. f) 100 mV s^-1^.

1. **The analysis of ion-diffusion and capacitive contributions of the M7**


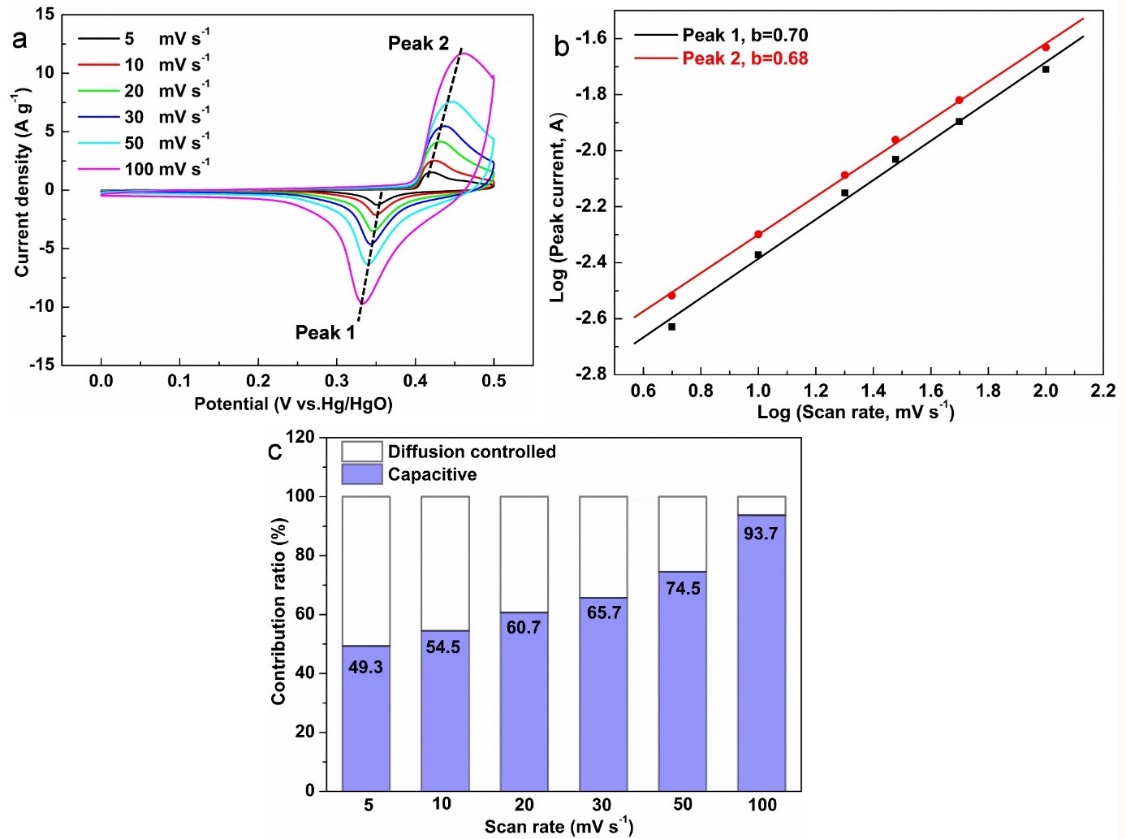


**Figure S30.** a) CV curves of the M7 at various scan rates of 5-100 mV s^-1^ in a three-electrode cell. b) Log*(i)* versus log*(v)* plots of the M7 at specific peak currents. c) Bar chart showing the percent of pseudocapacitive contribution of the M7 at different scan rates.

1. **Pseudocapacitive contribution shadow diagram of M7 in CV curves**


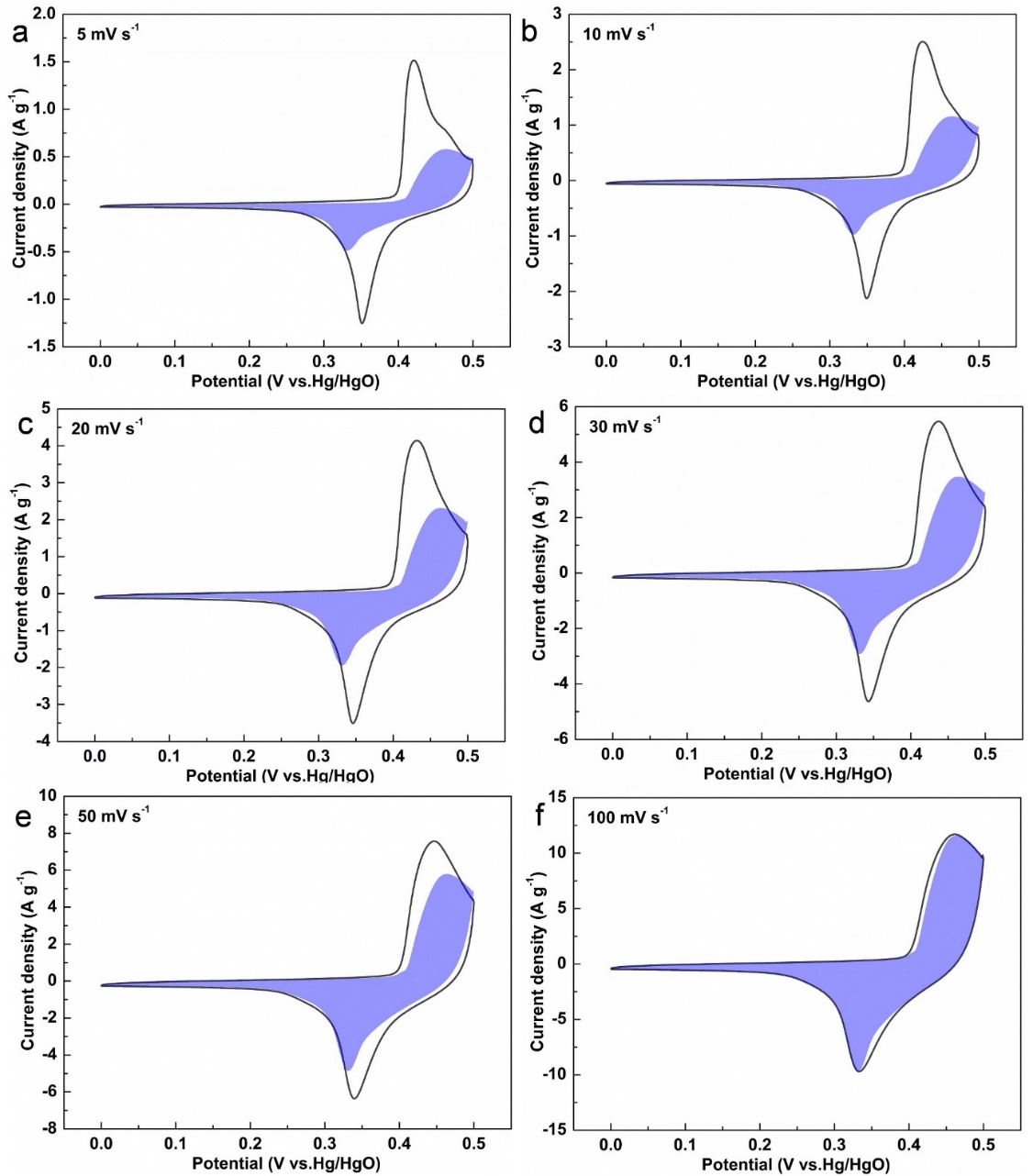


**Figure S31.** CV curve with the pseudocapacitive fraction shown by the shaded area of M7 at various scan rates in a three-electrode cell. a) 5 mV s^-1^. b) 10 mV s^-1^. c) 20 mV s^-1^. d) 30 mV s^-1^. e) 50 mV s^-1^. f) 100 mV s^-1^.

1. **The analysis of ion-diffusion and capacitive contributions of the M8**


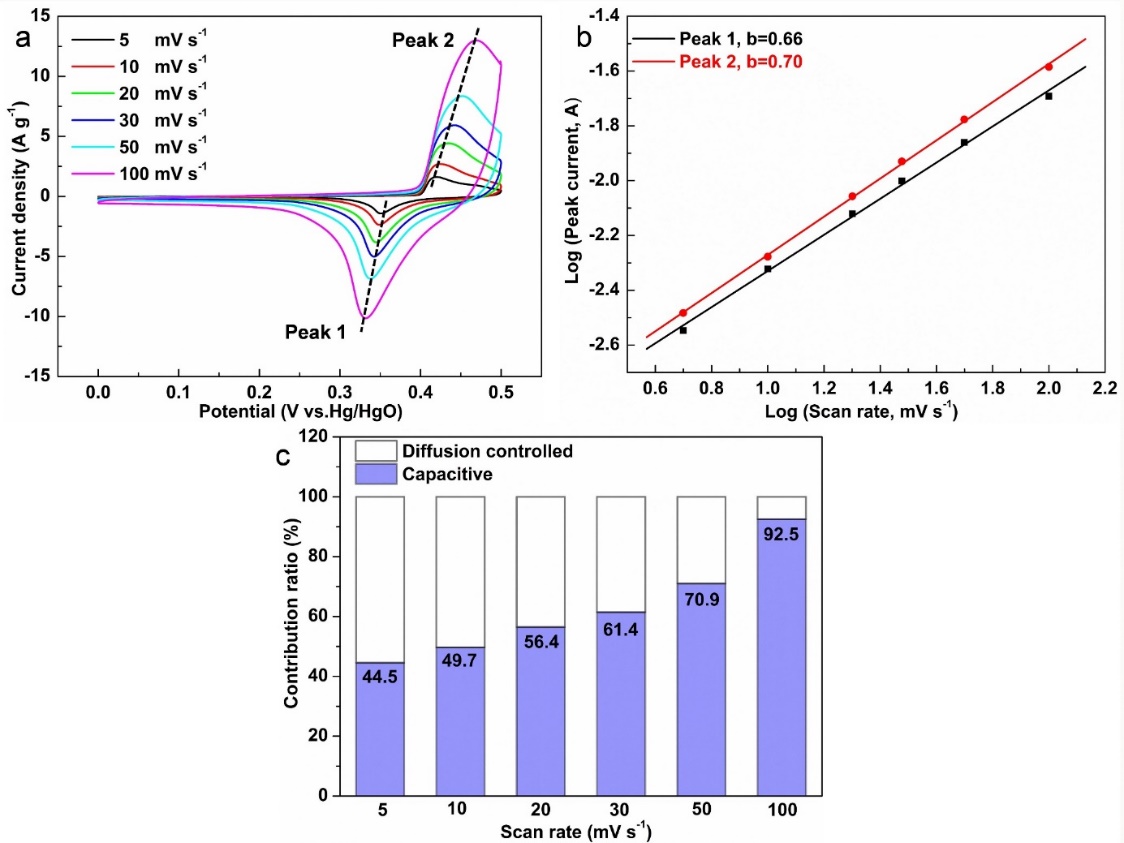


**Figure S32.** a) CV curves of the M8 at various scan rates of 5-100 mV s^-1^ in a three-electrode cell. b) Log*(i)* versus log*(v)* plots of the M8 at specific peak currents. c) Bar chart showing the percent of pseudocapacitive contribution of the M8 at different scan rates.

1. **Pseudocapacitive contribution shadow diagram of M8 in CV curves**


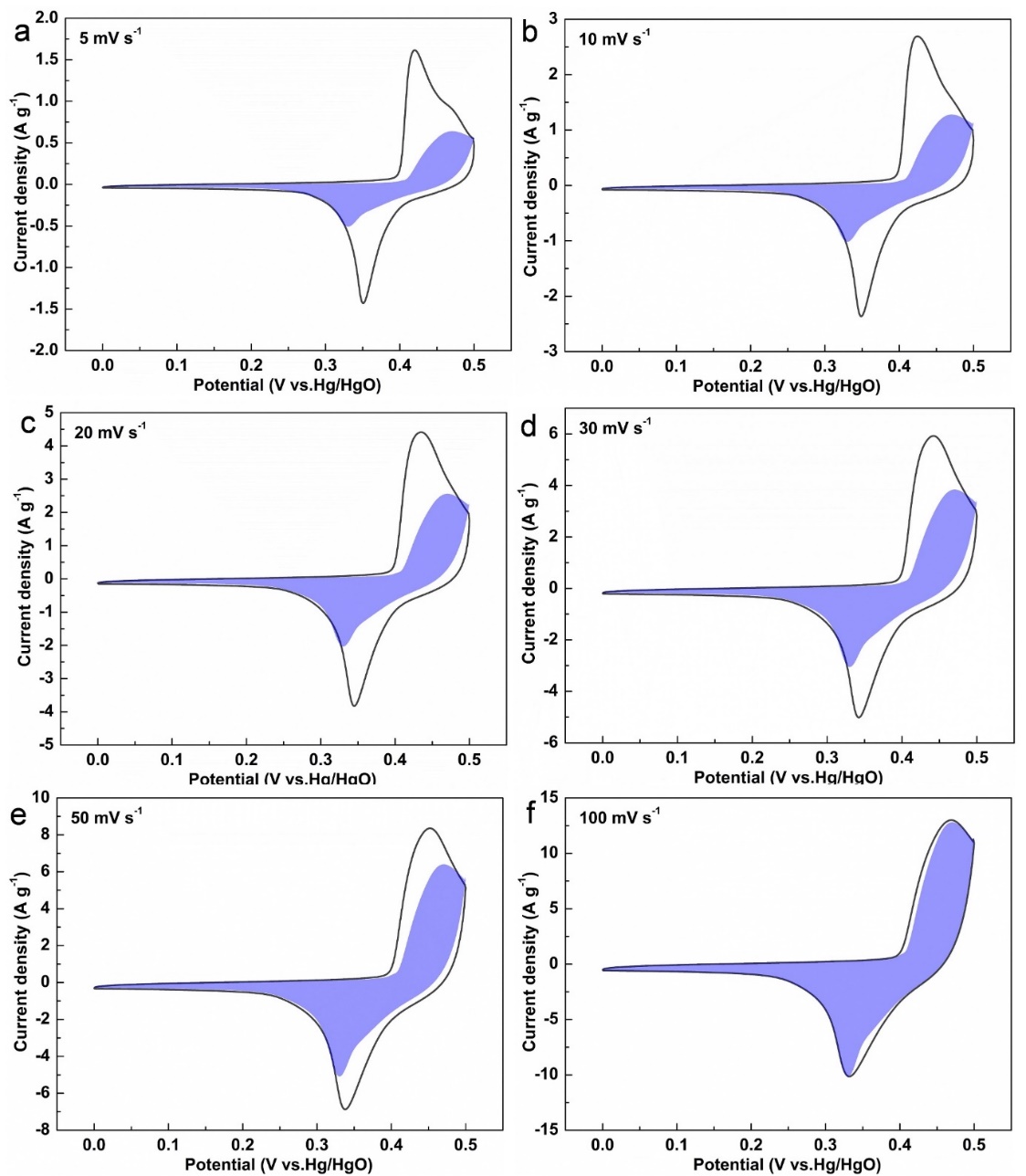


**Figure S33.** CV curve with the pseudocapacitive fraction shown by the shaded area of M8 at various scan rates in a three-electrode cell. a) 5 mV s^-1^. b) 10 mV s^-1^. c) 20 mV s^-1^. d) 30 mV s^-1^. e) 50 mV s^-1^. f) 100 mV s^-1^.

1. **The percent of the diffusion-controlled capacity and pseudocapacitive contribution of the M1-M8**


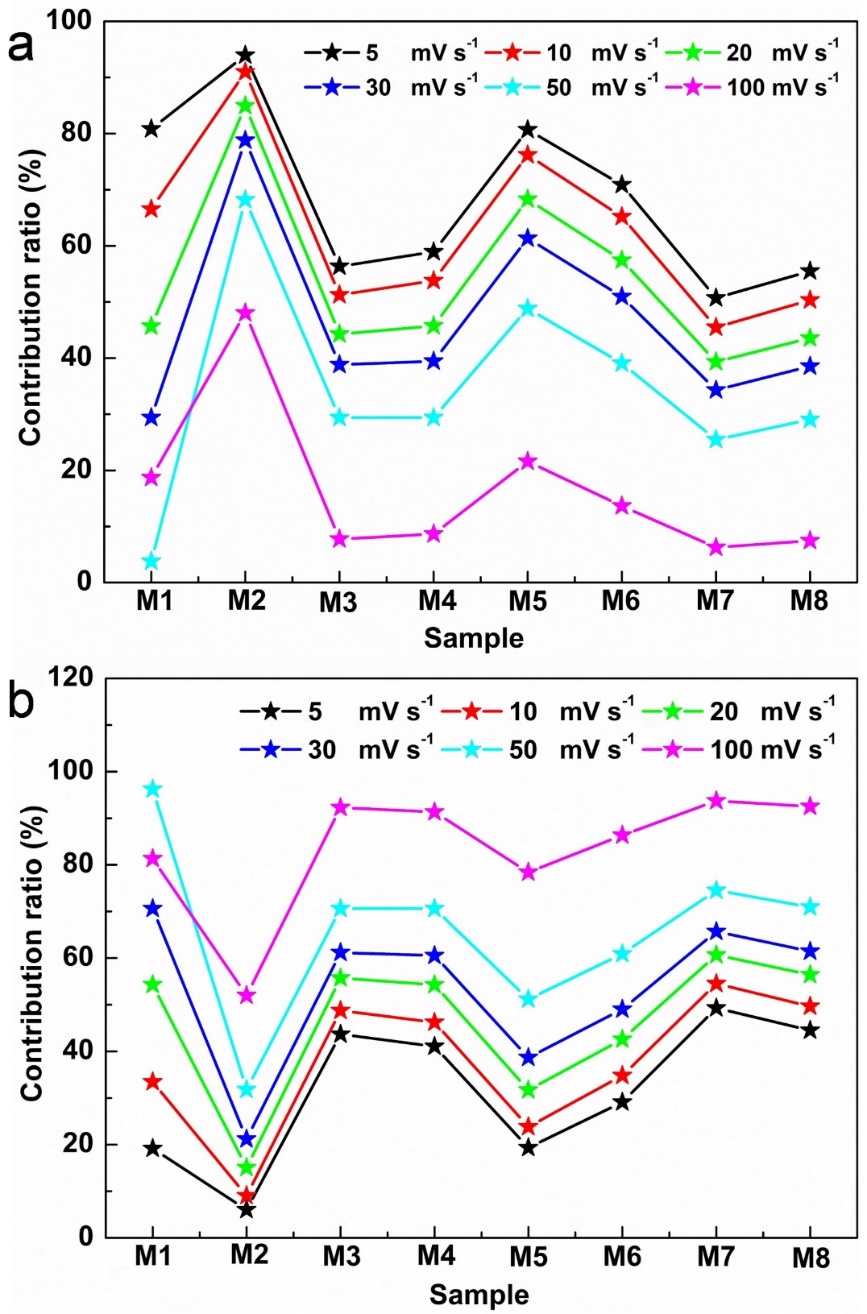


**Figure S34.** a) The percent of the diffusion-controlled capacity contribution of the M1-M8 at different scan rates. b) The percent of pseudocapacitive contribution of the M1-M8 at different scan rates.

1. **CV and GCD curves of the M5 at different potentials**


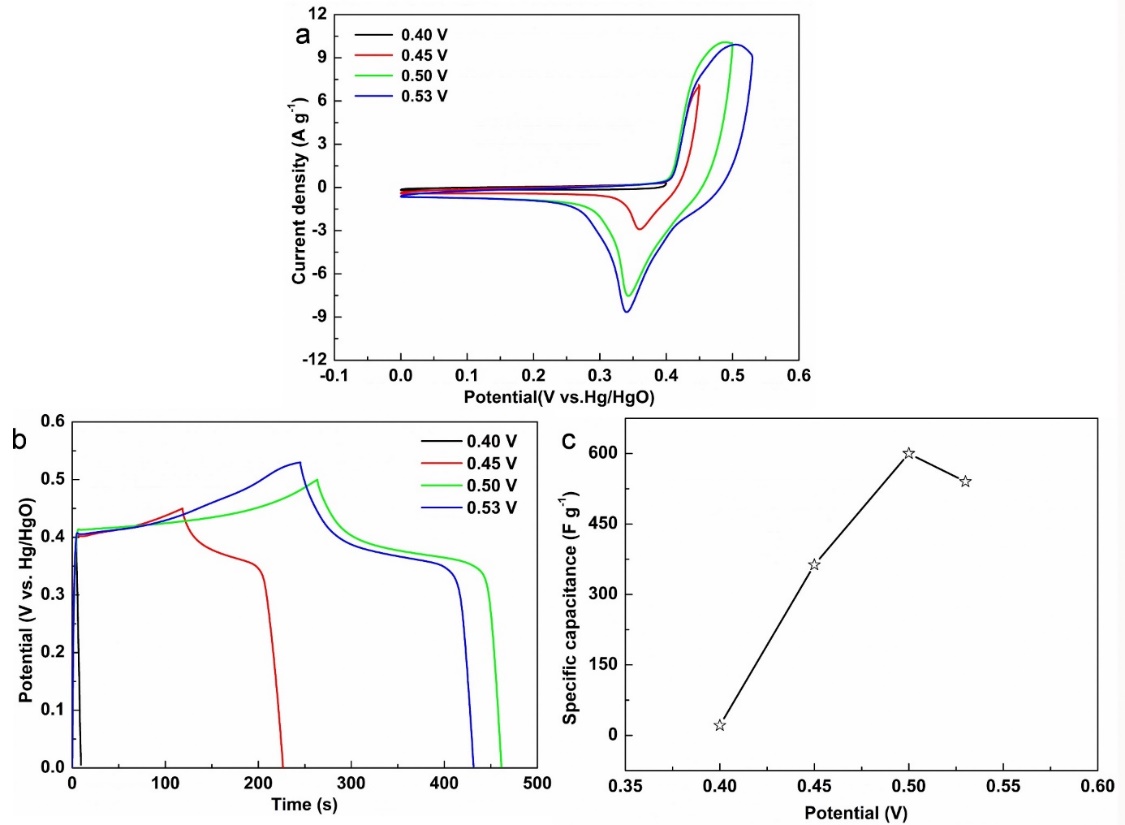


**Figure S35.** a) CV curves of the M5 with a scan rate at 30 mV s^-1^ at different potentials in a three-electrode cell. b) The GCD curves of M5 with a current density 1.5 A g^-1^ at different potentials. c) Specific capacitance change vs. potential.

1. **The GCD curves of M1-M4 at different current densities**


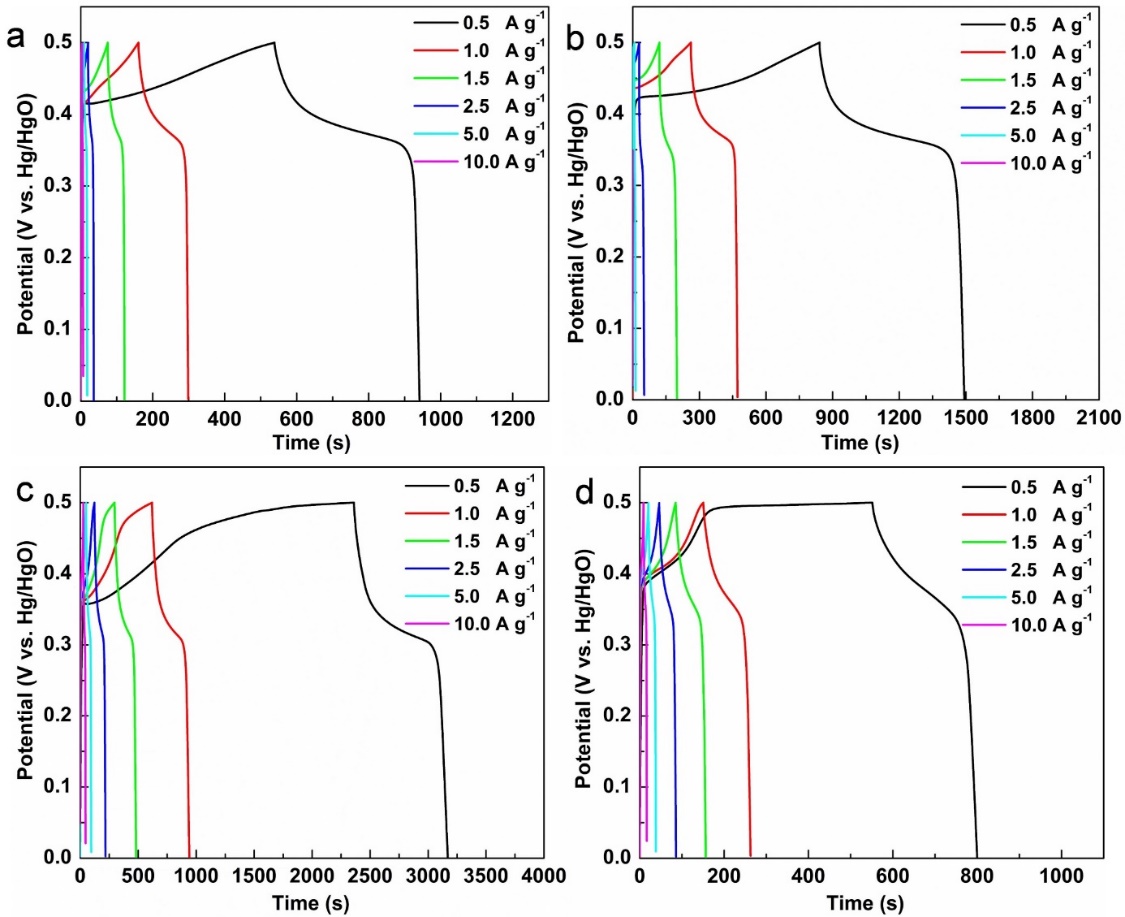


**Figure S36.** The GCD curves at different current densities in a three-electrode cell. a) M1. b) M2. c) M3. d) M4.

1. **The GCD curves of M5-M8 at different current densities**


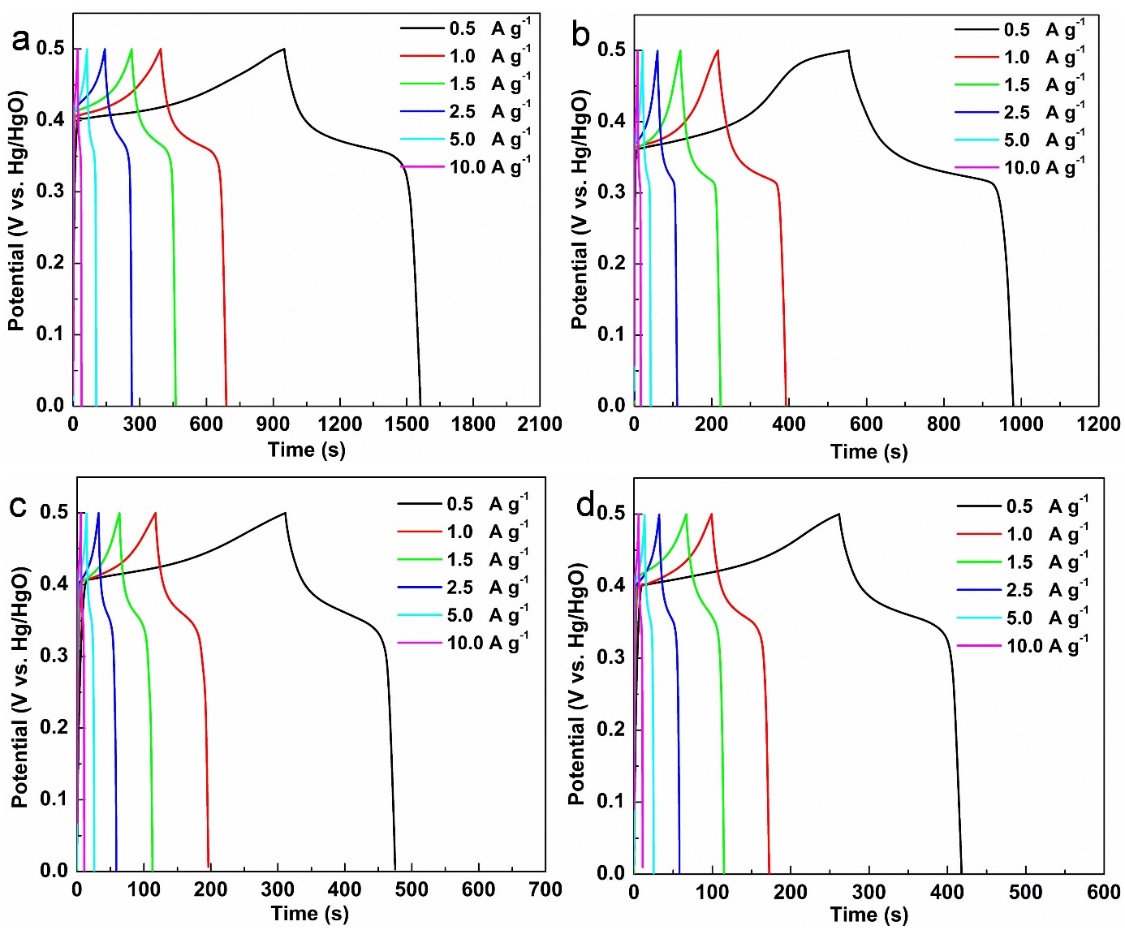


**Figure S37.** The GCD curves at different current densities in a three-electrode cell. a) M5. b) M6. c) M7. d) M8.

1. **The specific capacitance of the M1-M8 at different current densities.**


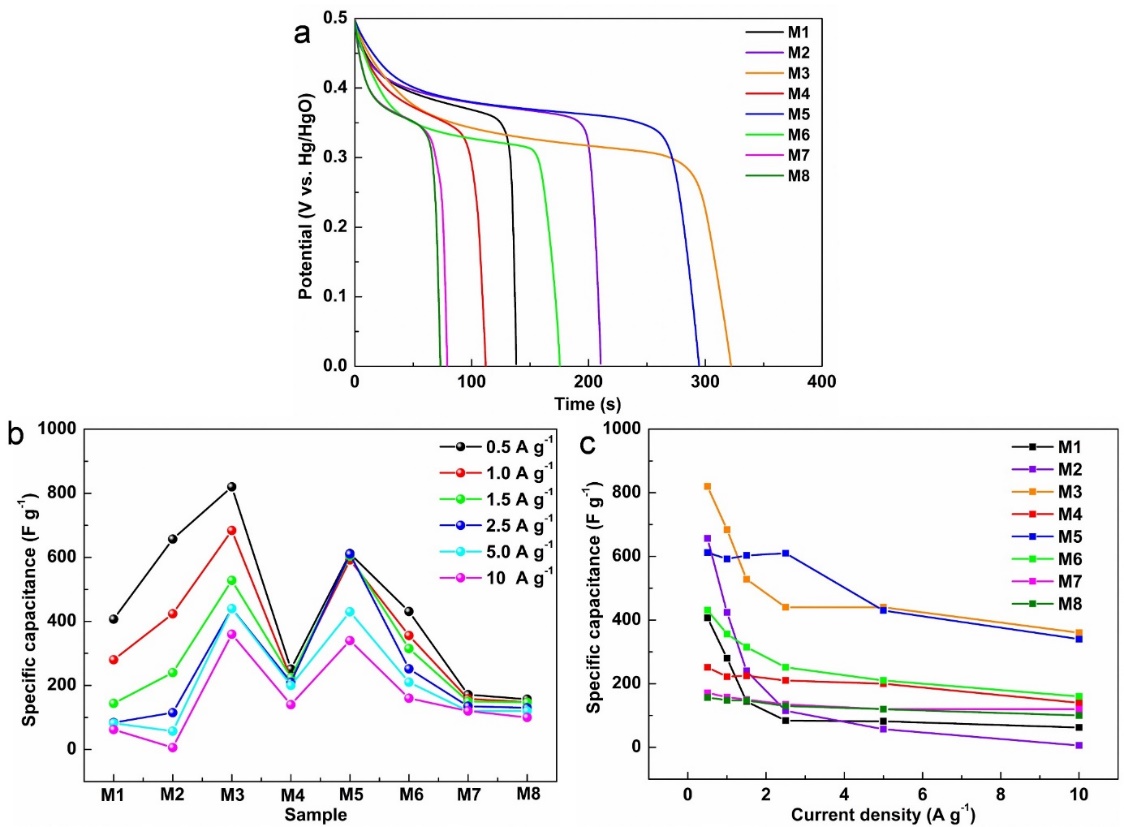


**Figure S38.** a) The galvanostatic discharge curves of the M1-M8 at 1 A g^-1^. b,c) The specific capacitance of the M1-M8 at different current densities.

1. **The specific capacitance of organic ligand**


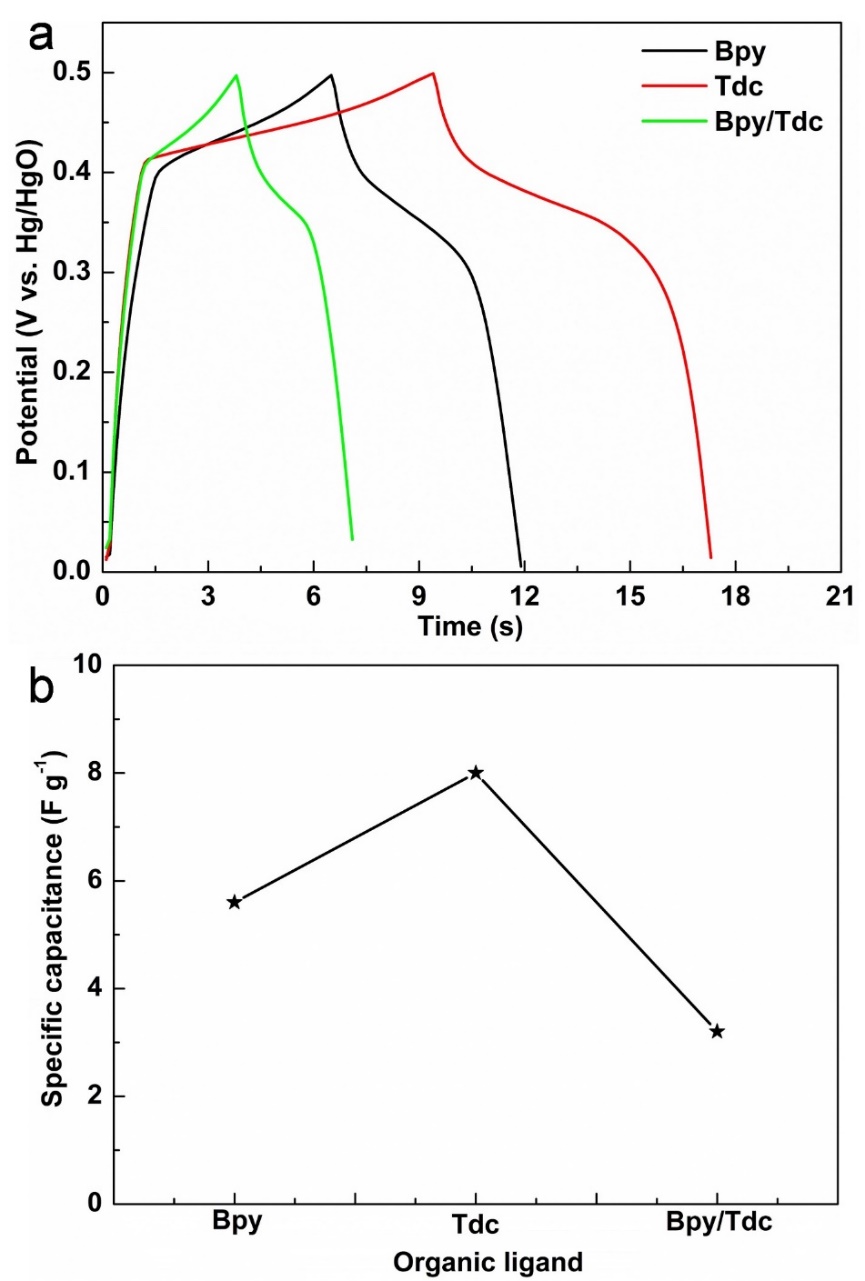


**Figure S39.** a) The GCD curves of organic ligand at 0.5 A g^-1^ in a three-electrode cell. b) Specific capacitance change vs. organic ligand.

1. **The electrochemical impedance spectra of M1-M8**


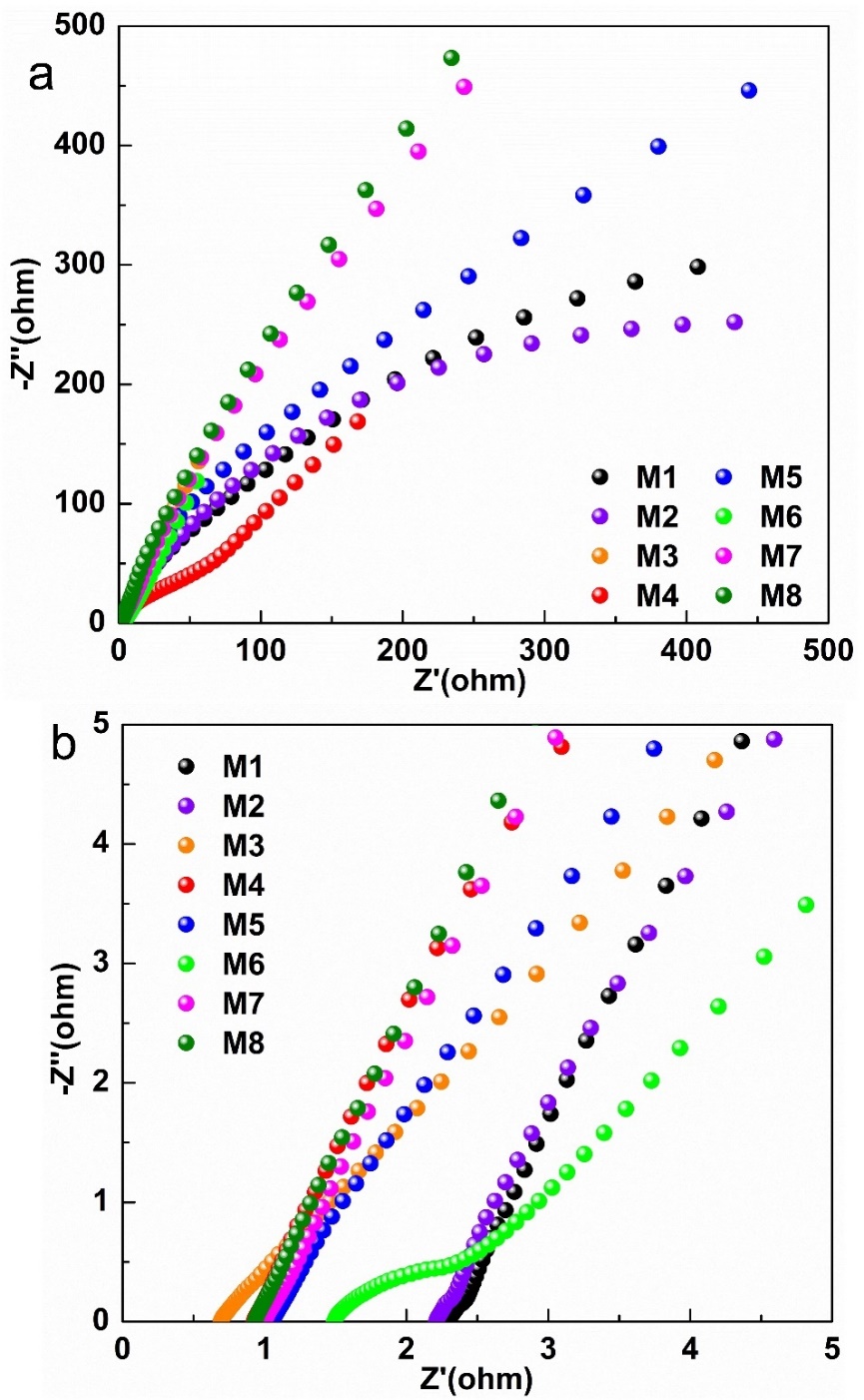


**Figure S40.** Nyquist plots of M1-M8 in a three-electrode cell.

1. **The GCD curves of AC**





**Figure S41.** The GCD curves of AC at 1 A g^-1^.

1. **The analysis of ion-diffusion and capacitive contributions of the M1//AC**


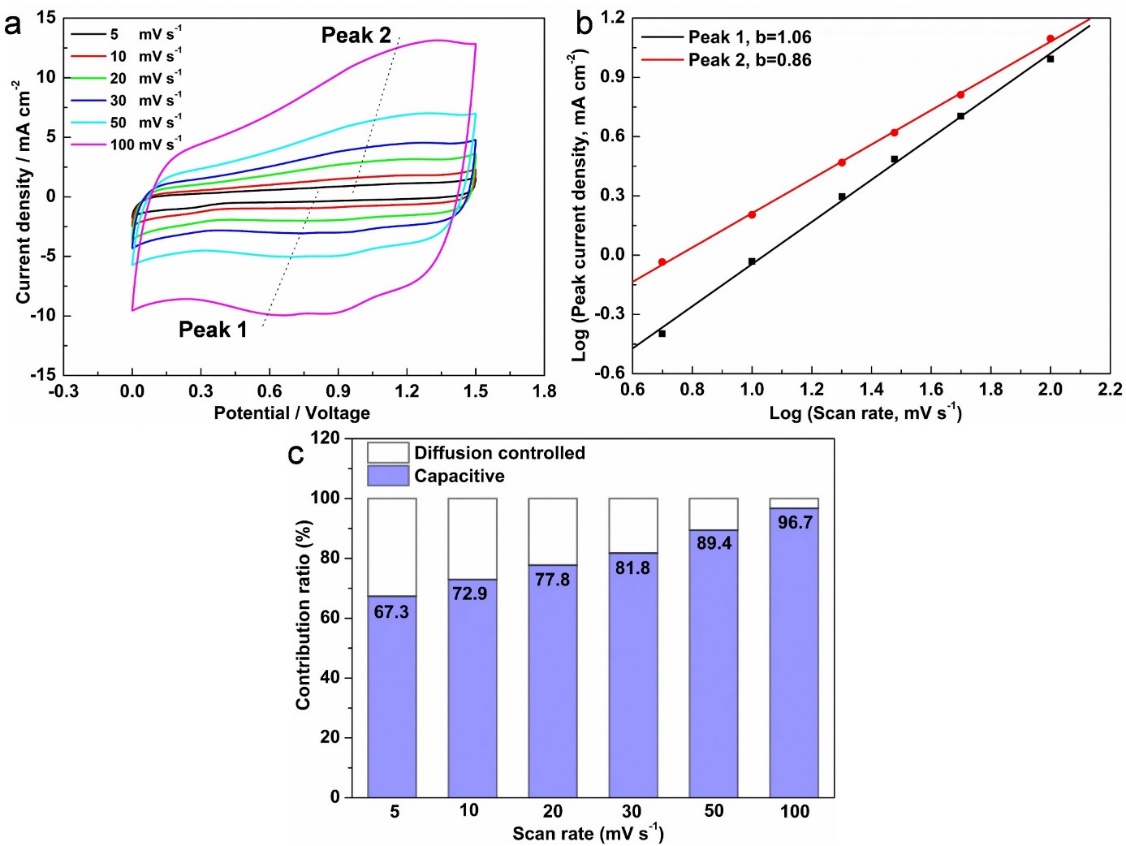


**Figure S42.** a) CV curves of the M1//AC at various scan rates of 5-100 mV s^-1^. b) Log*(i)* versus log*(v)* plots of the M1//AC at specific peak currents. c) Bar chart showing the percent of pseudocapacitive contribution of the M1//AC at different scan rates.

1. **Pseudocapacitive contribution shadow diagram of M1//AC in CV curves**


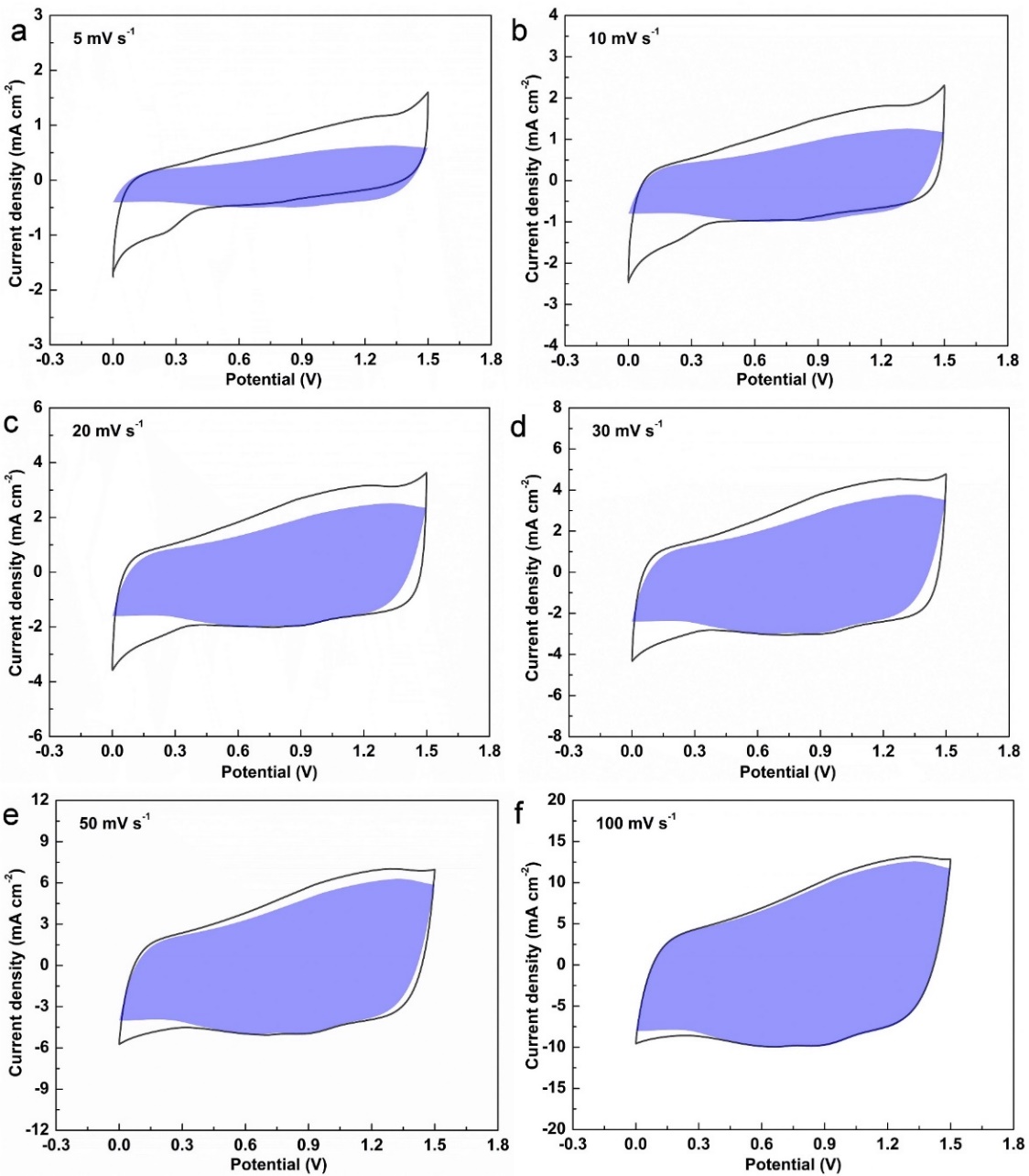


**Figure S43.** CV curve with the pseudocapacitive fraction shown by the shaded area of M1//AC at various scan rates. a) 5 mV s^-1^. b) 10 mV s^-1^. c) 20 mV s^-1^. d) 30 mV s^-1^. e) 50 mV s^-1^. f) 100 mV s^-1^.

1. **The analysis of ion-diffusion and capacitive contributions of the M2//AC**


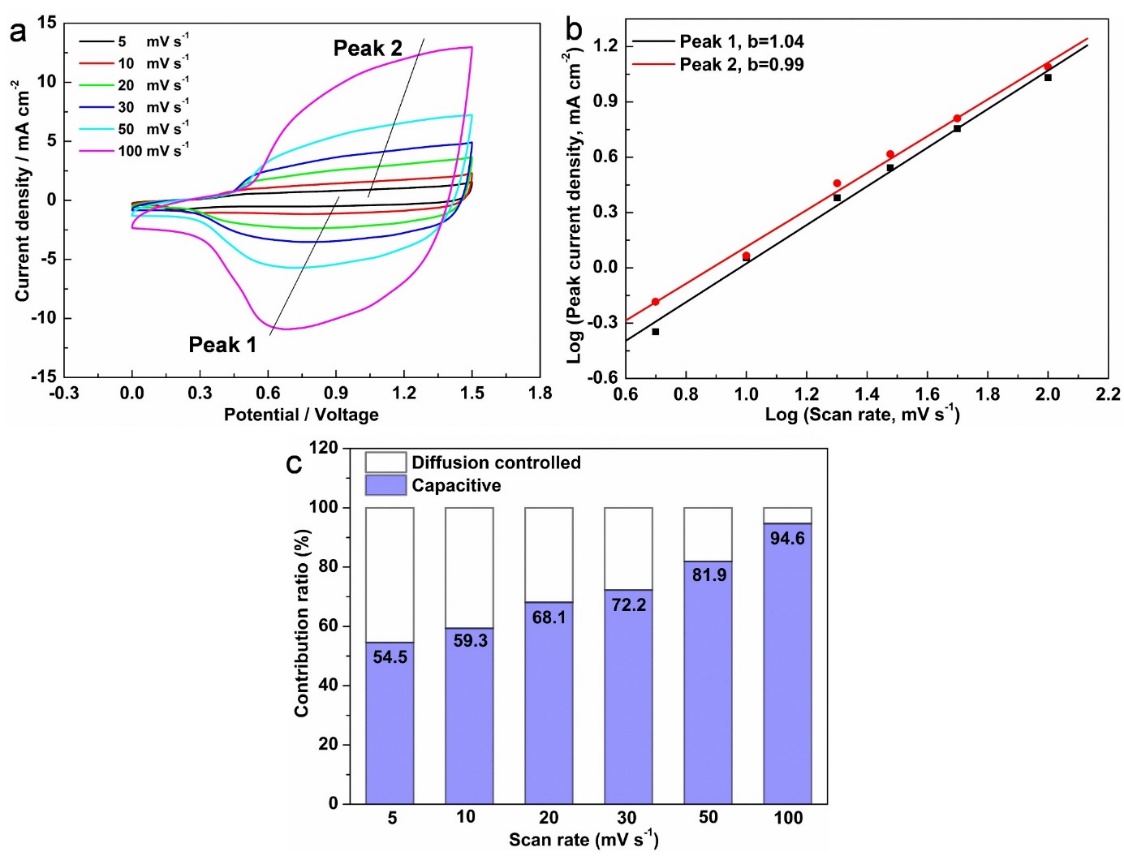


**Figure S44.** a) CV curves of the M2//AC at various scan rates of 5-100 mV s^-1^. b) Log*(i)* versus log*(v)* plots of the M2//AC at specific peak currents. c) Bar chart showing the percent of pseudocapacitive contribution of the M2//AC at different scan rates.

1. **Pseudocapacitive contribution shadow diagram of M2//AC in CV curves**


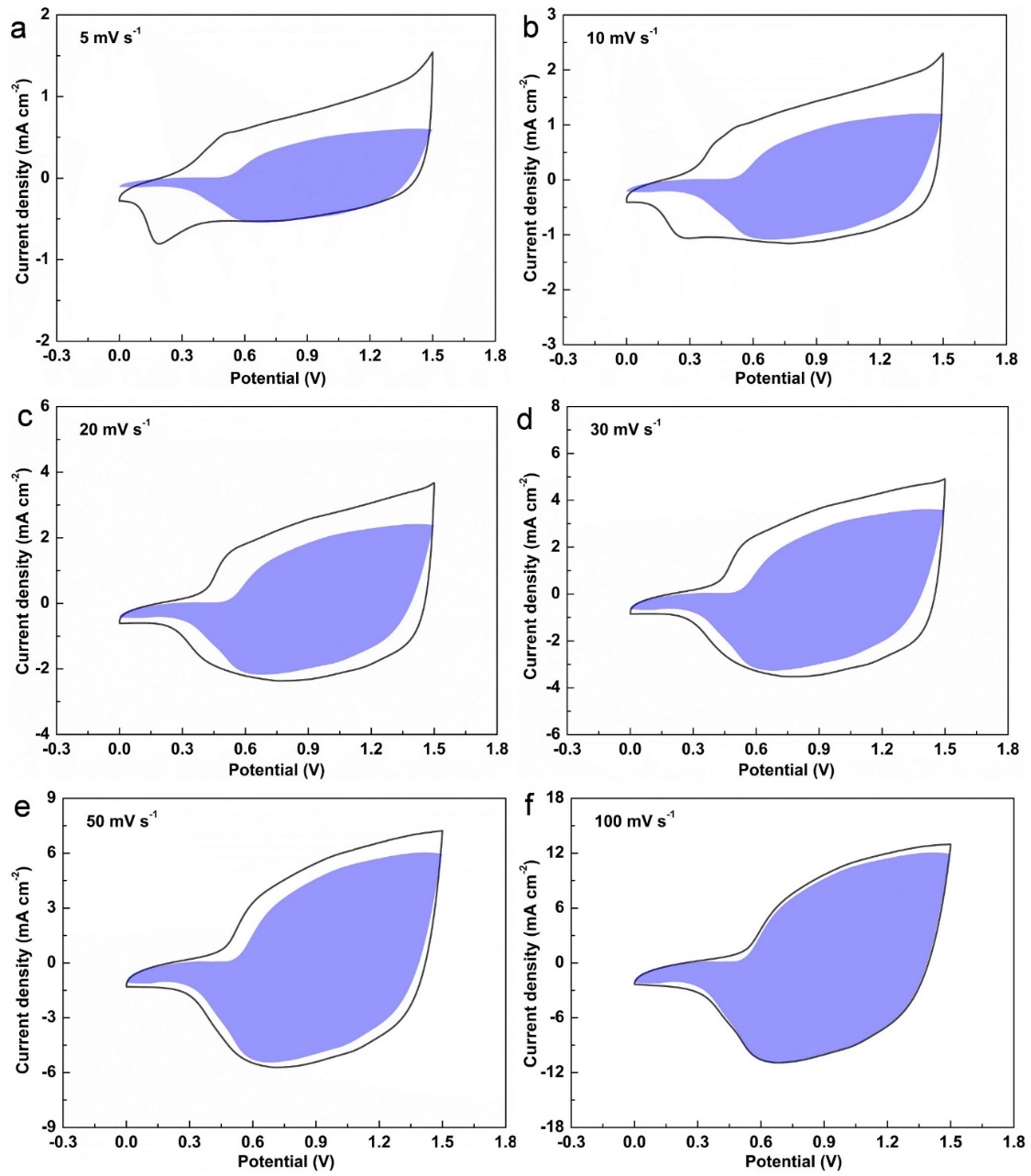


**Figure S45.** CV curve with the pseudocapacitive fraction shown by the shaded area of M2//AC at various scan rates. a) 5 mV s^-1^. b) 10 mV s^-1^. c) 20 mV s^-1^. d) 30 mV s^-1^. e) 50 mV s^-1^. f) 100 mV s^-1^.

1. **The analysis of ion-diffusion and capacitive contributions of the M3//AC**


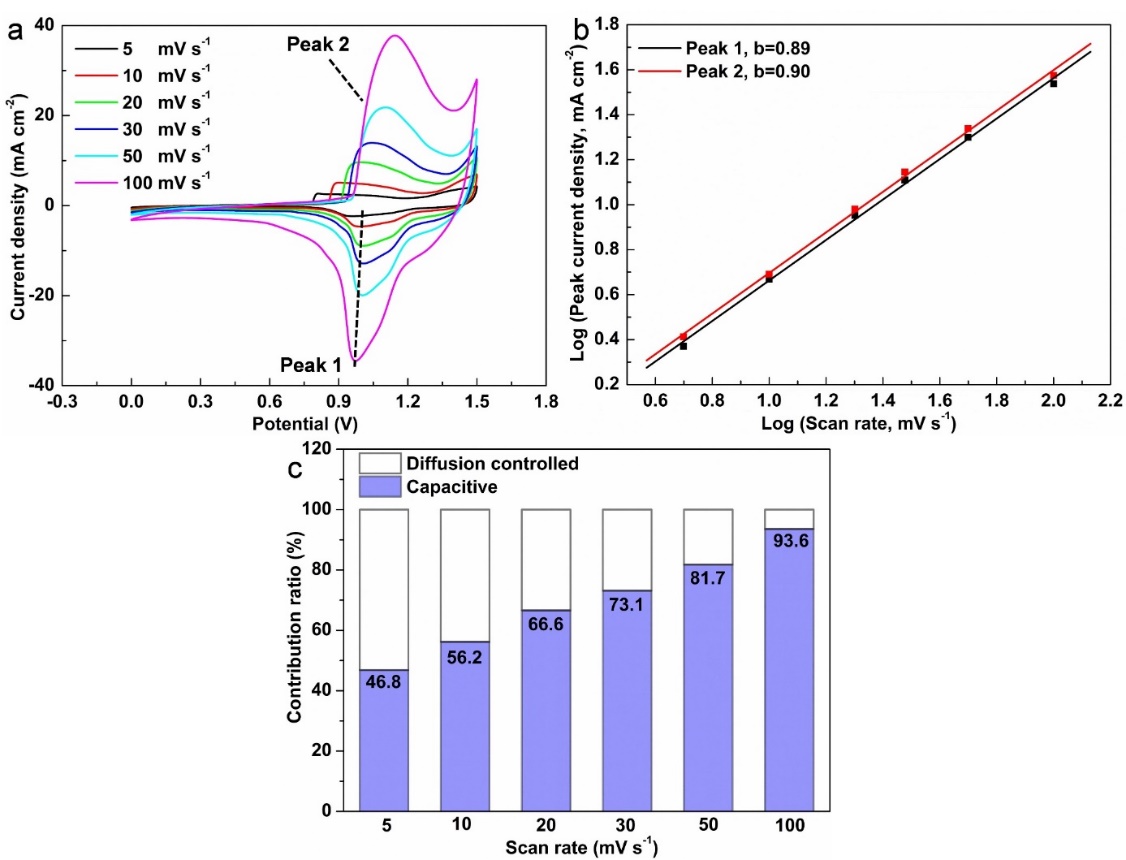


**Figure S46.** a) CV curves of the M3//AC at various scan rates of 5-100 mV s^-1^. b) Log*(i)* versus log*(v)* plots of the M3//AC at specific peak currents. c) Bar chart showing the percent of pseudocapacitive contribution of the M3//AC at different scan rates.

1. **Pseudocapacitive contribution shadow diagram of M3//AC in CV curves**


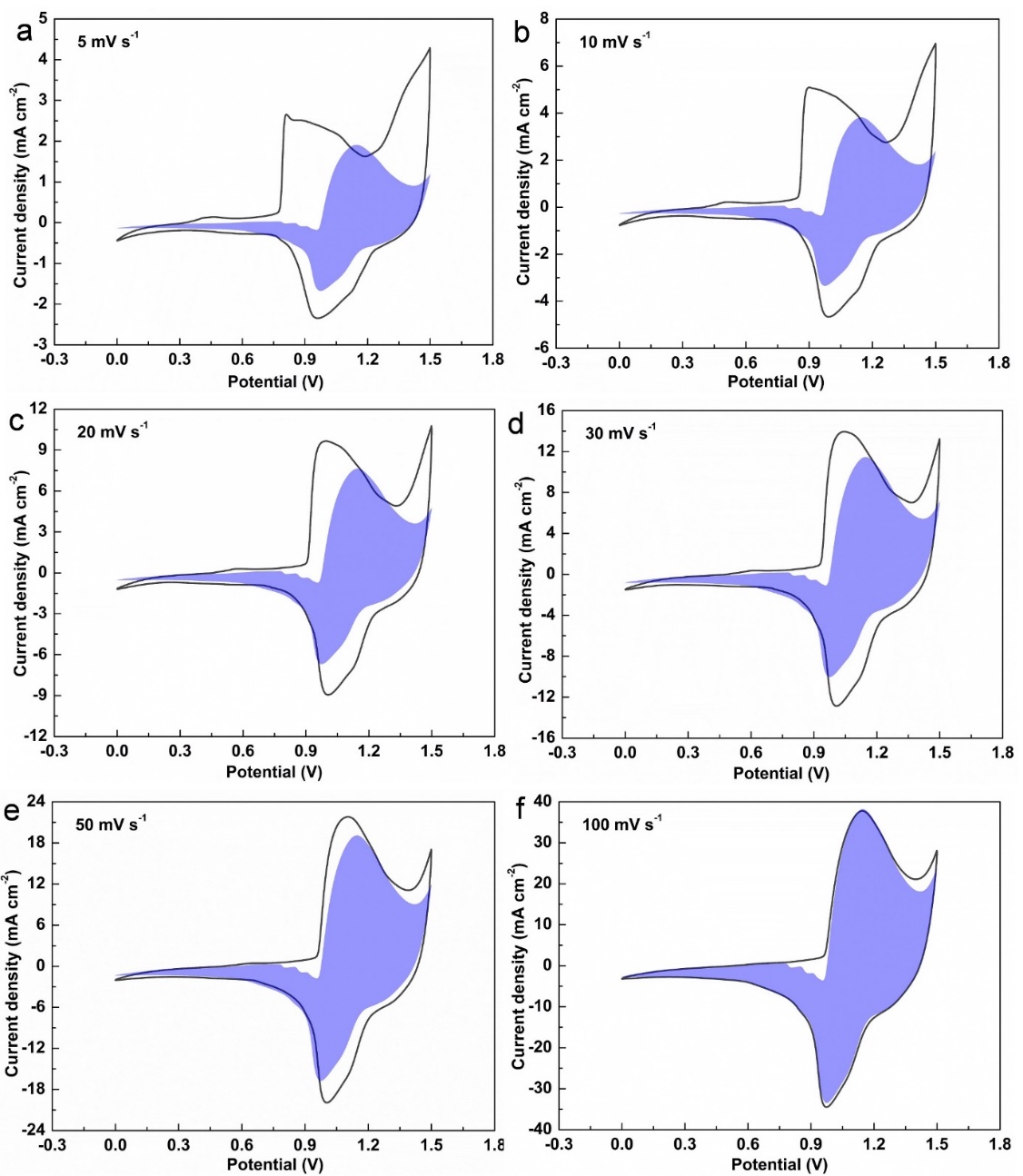


**Figure S47.** CV curve with the pseudocapacitive fraction shown by the shaded area of M3//AC at various scan rates. a) 5 mV s^-1^. b) 10 mV s^-1^. c) 20 mV s^-1^. d) 30 mV s^-1^. e) 50 mV s^-1^. f) 100 mV s^-1^.

1. **The analysis of ion-diffusion and capacitive contributions of the M4//AC**


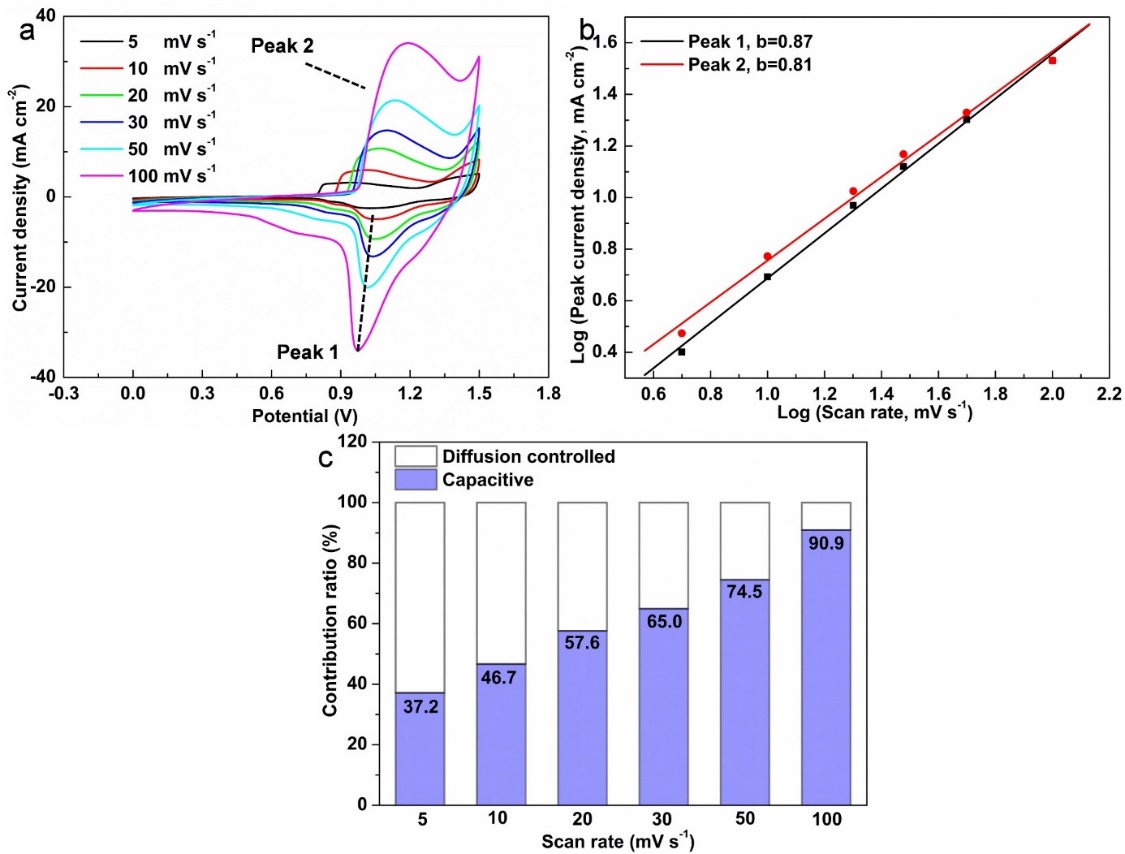


**Figure S48.** a) CV curves of the M4//AC at various scan rates of 5-100 mV s^-1^. b) Log*(i)* versus log*(v)* plots of the M4//AC at specific peak currents. c) Bar chart showing the percent of pseudocapacitive contribution of the M4//AC at different scan rates.

1. **Pseudocapacitive contribution shadow diagram of M4//AC in CV curves**


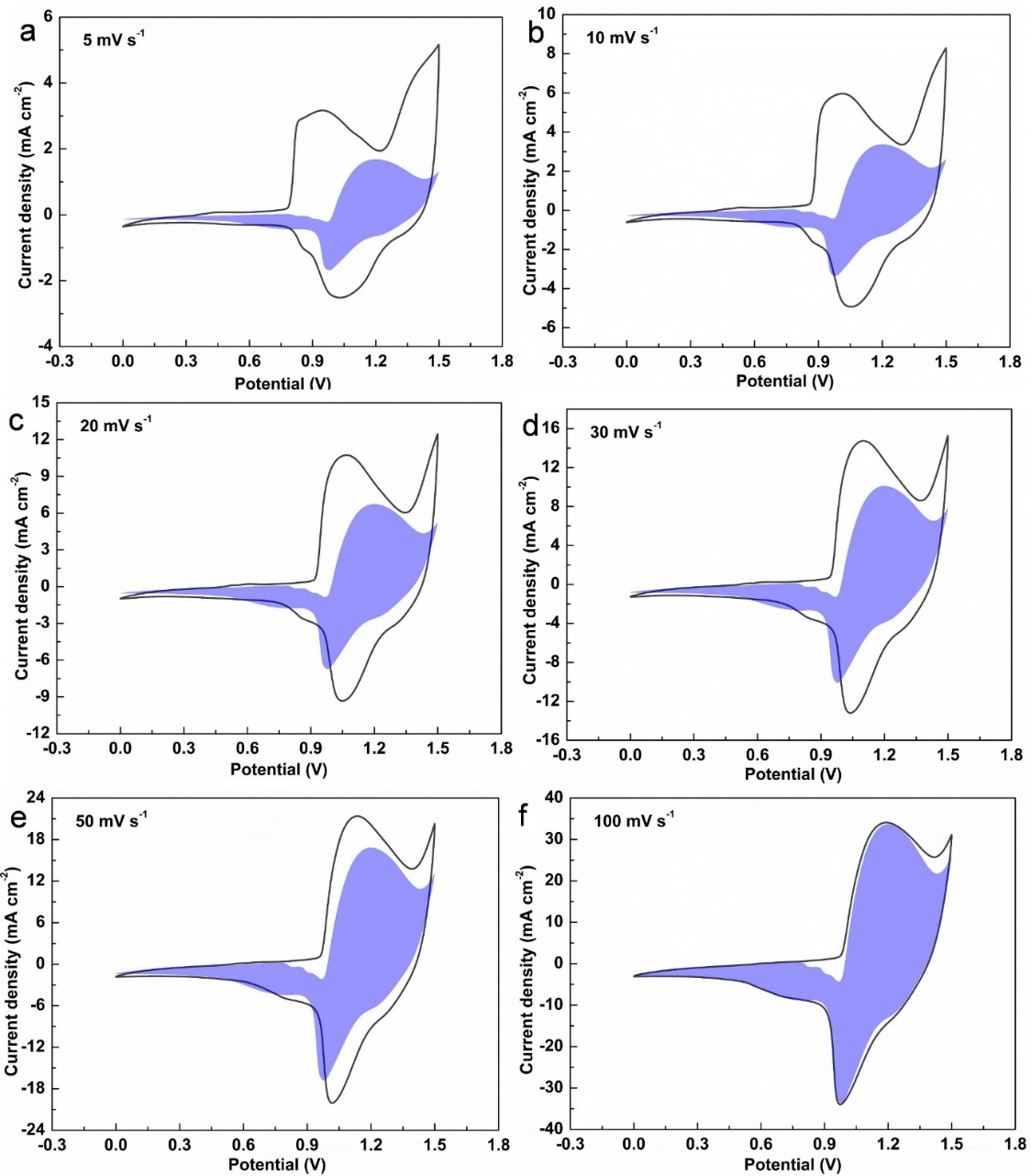


**Figure S49.** CV curve with the pseudocapacitive fraction shown by the shaded area of M4//AC at various scan rates. a) 5 mV s^-1^. b) 10 mV s^-1^. c) 20 mV s^-1^. d) 30 mV s^-1^. e) 50 mV s^-1^. f) 100 mV s^-1^.

1. **The analysis of capacitive contributions of the M5//AC**





**Figure S50.** Bar chart showing the percent of pseudocapacitive contribution of the M5//AC at different scan rates.

1. **Pseudocapacitive contribution shadow diagram of M5//AC in CV curves**


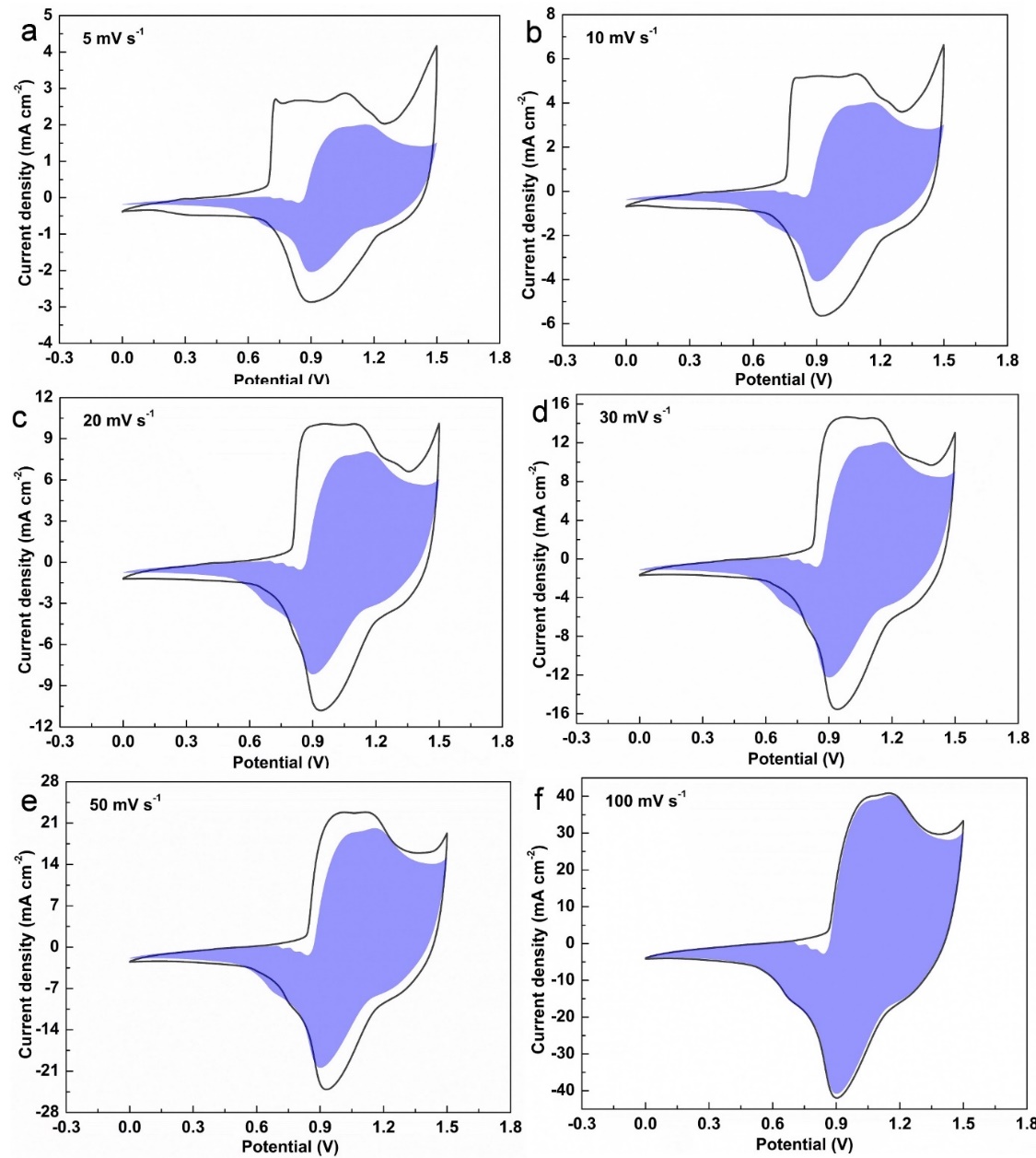


**Figure S51.** CV curve with the pseudocapacitive fraction shown by the shaded area of M5//AC at various scan rates. a) 5 mV s^-1^. b) 10 mV s^-1^. c) 20 mV s^-1^. d) 30 mV s^-1^. e) 50 mV s^-1^. f) 100 mV s^-1^.

1. **The analysis of ion-diffusion and capacitive contributions of the M6//AC**


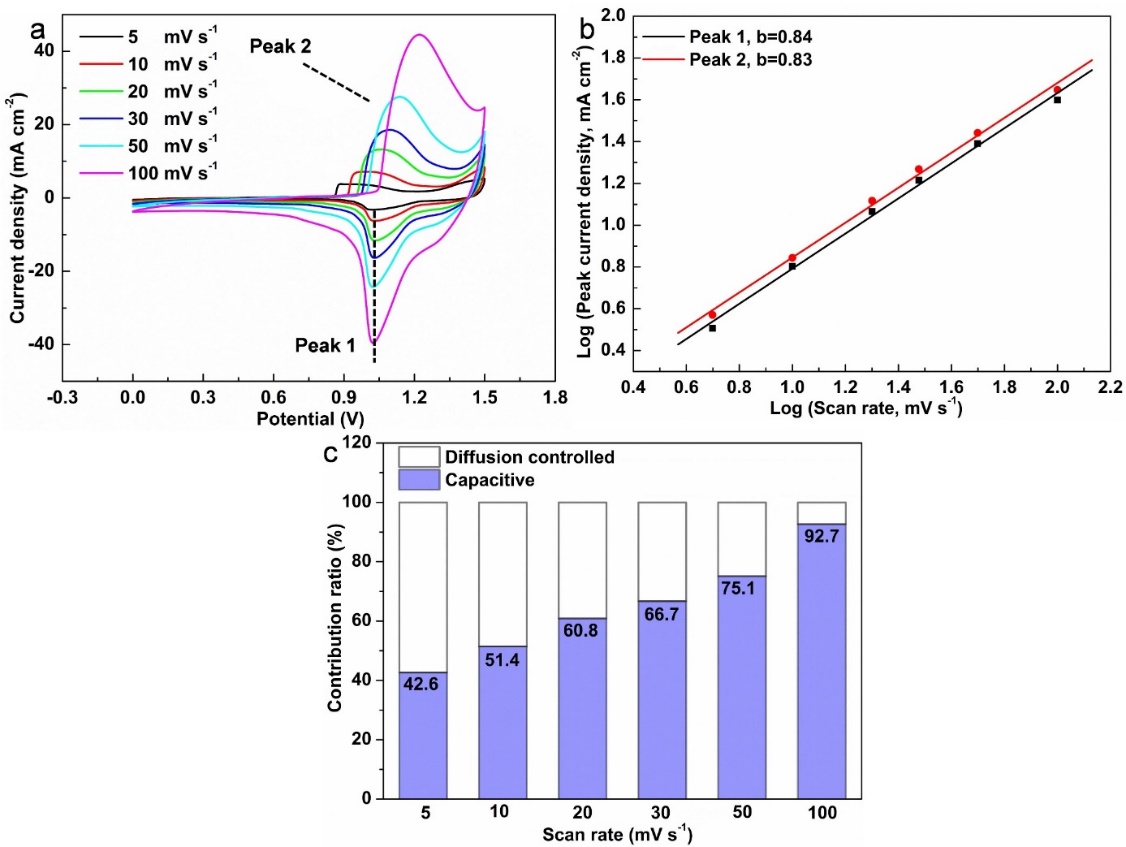


**Figure S52.** a) CV curves of the M6//AC at various scan rates of 5-100 mV s^-1^. b) Log*(i)* versus log*(v)* plots of the M6//AC at specific peak currents. c) Bar chart showing the percent of pseudocapacitive contribution of the M6//AC at different scan rates.

1. **Pseudocapacitive contribution shadow diagram of M6//AC in CV curves**


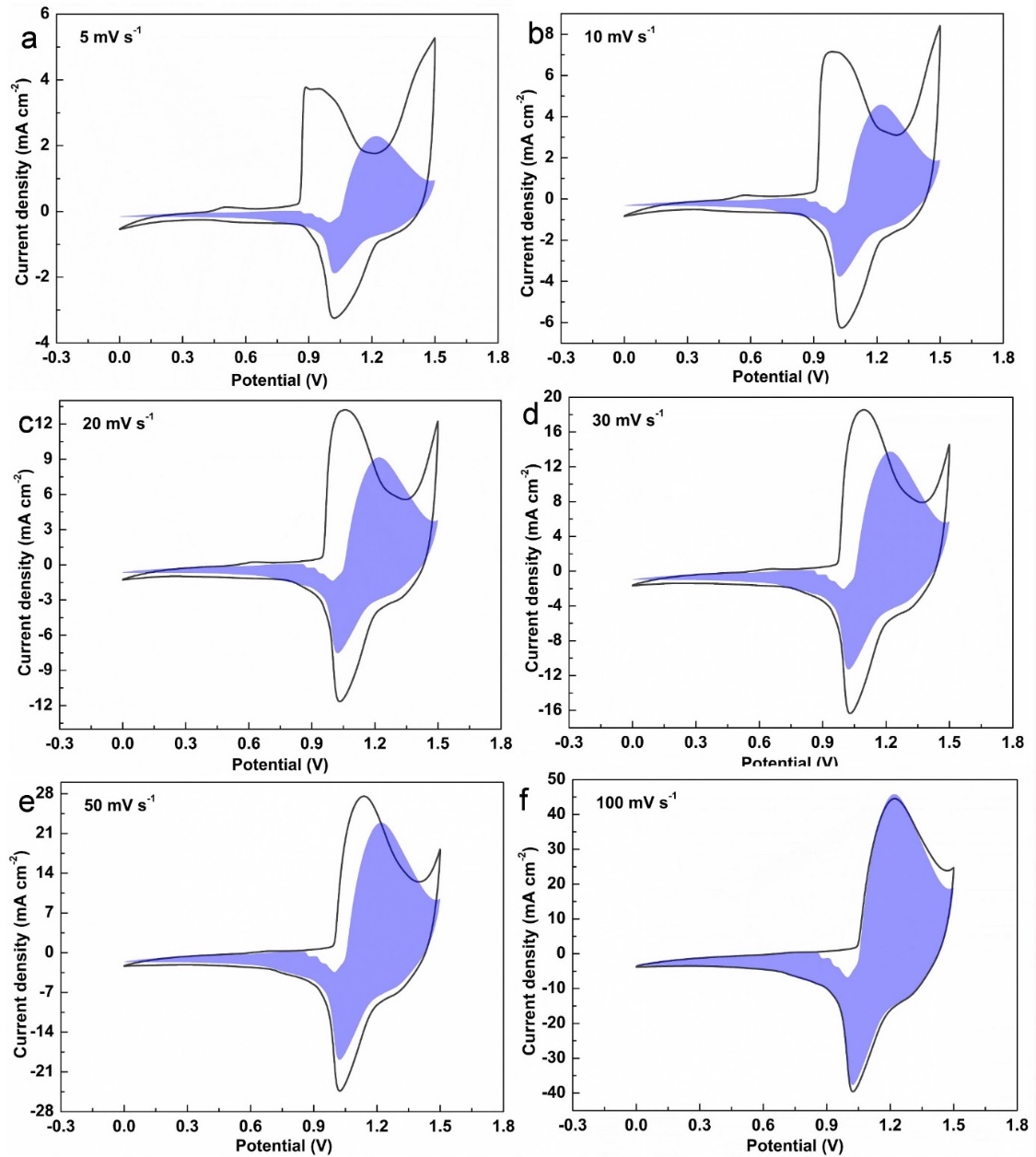


**Figure S53.** CV curve with the pseudocapacitive fraction shown by the shaded area of M6//AC at various scan rates. a) 5 mV s^-1^. b) 10 mV s^-1^. c) 20 mV s^-1^. d) 30 mV s^-1^. e) 50 mV s^-1^. f) 100 mV s^-1^.

1. **The analysis of ion-diffusion and capacitive contributions of the M7//AC**


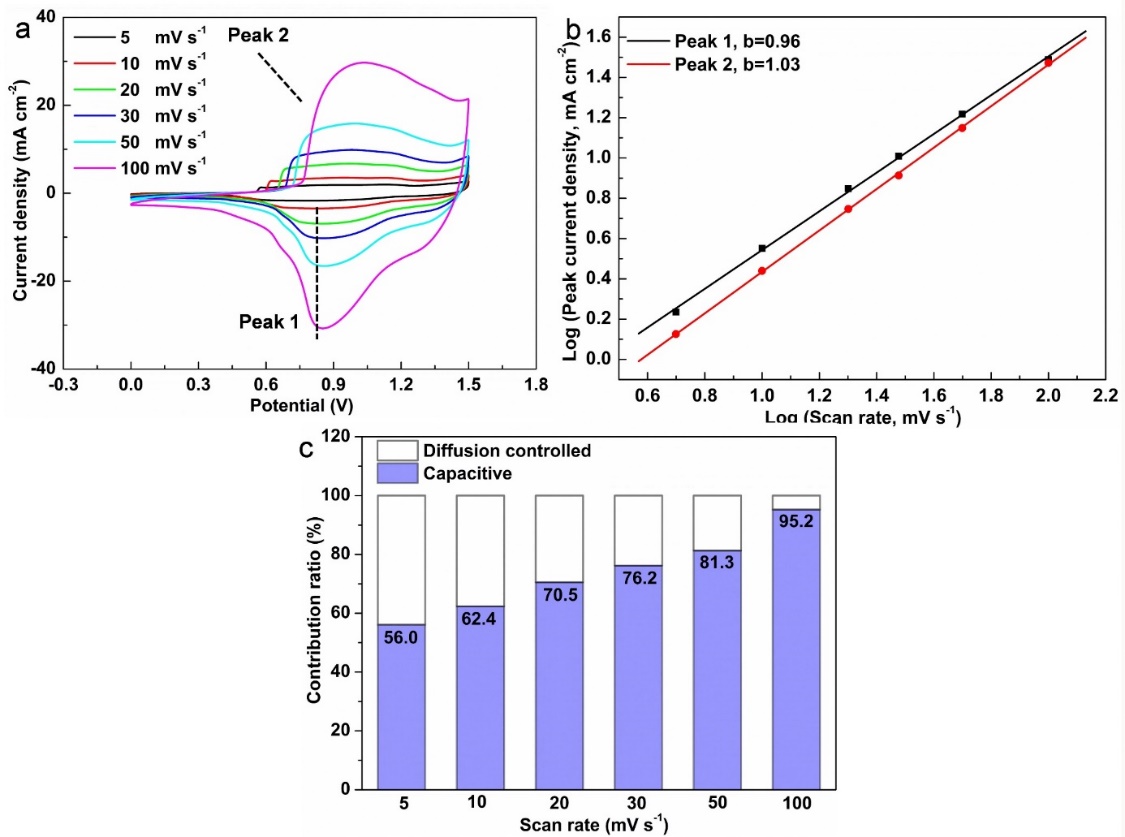


**Figure S54.** a) CV curves of the M7//AC at various scan rates of 5-100 mV s^-1^. b) Log*(i)* versus log*(v)* plots of the M7//AC at specific peak currents. c) Bar chart showing the percent of pseudocapacitive contribution of the M7//AC at different scan rates.

1. **Pseudocapacitive contribution shadow diagram of M7//AC in CV curves**


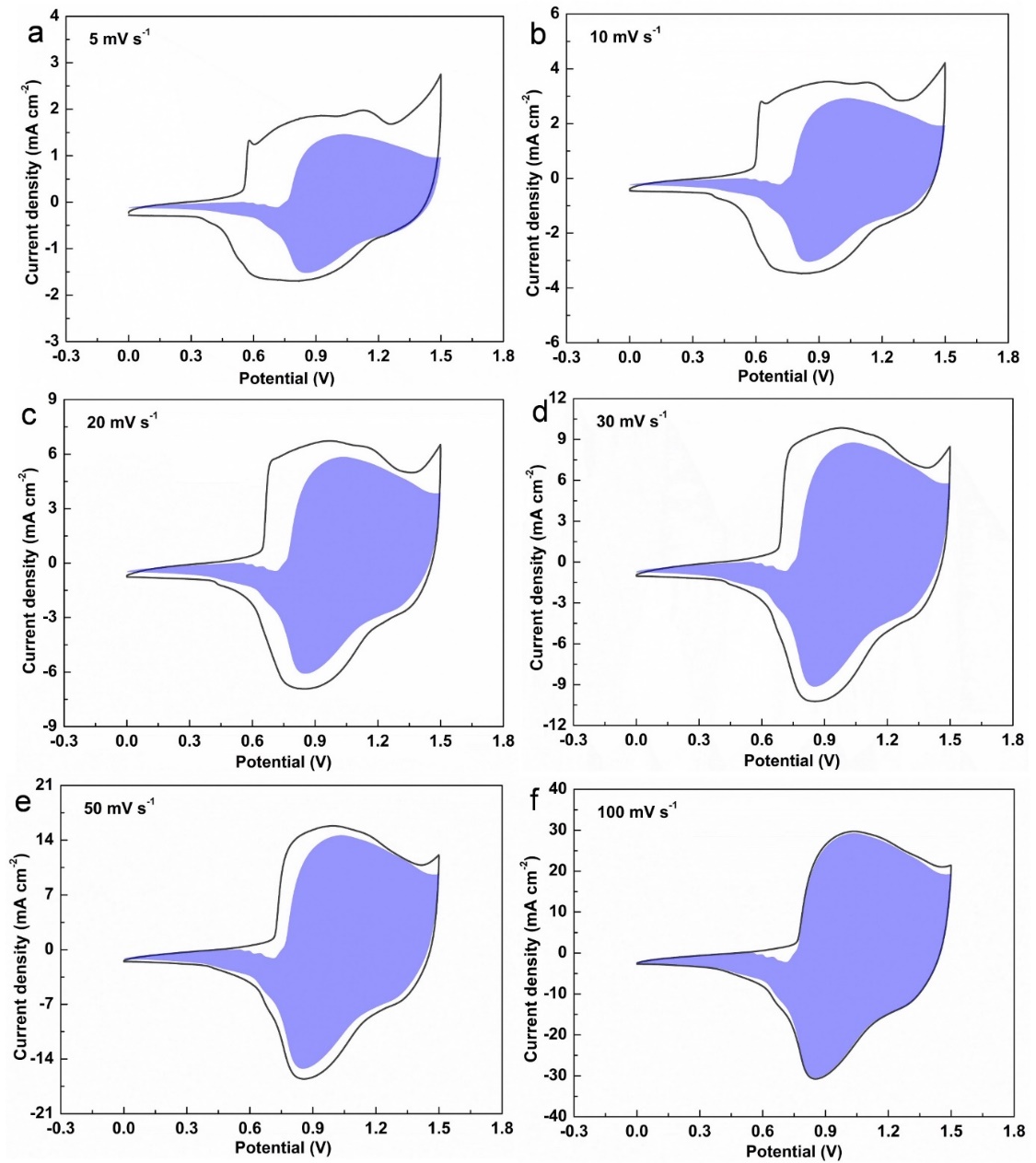


**Figure S55.** CV curve with the pseudocapacitive fraction shown by the shaded area of M7//AC at various scan rates. a) 5 mV s^-1^. b) 10 mV s^-1^. c) 20 mV s^-1^. d) 30 mV s^-1^. e) 50 mV s^-1^. f) 100 mV s^-1^.

1. **The analysis of ion-diffusion and capacitive contributions of the M8//AC**


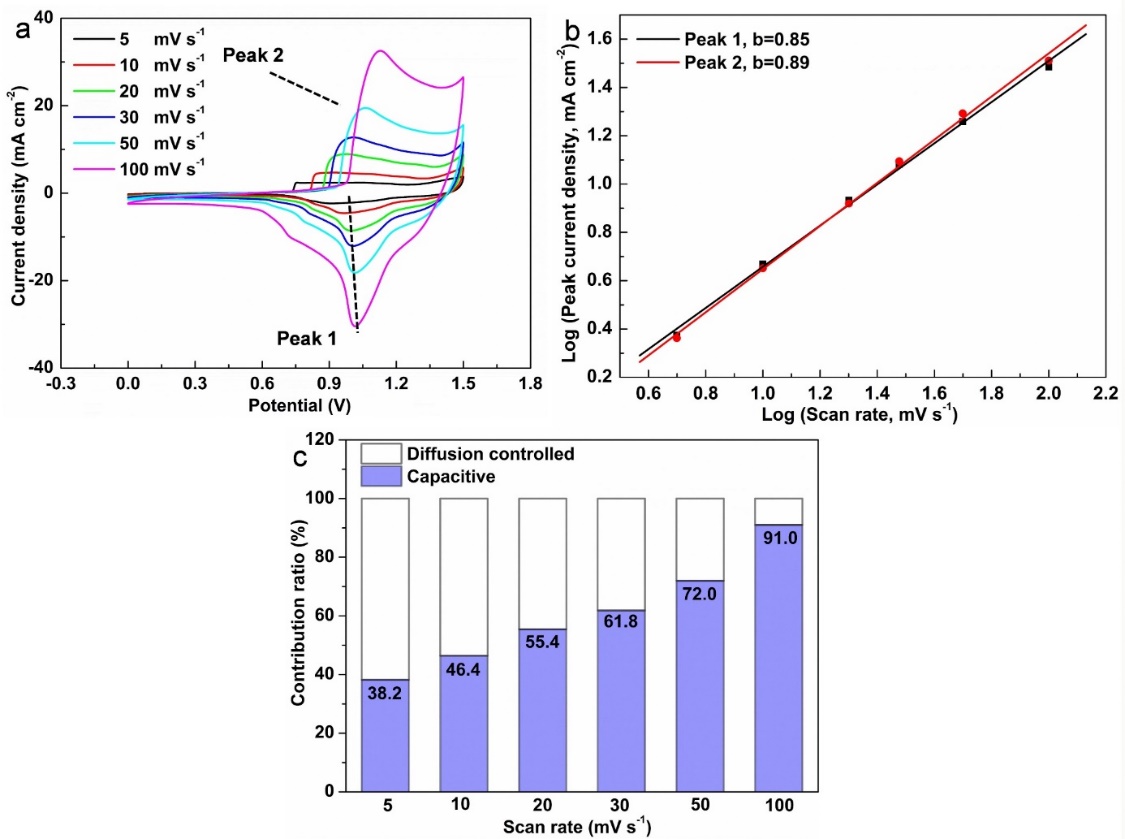


**Figure S56.** a) CV curves of the M8//AC at various scan rates of 5-100 mV s^-1^. b) Log*(i)* versus log*(v)* plots of the M8//AC at specific peak currents. c) Bar chart showing the percent of pseudocapacitive contribution of the M8//AC at different scan rates.

1. **Pseudocapacitive contribution shadow diagram of M8//AC in CV curves**


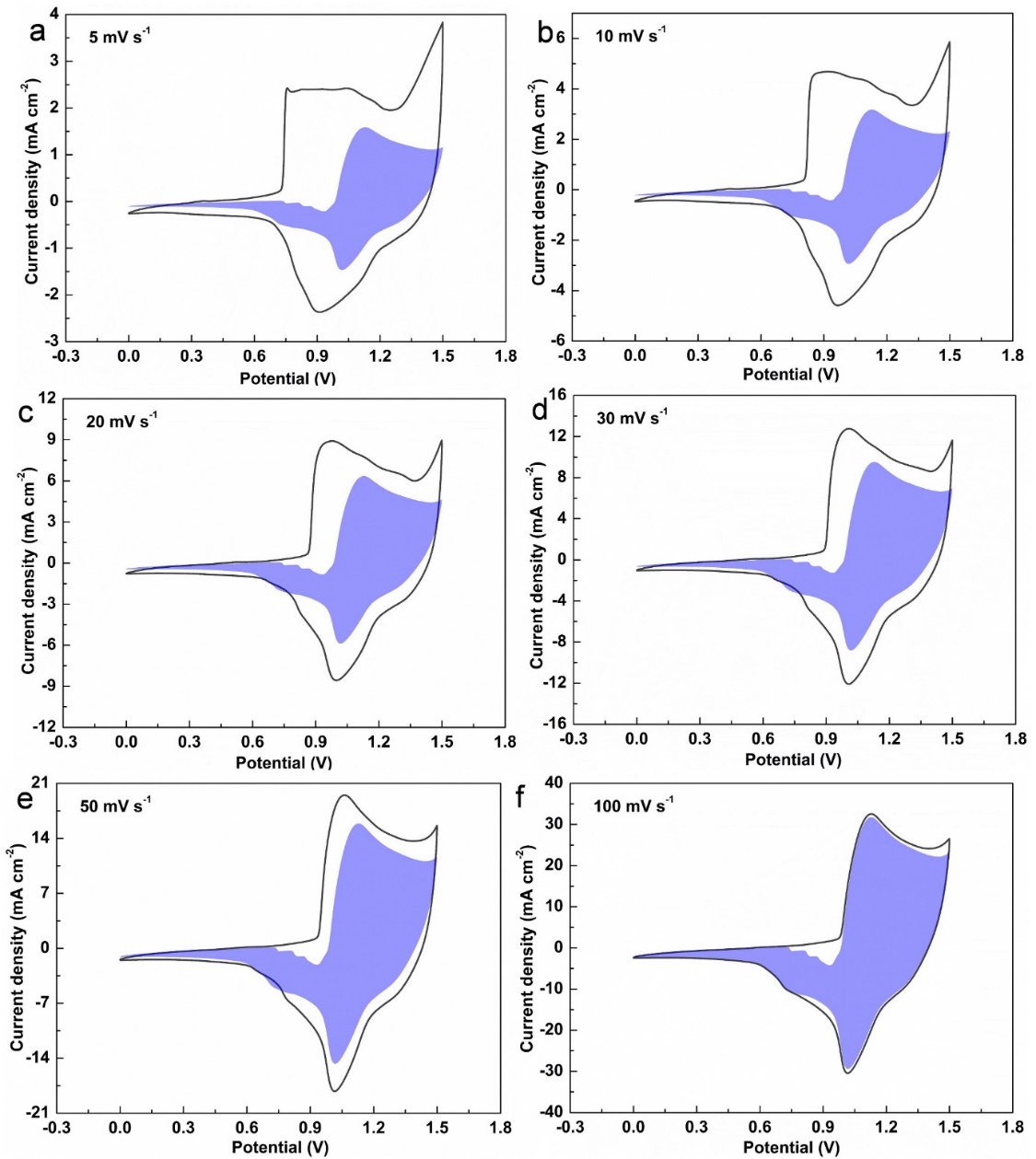


**Figure S57.** CV curve with the pseudocapacitive fraction shown by the shaded area of M8//AC at various scan rates. a) 5 mV s^-1^. b) 10 mV s^-1^. c) 20 mV s^-1^. d) 30 mV s^-1^. e) 50 mV s^-1^. f) 100 mV s^-1^.

1. **The percent of the diffusion-controlled capacity and pseudocapacitive contribution of the M1-M8//AC**


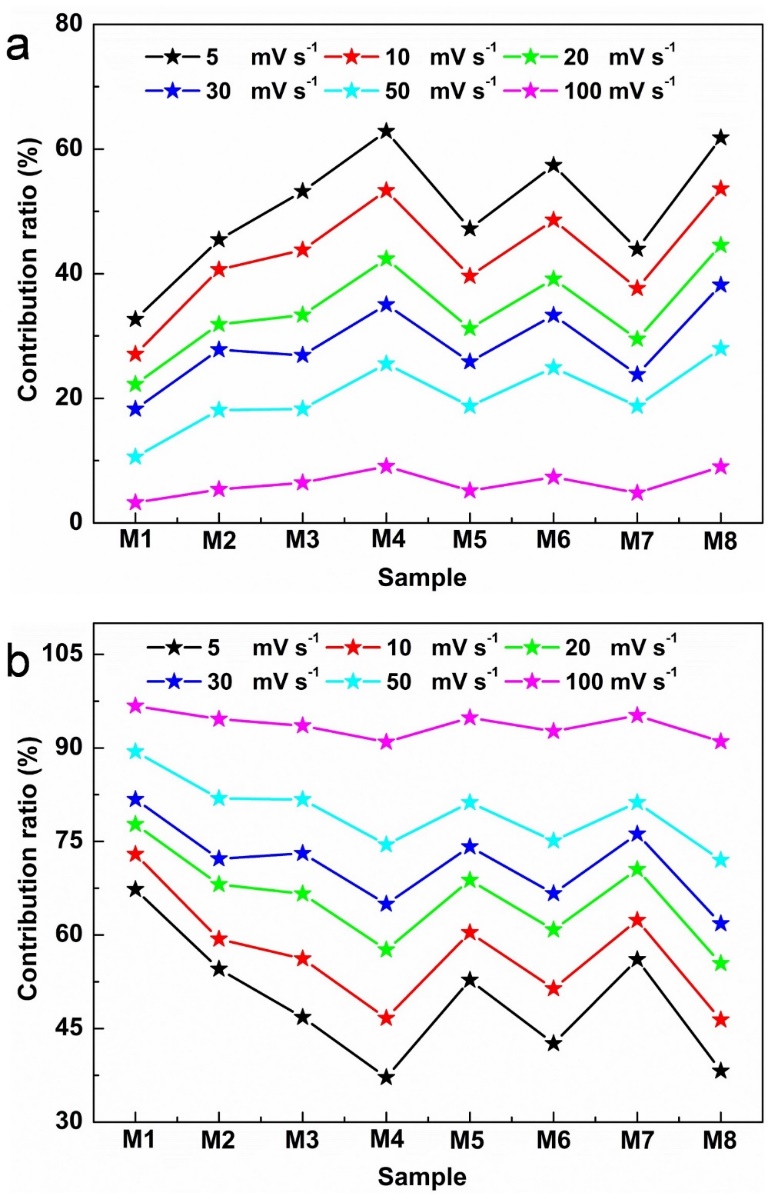


**Figure S58.** a) The percent of the diffusion-controlled capacity contribution of the M1-M8//AC at different scan rates. b) The percent of pseudocapacitive contribution of the M1-M8//AC at different scan rates.

1. **CV and GCD curves of the M5//AC at different potentials**


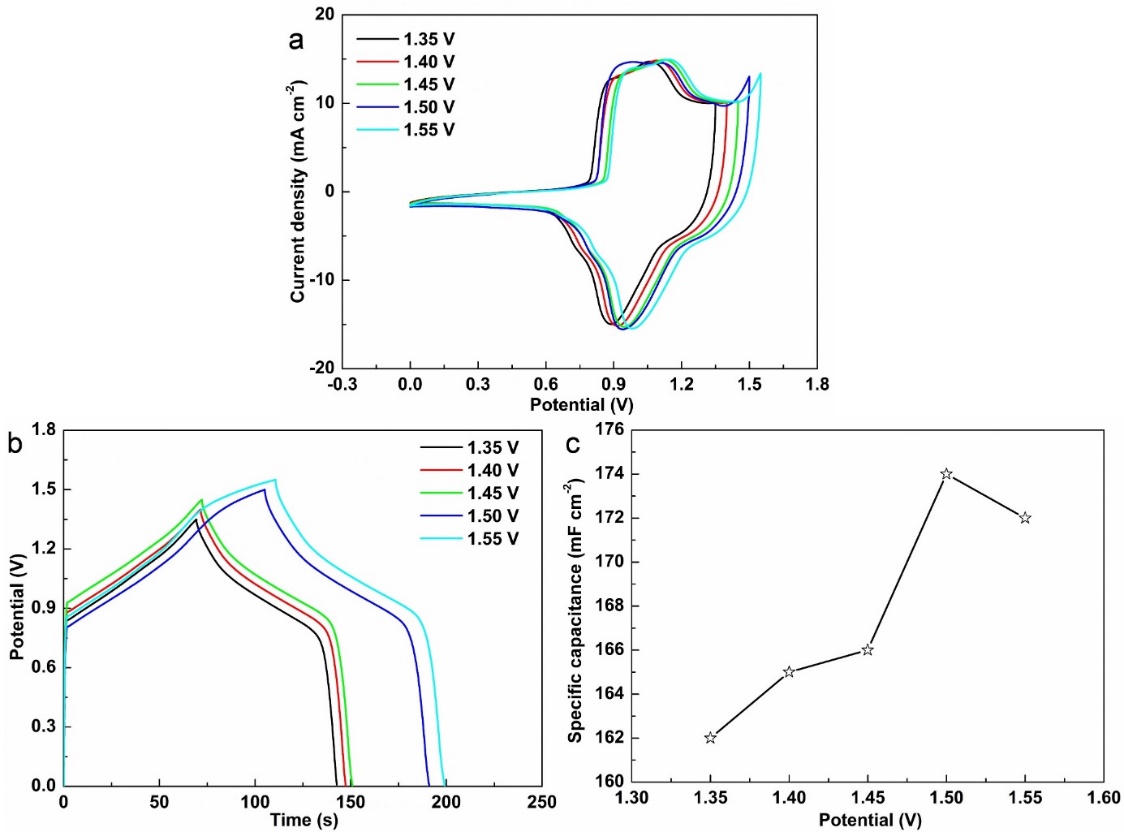


**Figure S59.** a) CV curves of the M5//AC with a scan rate at 30 mV s^-1^ at different potentials. b) The GCD curves of M5//AC with a current density 3 mA cm^-2^ at different potentials. c) Specific capacitance change vs. potential.

1. **The GCD curves of M1-M4//AC at different current densities**


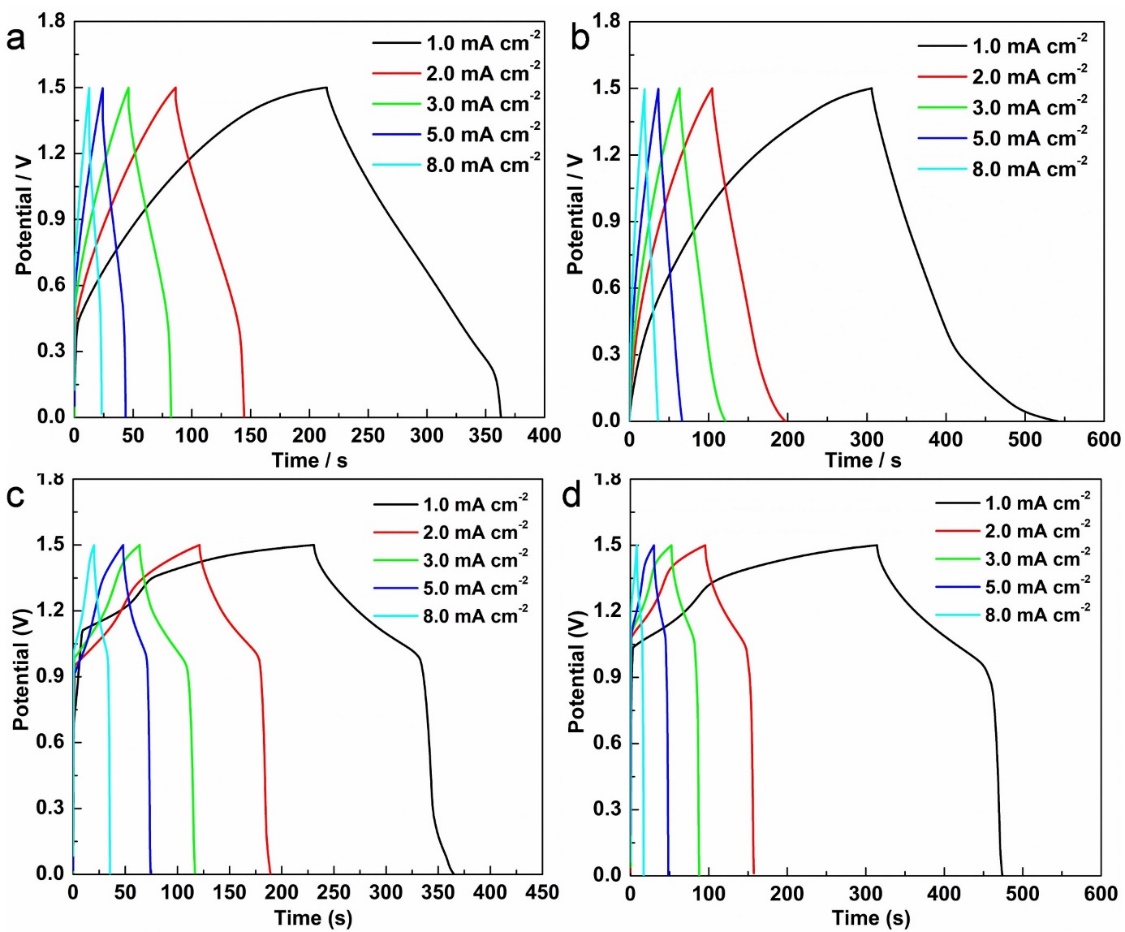


**Figure S60.** The GCD curves at different current densities. a) M1//AC. b) M2//AC. c) M3//AC. d) M4//AC.

1. **The GCD curves of M5-M8//AC at different current densities**


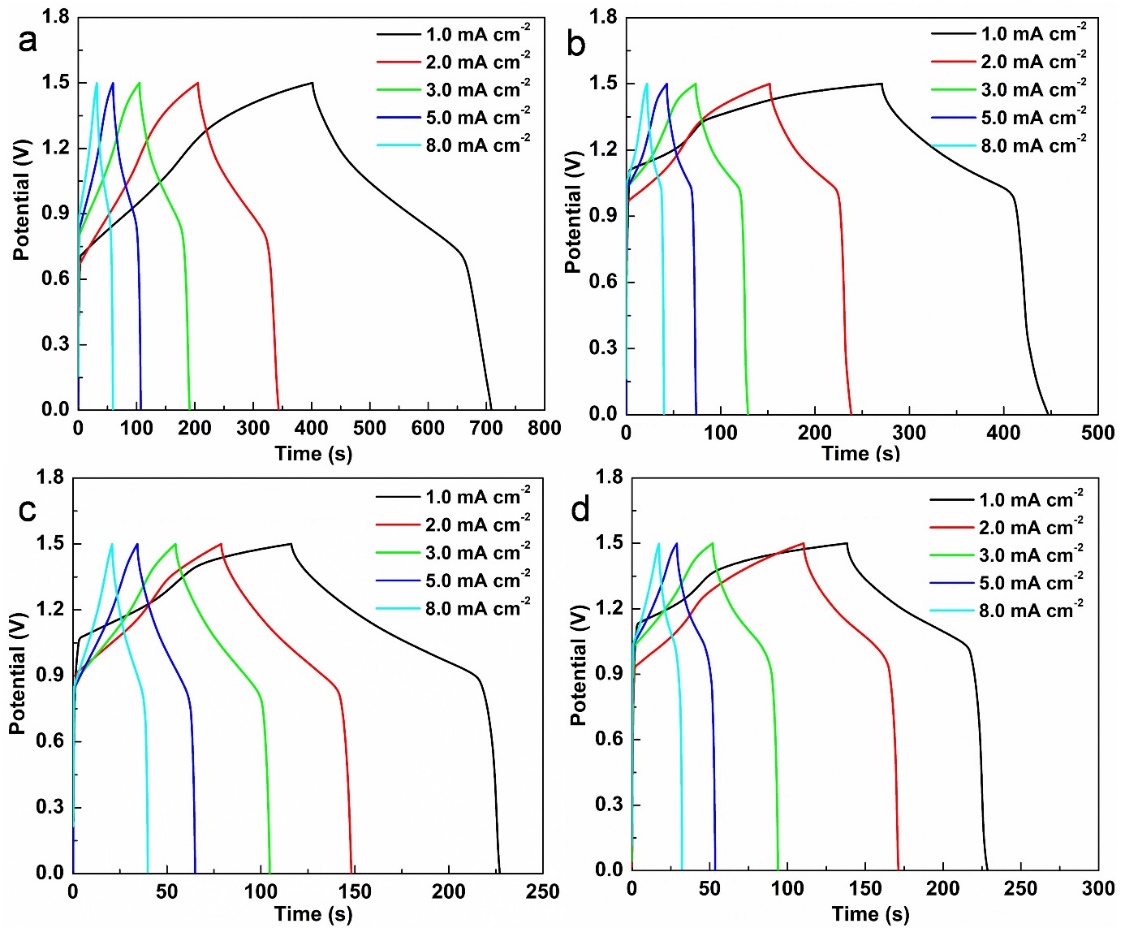


**Figure S61.** The GCD curves at different current densities. a) M5//AC. b) M6//AC. c) M7//AC. d) M8//AC.

1. **The specific capacitance of the M1-M8//AC at different current densities**


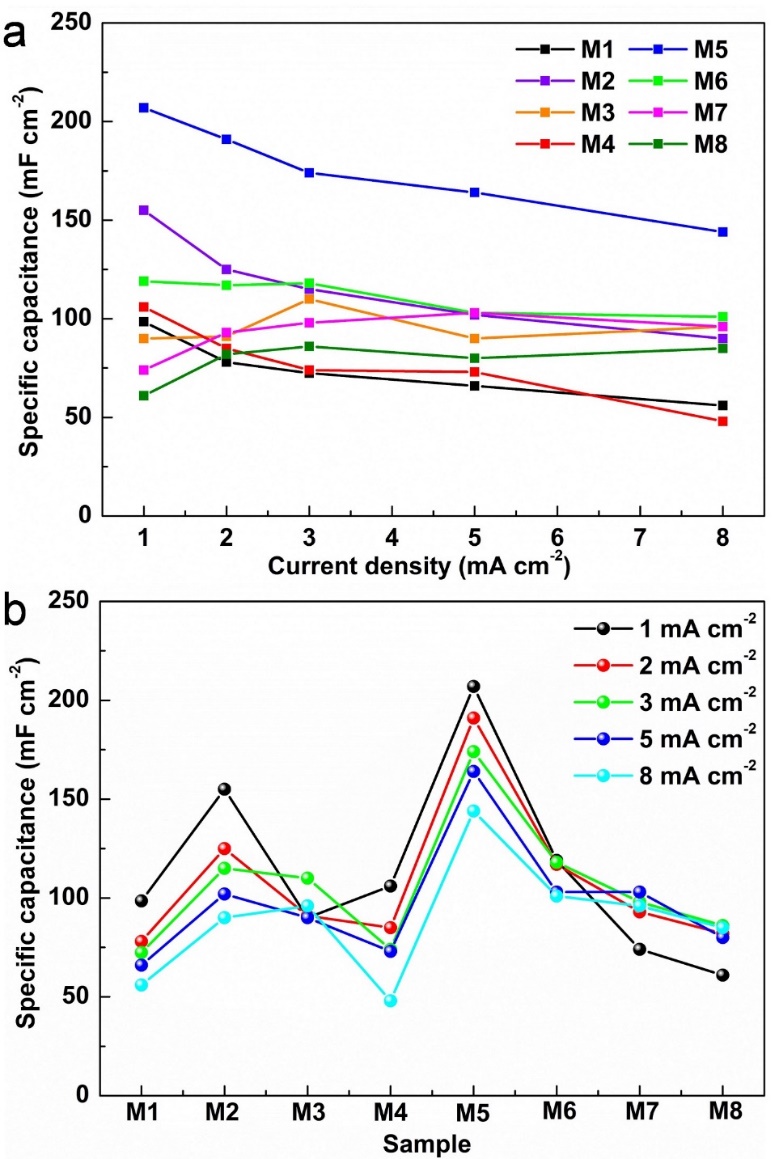


**Figure S62.** The specific capacitance of the M1-M8//AC at different current densities.

1. **Schematic diagram of ion transport pathway**

**
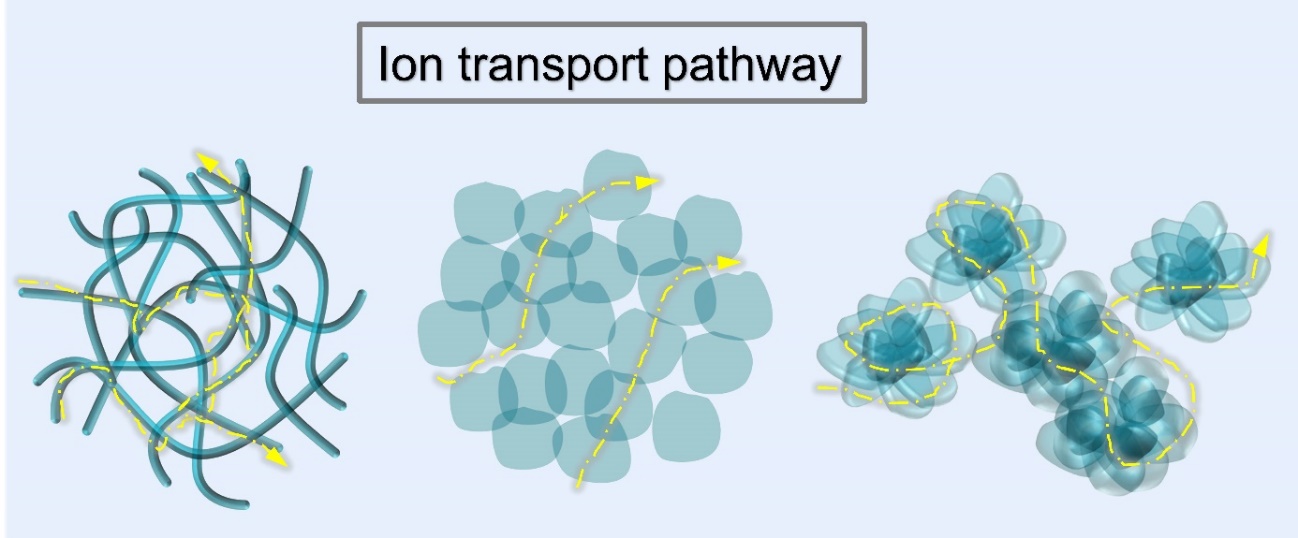
**

**Figure S63.** Schematic diagram of ion transport pathway for M1, M5, M8. (The yellow arrow represents the transmission path)

1. **The electrochemical impedance spectra of M1-M8//AC**


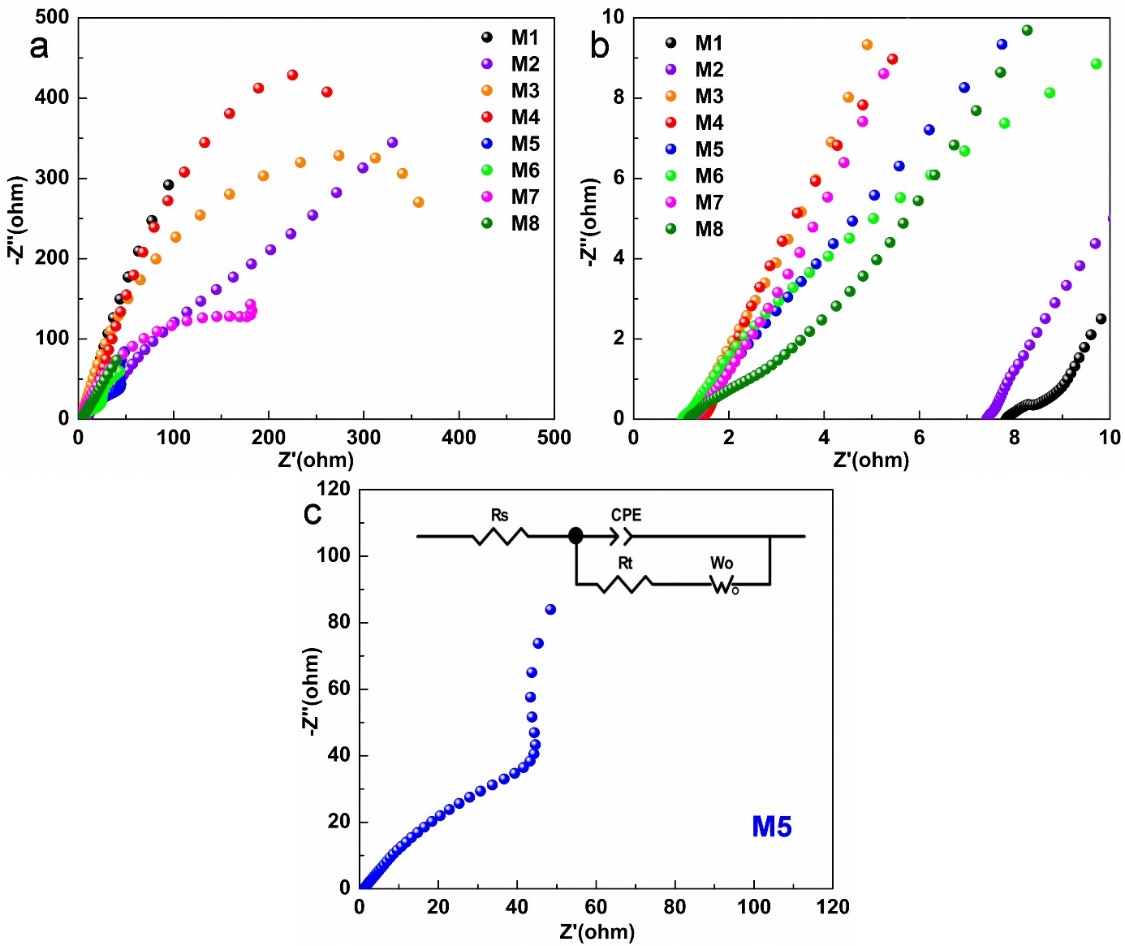


**Figure S64.** a, b) The Nyquist plots of M1-M8//AC, c) the Nyquist plot of M5//AC (inset shows the fitted equivalent circuit).

1. **The GCD curves of M5 with different mass loadings**

**
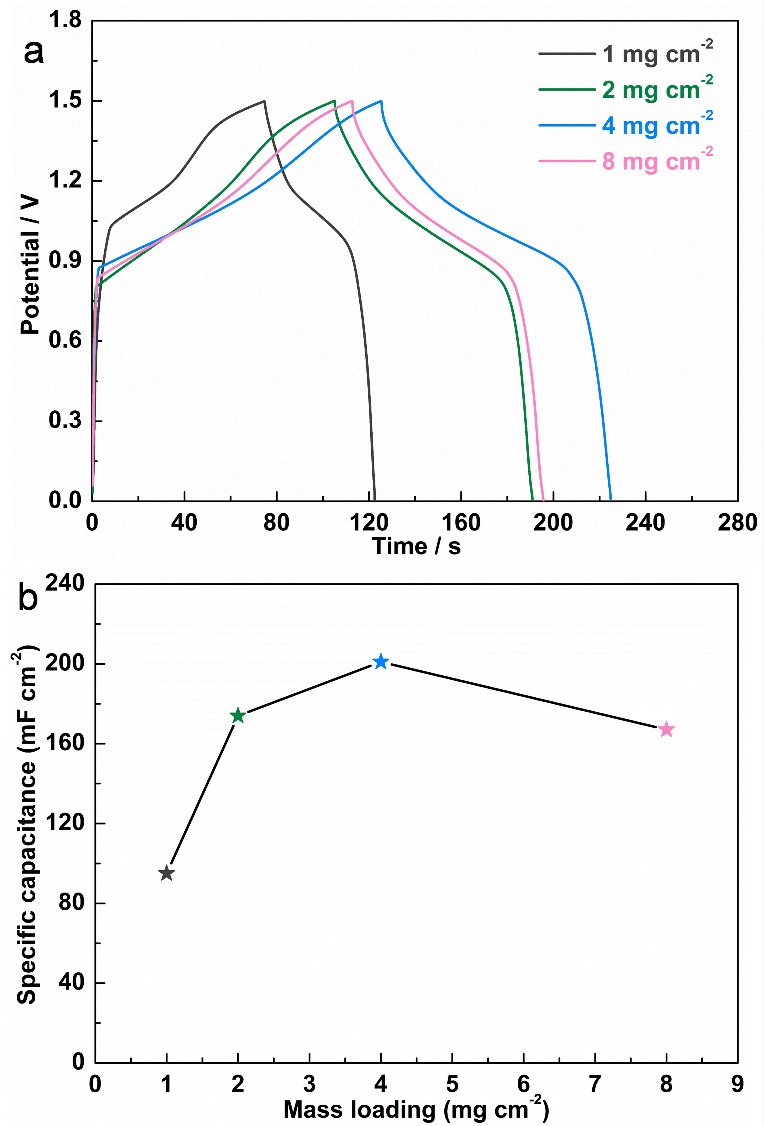
**

**Figure S65.** a) The GCD curves of M5 at 3 mA cm^-2^ with different mass loadings, b) specific capacitance at different mass loadings.

1. **The coulombic efficiency**

**

**

**Figure S66.** The coulombic efficiency of two M5//AC devices linked in series at multiple current densities.

1. **SEM images of M5 after cycling**


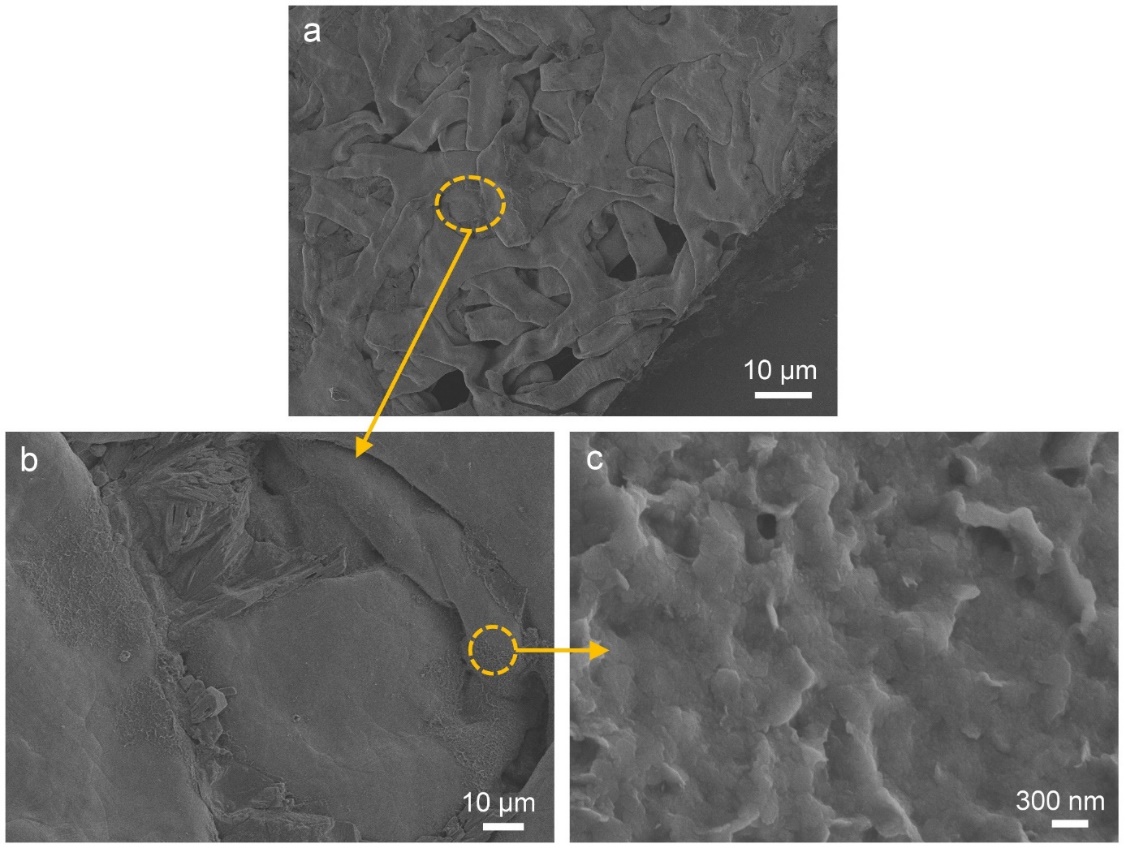


**Figure S67.** a) SEM image of M5 after cycling/NF. b) Enlarged SEM image of the selected area of the yellow dotted line in **Figure S67**a. c) Enlarged SEM image of the selected area of the yellow dotted line in **Figure S67**b.

1. **The EDS mapping images of M5 after cycling**

**
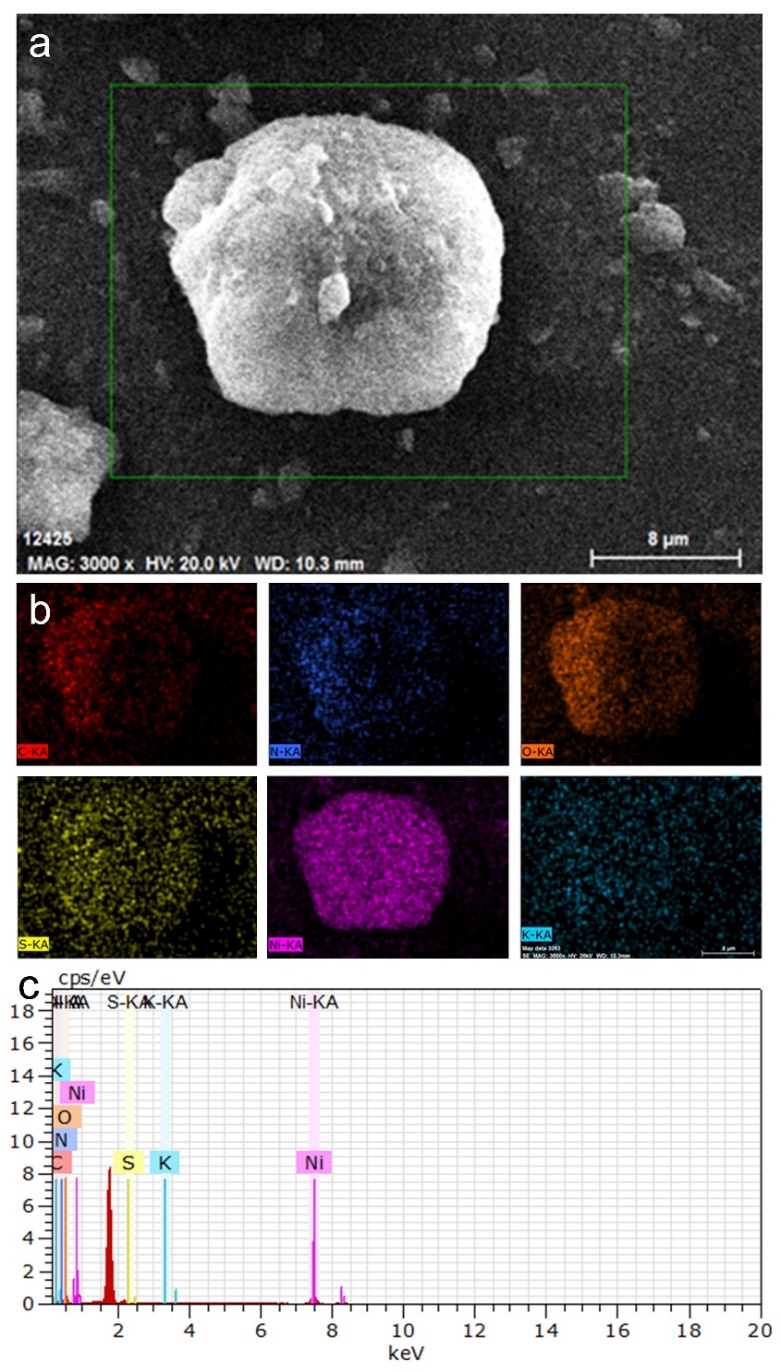
**

**Figure S68.** a) SEM image, b) the elemental mapping, and c) EDS analysis of M5 after cycling.

1. **XRD patterns of M5 after cycling**


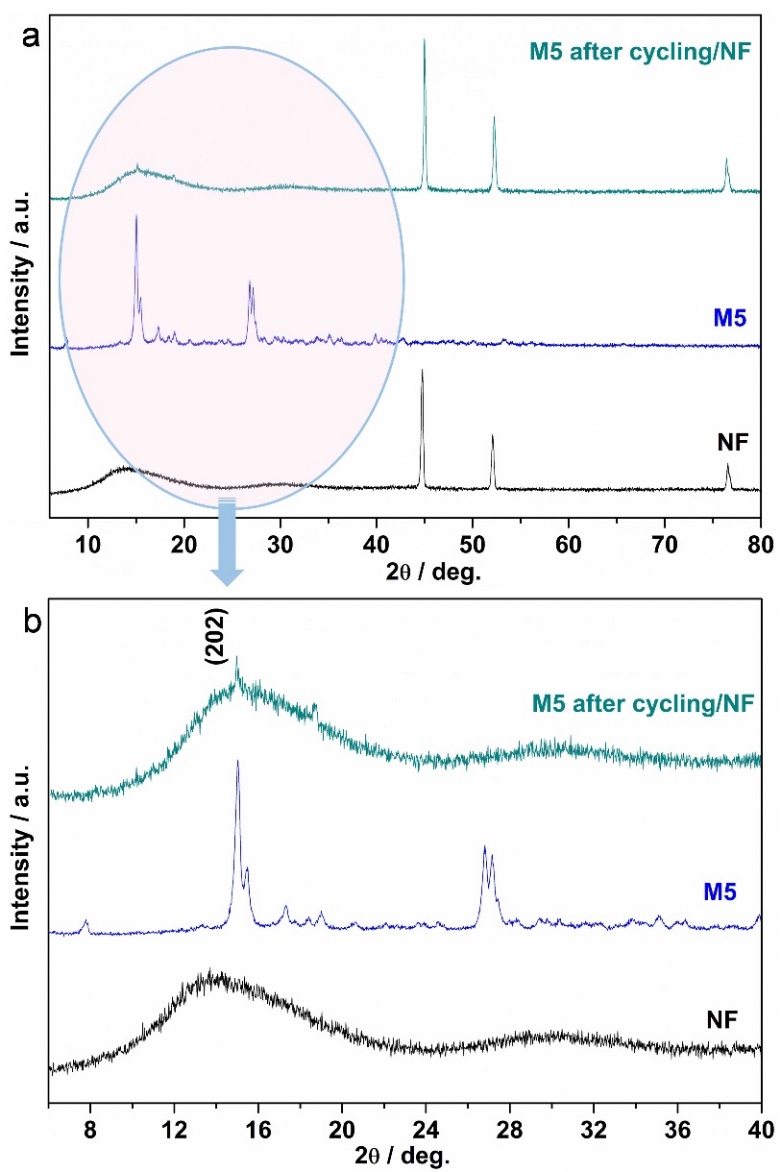


**Figure S69.** XRD patterns of NF, M5 and M5 after cycling/NF.

1. **XPS spectra of M5 after cycling**


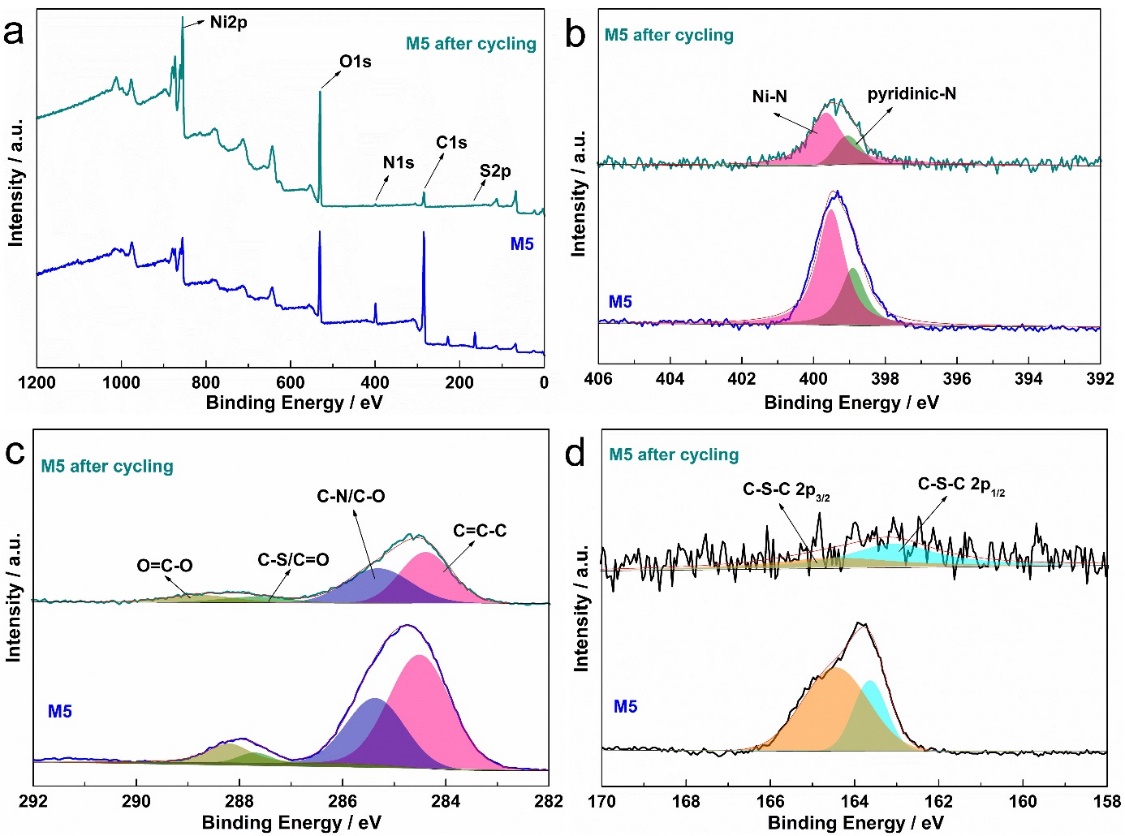


**Figure S70.** XPS spectra of the M5 and M5 after cycling. a) Survey, and high resolution b) N 1s, c) C 1s and d) S 2p XPS spectra.

1. **A comparison with previously reported MOF nanomaterials.**

Table S3. A comparison with previously reported MOF nanomaterials.

| **Material** | **SP^b)^ [F g^-1^]**  **(LCD/LSR)^c)^** | **SP^b)^ [F g^-1^]**  **(HCD/HSR)^d)^** | **Capacity retention**  **[%]** | **Electrolyte** | **Ref.** |
| --- | --- | --- | --- | --- | --- |
| Co8-MOF-5 | 0.49  (25 mV s^-1^) | 0.3  (10 mA g^-1^) | 92 (1000 cycles,  10 mA g^-1^) | 0.1 M TBAPF_6_ | [1] |
| Co-BPDC | 179.2  (10 mV s^-1^) | 60  (200 mV s^-1^) | 77.4 (1000 cycles,  100 mV s^-1^) | 0.5 M LiOH | [2] |
| Co-MOF-71 | 206.76  (0.6 A g^-1^) | — | 98.5 (1000 cycles,  —) | 1 M LiOH | [3] |
| Ni-MOF | 634  (5 mV s^-1^) | 457  (10 mV s^-1^) | 84 (2000 cycles,  50 mV s^-1^) | 6 M KOH | [4] |
| Ni_3_(btc)_2_·12H_2_O | 726  (1 A g^-1^) | 313.8  (5 A g^-1^) | 65 (5000 cycles,  1 A g^-1^) | 2 M KOH | [5] |
| Ni-DMOF-ADC | 552  (1 A g^-1^) | 438  (20 A g^-1^) | 98 (16000 cycles,  10 A g^-1^) | 2 M KOH | [6] |
| Ni_3_(HITP)_2_ | 111  (0.05 A g^-1^) | — | 90 (10000 cycles,  2 A g^-1^) | 1 M TEABF_4_/ACN | [7] |
| MIL-100 (Fe) | 39  (5 mV s^-1^) | — | — | 0.1 M K_2_SO_4_ | [8] |
| ZIF-67/GO | 202  (1 A g-1) | — | — | 6 M KOH | [9] |
| CNTs@Mn-MOF | 206  (5 mV s^-1^) | 112.5  (100 mV s^-1^) | 88 (3000 cycles,  5 A g^-1^) | 6 M KOH | [10] |
| **M5** | **612**  **(0.5 A g^-1^)** | **340**  **(10 A g^-1^)** | **95 (5000 cycles,**  **3 mA cm^-2^)** | **3 M KOH** | **Thiswork** |

a) SA: surface area; b) SP: specific capacitance; c) LCD/LSR: low current density/scan rate; d) HCD/HSR: high current density/scan rate.

1. **References**

1 Díaz R, Orcajo MG and Botas JA *et al.* Co8-MOF-5 as electrode for supercapacitors. *Mater Lett* 2012; **68**:126–128.

2 Lee DY, Shinde D V and Kim EK *et al.* Supercapacitive property of metal–organic-frameworks with different pore dimensions and morphology. *Microporous Mesoporous Mater* 2013; **171**:53–57.

3 Lee DY, Yoon SJ and Shrestha NK *et al.* Unusual energy storage and charge retention in Co-based metal–organic-frameworks. *Microporous Mesoporous Mater* 2012; **153**:163–165.

4 Liao C, Zuo Y and Zhang W *et al.* Electrochemical performance of metal-organic framework synthesized by a solvothermal method for supercapacitors. *Russ J Electrochem* 2013; **49**:983–986.

5 Kang L, Sun SX and Kong LB *et al.* Investigating metal-organic framework as a new pseudo-capacitive material for supercapacitors. *Chinese Chem Lett* 2014; **25**:957–961.

6 Qu C, Jiao Y and Zhao B *et al.* Nickel-based pillared MOFs for high-performance supercapacitors: Design, synthesis and stability study. *Nano Energy* 2016; **26**:66–73.

7 Sheberla D, Bachman JC and Elias JS *et al.* Conductive MOF electrodes for stable supercapacitors with high areal capacitance. *Nat Mater* 2017; **16**:220–224.

8 Wang R, Yan X and Lang J *et al.* A hybrid supercapacitor based on flower-like Co(OH)_2_ and urchin-like VN electrode materials. *J Mater Chem A* 2014; **2**:12724–12732.

9 Zhang W, Tan Y and Gao Y *et al.* Nanocomposites of zeolitic imidazolate frameworks on graphene oxide for pseudocapacitor applications. *J Appl Electrochem* 2016; **46**:441–450.

10 Zhang Y, Lin B and Sun Y *et al.* Carbon nanotubes@metal–organic frameworks as Mn-based symmetrical supercapacitor electrodes for enhanced charge storage. *RSC Adv* 2015; **5**:58100–58106.
